# Birth of Archaeal Cells: Molecular Phylogenetic Analyses of G1P Dehydrogenase, G3P Dehydrogenases, and Glycerol Kinase Suggest Derived Features of Archaeal Membranes Having G1P Polar Lipids

**DOI:** 10.1155/2016/1802675

**Published:** 2016-09-28

**Authors:** Shin-ichi Yokobori, Yoshiki Nakajima, Satoshi Akanuma, Akihiko Yamagishi

**Affiliations:** ^1^Laboratory of Extremophiles, Department of Applied Life Sciences, School of Life Sciences, Tokyo University of Pharmacy and Life Sciences, 1432-1 Horinouchi, Hachioji, Tokyo 192-0392, Japan; ^2^Faculty of Human Sciences, Waseda University, 2-579-15 Mikajima, Tokorozawa, Saitama 359-1192, Japan

## Abstract

Bacteria and Eukarya have cell membranes with* sn*-glycerol-3-phosphate (G3P), whereas archaeal membranes contain* sn*-glycerol-1-phosphate (G1P). Determining the time at which cells with either G3P-lipid membranes or G1P-lipid membranes appeared is important for understanding the early evolution of terrestrial life. To clarify this issue, we reconstructed molecular phylogenetic trees of G1PDH (G1P dehydrogenase; EgsA/AraM) which is responsible for G1P synthesis and G3PDHs (G3P dehydrogenase; GpsA and GlpA/GlpD) and glycerol kinase (GlpK) which is responsible for G3P synthesis. Together with the distribution of these protein-encoding genes among archaeal and bacterial groups, our phylogenetic analyses suggested that GlpA/GlpD in the Commonote (the last universal common ancestor of all extant life with a cellular form,* Commonote commonote*) acquired EgsA (G1PDH) from the archaeal common ancestor (*Commonote archaea*) and acquired GpsA and GlpK from a bacterial common ancestor (*Commonote bacteria)*. In our scenario based on this study, the Commonote probably possessed a G3P-lipid membrane synthesized enzymatically, after which the archaeal lineage acquired G1PDH followed by the replacement of a G3P-lipid membrane with a G1P-lipid membrane.

## 1. Introduction

Archaea is one of the three domains covering all extant terrestrial life. Woese et al. [1] suggested that Bacteria, Archaea, and Eukarya are distinct monophyletic groups based on the small subunit ribosomal RNA tree. However, it has been proposed that Eukarya (eukaryotes) are derived from a certain archaeal branch, such as the TACK superphylum [2] or Lokiarchaeota [3, 4]. In any of these three cases, most hypotheses placed the LUCA (last universal common ancestor), also known as the Commonote [5], between the Bacteria and a group formed by Archaea and Eukarya. We prefer to use the term “Commonote” rather than LUCA or progenote, since the definition of Commonote is the last universal common ancestor having a cellular membrane [5], as we believe that the last universal common ancestor was a cellular organism. In this article, we use the terms* Commonote commonote*,* Commonote archaea*, and* Commonote bacteria* referring to the last common ancestral species of all living organisms (formerly Commonote) and of Archaea and Bacteria, as proposed in Akanuma et al. [6].

The cell membrane component of* C. commonote,* and before the appearance of* C. bacteria* and* C. archaea,* are foci for the early evolution of terrestrial life, because membrane lipids that divide inside and outside of the cell are essential for life [7, 8].

**Figure 1:**
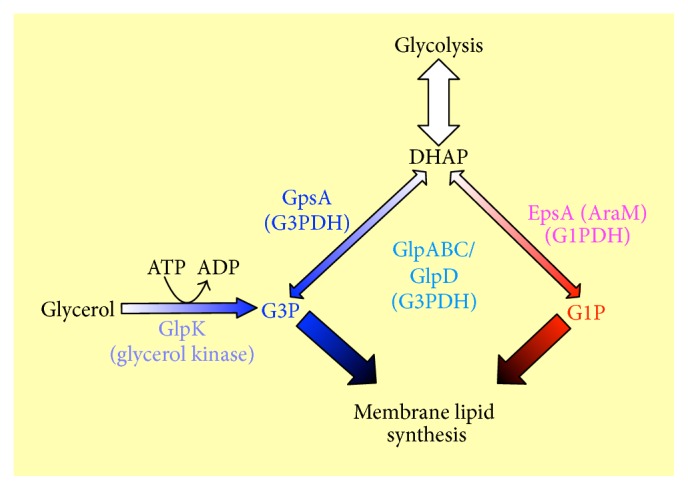
Overview of the stereospecific biosynthetic pathways of G1P and G3P.

Various lipid structures are found in the three domains. For example, as major membrane lipids, Bacteria and Eukarya have ester lipids with long chain fatty acids, whereas Archaea have ether lipids with isoprenoids as their hydrophobic moiety. However, all cellular organisms have polar lipids with a glycerol backbone as a common structure, with the exception of their stereostructures. The stereostructure of the glycerol backbone in the polar lipids of Bacteria and Eukarya is* sn*-glycerol-3-phosphate (G3P), while being* sn*-glycerol-1-phosphate (G1P) in Archaea [8, 9]. G3P and G1P are generated from dihydroxyacetone phosphate (DHAP) by different enzymes: G3P dehydrogenase (G3PDH) and G1P dehydrogenase (G1PDH), respectively (Figure 1) [8–11]. In addition, G3P can be obtained from glycerol by phosphorylation by glycerol kinase (GK) (Figure 1) [12]. Note that although ether lipids are major membrane lipids that are often referred to as a unique characteristic of archaeal cells, various (thermophilic) bacterial cells are also known to contain ether lipids in their cellular membranes (e.g., [13, 14]).

In Bacteria, G3PDH encoded by the* gpsA* gene, which is NAD^+^ dependent, is responsible for the stereospecific synthesis of G3P from DHAP (Figure 1) [10]. In Eukarya, G3PDH encoded by the* gpd* gene [11], which is cytoplasmic and NAD^+^ dependent, and eukaryal homolog of* gpsA*, is responsible for the stereospecific synthesis of G3P from DHAP. Only a few* gpsA*/*gps* gene homologs are known in Archaea. For example,* Archaeoglobus fulgidus* has a GpsA homolog; however it prefers NADP^+^ rather than NAD^+^ [15].

In addition to the product of the* gpd* gene, flavin-dependent mitochondrial dehydrogenase, encoded by the* glp *gene, synthesizes G3P from DHAP during the “GP shuttle” in eukaryal cells such as insect flight muscle cells [16, 17]. Certain heterotrophic bacteria also have* glp* homologs (*glpA*/*glpD*) [12]. GlpA, the product of the* glpA *gene, is a subunit of an anaerobic G3PDH, GlpABC. GlpD, the product of the* glpD *gene, is a dimeric protein that is called an aerobic G3PDH. The anaerobic and aerobic G3PDHs catalyze G3P from DHAP during glycerol metabolism. Because DHAP is intermediate in glycolysis, G3P can be used for various metabolic pathways via glycolysis. Furthermore, GlpK, the product of the* glpK* gene and an ATP-dependent glycerol kinase found in various bacteria, synthesizes G3P from glycerol directly [12]. The pathway from glycerol to DHAP catalyzed by glycerol kinase and G3PDH is the first step in glycerol fermentation [12].

In Archaea, G1PDH encoded by the* egsA* gene, which is NADH-dependent, is responsible for the stereospecific synthesis of G1P from DHAP (Figure 1) [8, 9]. Proteins with G1PDH activity have been reported from certain bacterial lineages, such as firmicute* Bacillus subtilis* [18, 19]. Guldan et al. [18] reported that* B. subtilis* AraM, which is in an “Ara operon,” has G1PDH activity. In addition,* B. subtilis *AraM has a 31% sequence identity with* Archaeoglobus fulgidus* EgsA (G1PDH); therefore the bacterial AraM could be a bacterial EgsA homolog. G1P indeed becomes part of an archaea-type ether lipid heptaprenylglyceryl phosphate in* B. subtilis*. However, its function is still unknown [19]. Had they not originated by horizontal gene transfer from archaeal species after the separation of Bacteria and Archaea, the common ancestor of Bacteria and Archaea (or LUCA/Commonote) could have had G1P as a membrane component.

On the other hand, proteins with G3PDH activity have also been reported from certain archaeal lineages [20]. Rawls et al. [20] suggested that* Halobacterium* and some other archaeal species have GlpA/GlpD type G3PDH. In addition, some archaeal species such as* Archaeoglobus fulgidus* harbor* gpsA* gene [15, 21]. If they had not originated by horizontal gene transfer from bacterial species after the separation of Bacteria and Archaea, the common ancestor of Bacteria and Archaea (or LUCA/Commonote) could have had G3P as a membrane component.

**Figure 2:**
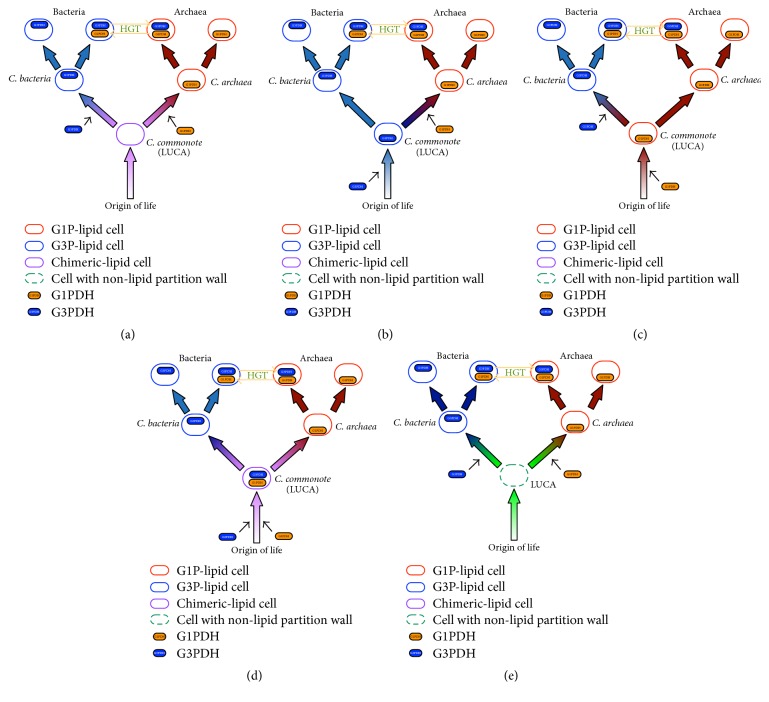
Five hypotheses regarding the early evolution of cell membrane glycerolipid backbone. LUCA: last universal common ancestor.* C. commonote* was defined as the cellular last universal common ancestor [5]. (a) The* C. commonote* had a heterochiral polar lipid membrane. Both G1P and G3P were used, but they might have been synthesized via a nonenzymatic pathway (absence of G1PDH and G3PDH in* C. commonote* cell) or via an enzymatic pathway (certain enzymes did not have specificity to either G1P or G3P; both G1P and G3P were created by a single enzyme). Then,* C. bacteria* acquired G3PDH and acquired a G3P-homochiral polar lipid membrane. On the other hand, the archaeal common ancestor acquired G1PDH with a G1P-homochiral polar lipid membrane. (b) The* C. commonote* had G3PDH. Therefore,* C. commonote* had a G3P-homochiral polar lipid membrane.* C. archaea* acquired G1PDH, and then the G3P-homochiral polar lipid membrane was replaced by a G1P-homochiral polar lipid membrane. (c)* C. commonote* had G1PDH. Therefore,* C. commonote* had a G1P-homochiral polar lipid membrane.* C. bacteria* acquired G3PDH, and then the G1P-homochiral polar lipid membrane was replaced by a G3P-homochiral polar lipid membrane. (d)* C. commonote* had both G1PDH and G3PDH. Therefore,* C. commonote* had a heterochiral polar lipid membrane (G1PDH created G1P and G3PDH created G3P). In the bacterial line, G1PDH was lost. Then, Bacteria acquired a G3P-homochiral polar lipid membrane. G3PDH was then lost in the archaeal line. Archaea then acquired a G1P-homochiral polar lipid membrane. (e) The LUCA did not have membrane structure. (The* C. commonote* is cellular LUCA. Therefore, we do not use the term “*C. commonote*” for this hypothesis.) The bacterial line then acquired G3PDH and acquired a G3P-homochiral polar lipid membrane. The archaeal line also acquired G1PDH and acquired a G1P-homochiral polar lipid membrane.

There is no sequence similarity between G3PDH (*gpsA*/*gpd*,* glpA*/*glpD*/*glp*) and G1PDH (*egsA*) at the gene or protein level [9]. Koga et al. [9] hypothesized that the separation of Bacteria and Archaea might have been caused by cellularization by membranes with two enantiomeric lipids synthesized by G3PDH and G1PDH, respectively, which evolved from different enzymes (Figure 2(a)).

Wächtershäuser [22] proposed a model incorporating Koga's model [9] and the precell theory [23]. According to his hypothesis, in the earliest stage the precell had heterochiral membrane lipids. The heterochiral membrane slowly segregated to form a stable homochiral membrane at an early point in the evolution of life. Wächtershäuser proposed that the heterochiral membrane evolved toward a homochiral membrane, assuming that the homochiral membrane is more stable than the heterochiral membrane. Bacteria emerged from precells with G3P-lipid rich membranes through the appearance of G3PDH, and Archaea emerged from precells with G1P-lipid rich membranes through the appearance of G1PDH. Wächtershäuser's hypothesis is summarized in Figure 2(a). Peretó et al. [7] proposed a model that is also summarized in Figure 2(a). In their model, LUCA (*C. commonote*) had a heterochiral membrane, and G1P and G3P were synthesized by an unknown enzyme that did not distinguish G1P and G3P.

There are four other possible scenarios (Figures 2(b)–2(e)). The* C. commonote* may have had either G3PDH or G1PDH or both. These cases are summarized in Figures 2(b)–2(d). In addition, Martin and Russell [24] hypothesized that Bacteria and Archaea emerged independently from a universal ancestor that was a non-free-living cell in the iron monosulfide compartments (Figure 2(e)).

Molecular phylogenetic studies of G1PDH and G3PDH have been performed. In a phylogenetic analysis of G1PDH by Daiyasu et al. [25], the archaeal G1PDHs form a group with some bacterial sequences including* B. subtilis* AraM. In their tree, archaeal G1PDHs form subgroups of bacterial AraM, although the authors did not point this out, apparently. In Carbone et al. [26], the archaeal G1PDHs appeared as subgroups of bacterial G1PDHs, similar to the results of Daiyasu et al. [25]; however no detailed phylogenetic analysis was presented in this article. According to Peretó et al. [7], bacterial G1PDH and archaeal G1PDH form monophyletic groups. However, because of the limited number of bacterial G1PDH sequences analyzed, it was difficult to determine the phylogenetic position of bacterial G1PDH. In their analysis of G3PDH phylogeny [7], the archaeal G3PDH reported by Rawls et al. [20] was not included.

To understand the early evolution of cellular membranes, we reconstructed separate molecular phylogenetic trees for G1PDH (EgsA/AraM), G3PDH (GpsA), G3PDH (GlpA/GlpD), and GK (GlpK). Together with current knowledge of the distributions of these proteins among archaeal and bacterial groups, we discuss below a scenario of early evolution of cellular membranes.

## 2. Materials and Methods

### 2.1. Phylogenetic Analyses of G1PDH

For the phylogenetic analysis, G1PDH and its family proteins, G1PDH (egsA), Glycerol dehydrogenase (GDH), 3-dehydroquinate synthase (DHQS), and alcohol dehydrogenase (ALDH), 2,335 entries in total were retrieved from GenBank by a BLAST search using* Sulfolobus tokodaii* G1PDH (DDBJ/GenBank/EMBL accession number P58460) as the key sequence by the end of 2012. The retrieved entries were aligned by MAFFT version 6.814b [27] with the -auto option, followed by manual editing. After removing sequences that were not well aligned and were fast-evolving, alignment consisting of 182 sequences was made. The list of these sequences can be found in Supplementary Table S1 in Supplementary Material available online at http://dx.doi.org/10.1155/2016/1802675.

The well conserved regions were selected using TrimAL version 1.4 beta [28] with the -automated1 option. Then, by using TrimAL with -nogaps option, the gap-containing sites were removed. The resultant trimmed Multiple Sequence Alignment (MSA) used in further phylogenetic analyses is shown in Supplementary Figure S1. The abstracted alignment of G1PDH without trimming by TrimAL is also shown in Supplementary Figure S2.

Molecular phylogenetic analyses were performed using the Maximum Likelihood (ML) method and the Bayesian Inference (BI) method. The ML tree was constructed with RAxML version 7.4.2 [29] via RAxML GUI version 1.3 [30] with the PROTGAMMALG model. The evolutionary model was selected by comparing the AIC estimated by ProtTest version 3.2 [31]. The BI tree (posterior-probability consensus tree inferred with Bayesian Inference) was constructed using PhyloBayes version 3.2f [32] with the CAT-Poisson + Γ(4) model (NCAT: C20, Gamma distribution: 4 rate categories, MCMC: 200,000 cycles, tree sampling: every 10 cycles, burn-in: first 50,000 cycles, and running chain: 2 chains). In both cases, GDH, DHQS, and ALDH were treated as the outgroup. FigTree version 1.4.2 [33] was used to display the trees.

In addition, the approximately unbiased (AU) test [34] was performed with Consel v0.1j [35] to test various alternative phylogenetic hypotheses. Based on the ML tree of G1PDH inferred by the RAxML, we divided G1PDHs into 8 groups,* Thermofilum pendens* Hrk-5 (Thermoproteales of Crenarchaeota) (Tpe), the rest of Thermoproteales (THER), Desulfurococcales + Acidilobales + Sulfolobales (DAS), Thaumarchaeota (THAU), Euryarchaeota (EURY),* Bacillus subtilis* subsp.* subtilis* str. 168 (Bsu), Deltaproteobacteria + Haloplasmatales +* Anoxybacillus flavithermus* WK1 +* Bacillus cellulosilyticus *DSM 2522 (DHF), and Gammaproteobacteria + Actinobacteria (GA), together with outgroup (OG). Under the two constraint conditions ({{Tpe, Bsu, DHF, GA}, THER, DAS, THAU, EURY, OG} and {Tpe, THER, DAS, THAU, EURY, {Bsu, DHF, GA, OG}}), we listed 3,150 relationships among 8 G1PDH groups and 1 outgroup, using Protml of Molphy 3.2b [36]. Next, the 3,150 relationships were used as constraints for an ML tree search performed using RAxML with the PROTGAMMALG model. The log likelihood of 3,150 resultant trees was compared, and the top 2,000 trees on the log likelihood were then used for the AU test with Consel.

### 2.2. Phylogenetic Analyses of G3PDH (GpsA)

The sequences of GpsA and its related proteins were retrieved via keywords search on GenBank using the terms “GpsA”, “hydroxyacyl-CoA dehydrogenase (HACDH)”, and “UDP-glucose 6-dehydrogenase (UDPGDH)”, on May 15, 2015. Initial alignment was done with MAFFT followed by manual editing. After removing the sequences that were not well aligned and were fast-evolving, alignment consisting of 305 sequences was made. HACDH and UDPGDH were used as outgroups of GpsA. The list of these 305 sequences can be found in Supplementary Table S2. The trimmed MSA used in further phylogenetic analyses is shown in Supplementary Figure S3. The abstracted alignment of G3PDH (GpsA) without trimming by TrimAL is also shown in Supplementary Figure S4. Molecular phylogenetic analysis of G3PDH (GpsA) was done as described in Section 2.1.

### 2.3. Phylogenetic Analyses of G3PDH (GlpA/GlpD)

The sequences of GlpA and its homologs were retrieved via the following processes by the end of 2012:

1. Retrieving sequences by a BLAST search [39] on GenBank (http://blast.ncbi.nlm.nih.gov/Blast.cgi).
2. Retrieving archaeal sequences in KEGG Orthology KO00111 (GlpA, GlpD) (http://www.genome.jp/dbget-bin/www_bget?ko:K00111).
3. Retrieving sequences used in Rawls et al. [20].

A BLAST search was carried out for each phylum (subphylum for Proteobacteria) of Bacteria and Archaea. The key sequence for the BLAST search was* Haloferax volcanii* GlpA (YP_003535585). We selected 5,314 sequences in total.

The alignment was done using MAFFT followed by manual editing. After removing the sequences that were not well aligned and were fast-evolving, an alignment consisting of 286 sequences was constructed. The outgroup of this analysis consisted of FAD-dependent oxidoreductase for which no function was known, according to the analysis done by Rawls et al. [20].

In preliminary analyses, we also used D-amino acid oxidases and D-amino acid deoxidases as the part of the outgroup. However, the FAD-dependent oxidoreductase sequences were the closest outgroup to the groups of GlpA/GlpD sequences in which all bacterial GlpA/GlpD sequences were included. Therefore, we used only the FAD-dependent oxidoreductase sequences as the outgroup of GlpA/GlpD. The list of these 286 sequences is shown in Supplementary Table S3. Although some genes were annotated to be anaerobic G3PDH (e.g., WP_011250344 for* Thermococcus kodakaraensis*) in the genomes of thermococcal and some other crenarchaeal species, they were not clustered with other G3PDHs, but with FAD-dependent oxidoreductase sequences. Therefore, in this paper, these genes annotated to be anaerobic G3PDH were excluded from our G3PDH analysis. The trimmed MSA used in phylogenetic analyses is shown in Supplementary Figure S5. The abstracted alignment of G3PDH (GlpA/GlpD) without trimming by TrimAL is also shown in Supplementary Figure S6. The molecular phylogenetic analysis of G3PDH (GlpA/GlpD) was carried out as described above.

### 2.4. Phylogenetic Analyses of Glycerol Kinase (GlpK) Catalyzing Formation of G3P from Glycerol

GlpK sequences were collected by a keyword search (with GlpK as the keyword) and a BLAST search (blastP). The BLAST search was done using* Escherichia coli* glycerol kinase (AAB03058) as a key sequence against the NCBI protein database on July 2, 2015. Ca. 48,000 entries were retrieved in total. After removing duplicated entries, we aligned the sequences using MAFFT and constructed a preliminary phylogenetic tree using FastTree 2.1.5 [40, 41] on Geneious R8.1 [42]. After further removal of sequences that were not suitable for further analyses, we selected 374 sequences. As the outgroup for the phylogenetic analyses of GlpK, we used xylulose kinase and carbohydrate kinase sequences. The list of these 374 sequences is shown in Supplementary Table S4. The trimmed MSA used in further phylogenetic analyses is shown in Supplementary Figure S7. The abstracted alignment of glycerol kinase (GlpK) without trimming by TrimAL is also shown in Supplementary Figure S8. The molecular phylogenetic analysis of GlpK was done as described in Section 2.1.

### 2.5. Distribution of EgsA/AraM, GpsA, GlpA/GlpD, and GlpK among Archaeal and Bacterial Taxonomic Groups

To clarify the distribution of EgsA/AraM, GpsA, GlpA/GlpD, and GlpK among archaeal and bacterial groups, BLASTP and TBLASTX searches of these proteins for each archaeal/bacterial group were conducted. As the key sequences, P58460 (*Sulfolobus tokodaii*) and NP_390754 (*Bacillus subtilis* subsp.* subtilis* str. 168) (EgsA/AraM), WP_010878372 (*Archaeoglobus fulgidus*) and AAB18585 (*Escherichia coli* str. K-12 substr. MG1655) (GpsA), YP_004342538 (*Archaeoglobus veneficus*) and ZP_03590616 (*B. subtilis *subsp.* subtilis* str. 168) (GlpA/GlpD), and AAB90370 (*Archaeoglobus fulgidus* DSM 4304) and AAB03058 (*E. coli* str. K-12 substr. MG1655) (GlpK) were used. The BLASTP search was performed on June 3 and September 7 and 8, 2015, and the TBLASTX search was performed on September 7–11, 2015 against the nonredundant database of GenBank, NCBI.

The results of our BLAST searches were then compared with the lists of genes (EgsA/AraM, GpsA, GlpA/GlpD, and GlpK) in KEGG Orthology (release 76.0) [37].

## 3. Results and Discussion

### 3.1. Phylogenetic Analysis of G1PDH

**Figure 3:**
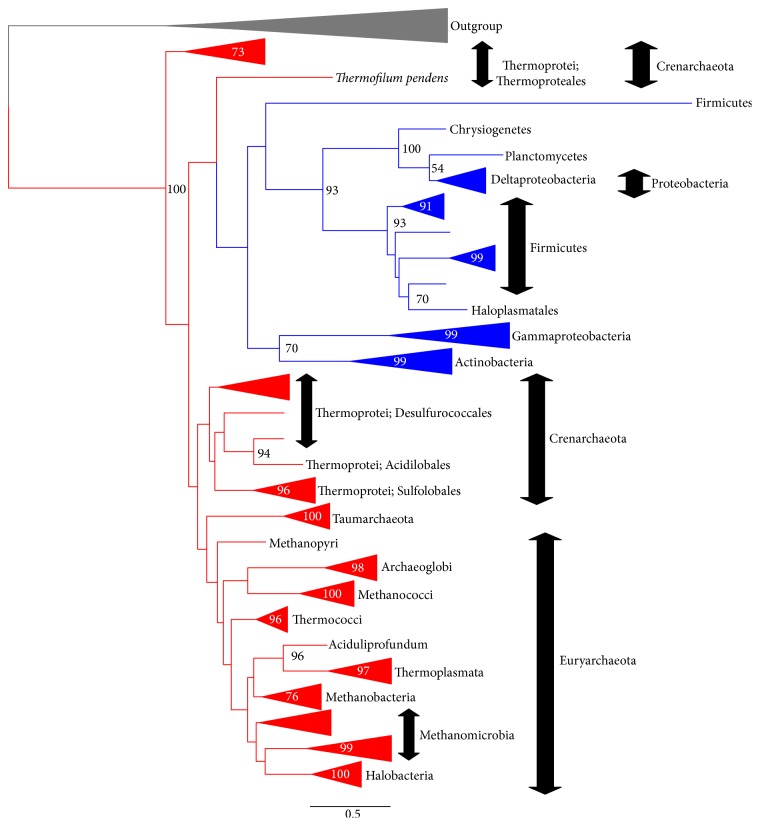
Outline of the G1PDH (EgsA/AraM) tree (ML method). The tree was constructed using RAxML version 7.4.2 [29] with the PROTGAMMALG model. The alignment with 182 OTUs and with 252 sites without any indels was used. The bootstrap analysis was carried out with 100 resamplings (slow option). The log likelihood of this tree is −44885.4. The bootstrap probability (BP) larger than 50% is shown at each node of the tree. The monophyletic group consisting of the same taxonomic group is shown in a simplified presentation. For the details of this tree, see Supplementary Figure S9.

Figure 3 shows an outline of the ML tree of G1PDH (EgsA/AraM). A detailed version of this tree can be found in Supplementary Figure S9. In this tree, Crenarchaeal G1PDHs appear as the basal branches of G1PDH. Euryarchaeal, thaumarchaeal, and bacterial G1PDHs appear as subgroups of crenarchaeal G1PDHs. Although euryarchaeal and thaumarchaeal G1PDHs form a group in this tree, 31 bacterial G1PDHs form a distinct monophyletic group separated from the G1PDHs of euryarchaeal and thaumarchaeal ones.* Bacillus subtilis* “G1PDH,” which has been biochemically characterized [18, 19], also appears here. The bacterial G1PDHs appear as a sister group of Crenarchaeon* Thermofilum pendens* G1PDH (Figure 3). The group consisting of bacterial and* T. pendens* G1PDHs is the second basal group.

In our AU test [34] for these G1PDH trees, the largest AU value indicating the bacterial G1PDHs belong to a subgroup of archaeal G1PDHs is 0.733, whereas the largest AU value indicating the bacterial G1PDHs form a distinct group from the archaeal G1PDHs is 0.301 (Supplementary Table S5). Although the AU test suggests that we cannot reject the hypothesis that* C. commonote* had G1PDH, it is more likely that G1PDH was acquired by* C. archaea*.

We also performed a BI analysis using PhyloBayes under the CAT-Poisson (C20) + G(4) model (Supplementary Figure S10). In the BI tree (the posterior-probability (PP) consensus tree of BI analysis), G1PDHs of Crenarchaea appeared as the paraphyletic group. G1PDHs of Thaumarchaea and Euryarchaea formed a monophyletic subgroup in G1PDHs of Crenarchaea. Bacterial G1PDHs appeared as a paraphyletic group, since* T. pendens* and* Bacillus subtilis* G1PDHs formed a group that was a sister group of other bacterial G1PDHs. The group consisting of* T. pendens* and bacterial G1PDHs is also a subgroup of Crenarchaea G1PDHs. In summary, bacterial G1PDH is a subgroup of archaeal G1PDH. Therefore, this analysis also supports the hypothesis that* C. archaea* acquired G1PDH.

### 3.2. Phylogenetic Analysis of G3PDH (GpsA)

**Figure 4:**
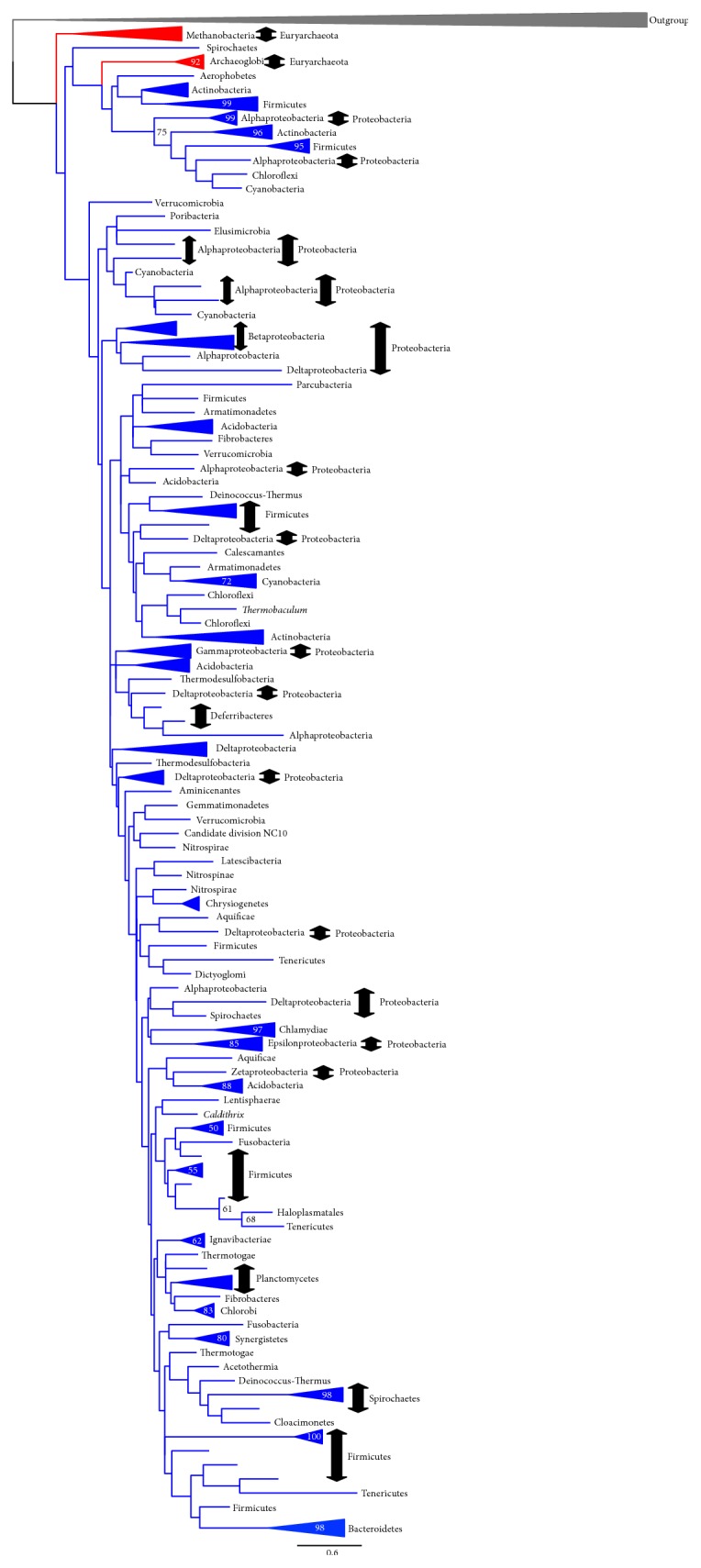
Outline of the G3PDH (GpsA) tree (ML method). The tree was constructed using RAxML version 7.4.2 [29] with the PROTGAMMALG model. The alignment with 305 OTUs and with 84 sites without any indels was used. The bootstrap analysis was carried out with 100 resamplings (slow option). The log likelihood of this tree is −29616.6. The bootstrap probability (BP) larger than 50% is shown at each node of the tree. The monophyletic group consisting of the same taxonomic group is shown in a simplified presentation. For the details of this tree, see Supplementary Figure S11.

The major G3PDH, synthesizing G3P from DHAP in Bacteria, is GpsA. We performed a molecular phylogenetic analysis of GpsA to evaluate its presence/absence in the* C. commonote*,* C. archaea*, and* C. bacteria*. An outline of the ML tree of GpsA is shown in Figure 4 (details of this tree can be seen in Supplementary Figure S11). A limited number of archaeal GpsAs, consisting of only Archaeoglobi and Methanobacteria, were included in our molecular phylogenetic analysis of GpsA, because only a few archaeal groups harbor the GpsAs. The archaeal GpsAs do not form a monophyletic group in this tree, and they are branched relatively close to the basal position of the GpsA sequences. Relationships among archaeal and bacterial GpsA could not be resolved in the BI analysis of GpsA (Supplementary Figure S12). It is likely that the* C. archaea *did not have GpsA and that certain archaeal lineages later acquired GpsA from bacterial species via horizontal gene transfer. However, we cannot ignore the idea that the* C. archaea* and also* C. commonote* carried GpsA.

### 3.3. Phylogenetic Analysis of G3PDH (GlpA/GlpD)

**Figure 5:**
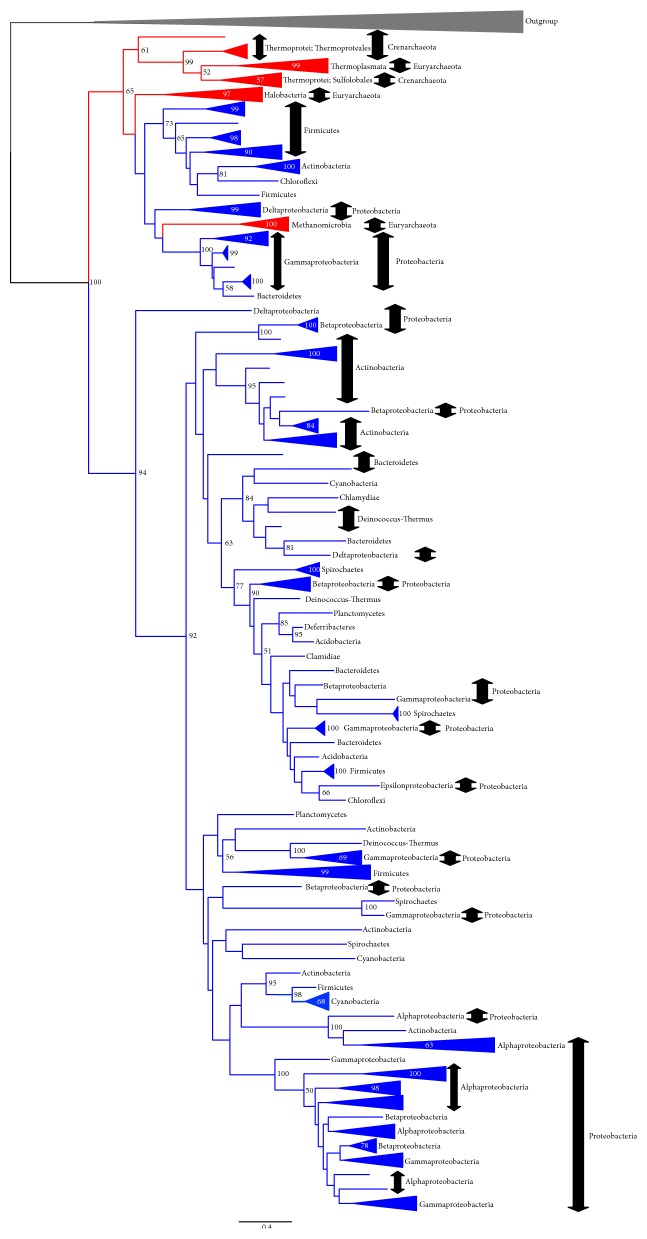
Outline of the G3PDH (GlpA/GlpD) tree (ML method). The tree was constructed using RAxML version 7.4.2 [29] with the PROTGAMMALG model. The alignment with 282 OTUs and with 239 sites without any indels was used. The bootstrap analysis was done with 100 resamplings (slow option). The log likelihood of this tree is −70287.6. The bootstrap probability (BP) larger than 50% is shown at each node of the tree. The monophyletic group consisting of the same taxonomic group is shown in a simplified presentation. For the details of this tree, see Supplementary Figure S13.

We used 282 species sequences for phylogenetic analyses. In the ML tree, GlpA/GlpD is divided into two groups. One is formed by archaeal and bacterial sequences and the other by only bacterial sequences (Figure 5; a detailed version of this tree is found in Supplementary Figure S13). Because all of the archaeal sequences were included in one of the two GlpA/GlpD groups in this tree and because most of the archaeal sequences in this group appeared as basal groups, we interpret this to mean that the* C. commonote*,* C. bacteria*, and* C. archaea* had GlpA/GlpD. The bacterial sequences in the archaeal branch in Figure 5 might have been horizontally transferred from archaeal species to bacterial species. Note that GlpA representing anaerobic G3PDH and GlpD for aerobic G3PDH were not resolved as separate groups in our ML tree, as was reported in preceding studies, for example, Peretó et al. [7].

In the BI tree, the relationships among six monophyletic groups [two archaeal groups (A1 and A2 in Supplementary Figure S14) and four bacterial groups (B1–B4 in Supplementary Figure S14)] were not clear. GlpA/GlpD appeared to have evolved in a polytomous manner in this tree. The largest bacterial group, GlpA/GlpD (B4), corresponds to the bacterial group in the GlpA/GlpD ML tree (Figure 5). The remaining five groups (A1, A2, and B1–B3) in Supplementary Figure S14 form a group in the ML tree presented in Figure 5. This also suggests that the archaeal GlpA/GlpD has a deep origin and that the* C. archaea* had GlpA/GlpD.

### 3.4. Phylogenetic Analysis of GlpK

**Figure 6:**
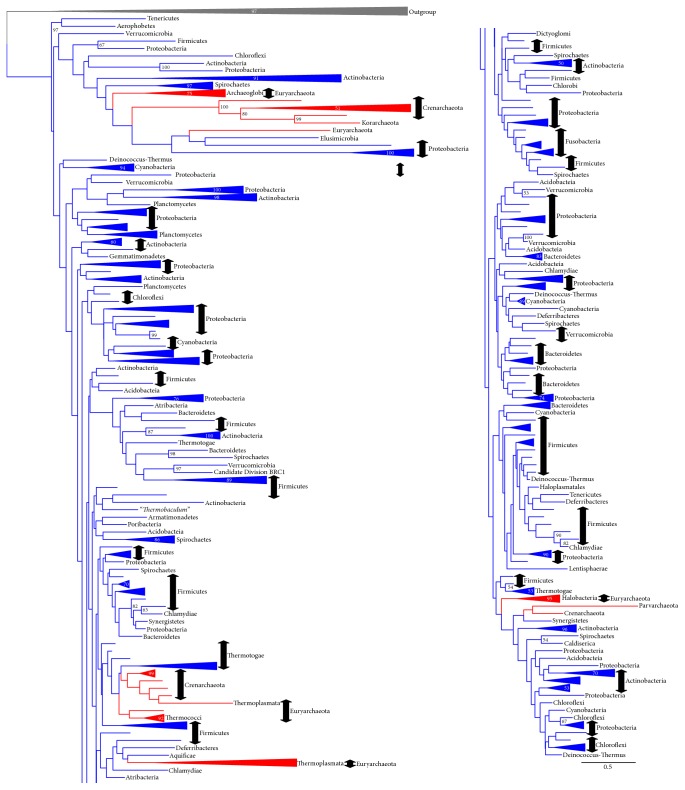
Outline of the GK (GlpK) tree (ML method). The tree was constructed using RAxML version 7.4.2 [29] with the PROTGAMMALG model. The alignment with 374 OTUs and with 194 sites without any indels was used. The bootstrap analysis was done with 100 resamplings (slow option). The log likelihood of this tree is −79246.3. The bootstrap probability (BP) larger than 50% is shown at each node of the tree. The monophyletic group consisting of the same taxonomic group is shown in a simplified presentation. For the details of this tree, see Supplementary Figure S15.

In Figure 6, the outline of the ML tree of GlpK is shown (a detailed version of this tree is found in Supplementary Figure S15). In this tree, archaeal GlpKs appeared as polyphyletic groups. It is most likely that the archaea acquired GlpKs via horizontal gene transfer from Bacteria.

### 3.5. Location of G1PDH for G1P Synthesis in Archaeal and Bacterial Groups

To discuss the evolution of chirality of polar lipids in cellular membranes, we listed archaeal and bacterial groups carrying G1PDH gene (*egsA*/*araM*) in Table 1.

In Archaea, G1PDH (EgsA) is found in phylum Euryarchaeota and TACK superphylum except for Lokiarchaeota (Table 1). However, the DPANN superphylum did not carry G1PDH (EgsA) among the archaeal groups listed in Table 1. In Bacteria, G1PDH (EgsA/AraM) is found in only a limited number of bacterial groups. The EgsA (or AraM) is found in only 14 of 44 bacterial phyla listed in Table 1.

### 3.6. Distribution of G3PDH and GK for G3P Synthesis in Archaeal and Bacterial Groups

We also listed archaeal and bacterial groups that carry the G3PDH gene (*gpsA* and* glpA*/*glpD*) and the GK gene (*glpK*) in Table 1. The* gpsA* gene is found in almost all of the bacterial groups listed in Table 1, with only a few exceptions (Atribacteria and Caldiserica). Complete genome sequences are not available for Atribacteria [43, 44]. Therefore, Atribacteria may have the* gpsA* gene. On the other hand, no* gpsA* gene was identified in the complete sequence of* Caldisericum exile* AZM16x01 genome (NC_017096), which is shown as a circular genome, In Archaea, the GpsA was found in only three euryarchaeal groups, Archaeoglobi, Metanobacteria, and Methanomicrobia, in addition to Woesearchaeota of the DPANN superphylum.

The GlpA/GlpD is found in 25 of 44 bacterial phyla listed in Table 1. In Archaea, the GlpA/GlpD is also found in several groups (four classes of Euryarchaea, and two orders of Crenarchaea, Korarchaeota, and Lokiarchaeota).

GlpK, a glycerol kinase (GK), is found in 32 of 44 bacterial phyla listed in Table 1. Among 32 bacterial phyla having the* glpK* gene, 23 phyla have* glpA*/*glpD* genes. Among Archaea, four classes of Euryarchaea, three Crenarchaea orders, Aigarchaeota, and Lokiarchaeota, have the* glpK* gene. Archaeoglobi and Halobacteria in Euryarchaeota, Sulfolobales and Thermoproteales in Crenarchaeota, and Lokiarchaeota have the* glpA*/*glpD* gene in addition to the* glpK* gene (Table 1).

### 3.7. When Did G1PDH Appear?

As shown in Figure 3 and Supplementary Figures S9 and S10, bacterial G1PDHs seem to have originated in Archaea. If this is true, neither the common ancestor of Bacteria nor the Commonote (LUCA) had G1PDH. Therefore, Archaea might have acquired G1PDH during a very early stage of evolution.

When did Archaea acquire G1PDH? Phylum Euryarchaeota and TACK superphylum except Lokiarchaeota have G1PDH (Table 1). On the other hand, members of the DPANN superphylum do not carry the* egsA*/*araM* gene (Table 1).

There are two possibilities regarding the phylogenetic position of the DPANN superphylum. One is that it is the basal group(s) of Archaea. In several phylogenetic studies, such as Rinke et al. [45] and Castelle et al. [38], it has been suggested that the DPANN superphylum is the basal group of Archaea. If the DPANN superphylum is the basal group of Archaea, then the common ancestor of all archaeal groups may not have needed a G1PDH gene. The common ancestor of the Euryarchaeota + TACK superphylum (Thaumarchaeota, Aigarchaeota, Crenarchaeota, Korarchaeota, and Lokiarchaeota) may have acquired G1PDH.

The other possibility is that groups (phyla) of DPANN superphylum are subgroups of either the Euryarchaeota or the TACK superphylum. Nanoarchaeota has been suggested to be a close relative of Thermococci in Euryarchaeota [46, 47]. Nanohaloarchaeota also has been suggested to be a close relative of Halobacteria in Euryarchaeota [47]. It has been suggested that Parvarchaeota (and Micrarchaeota) (ARMAN) are a subgroup of Euryarchaeota (relatives of Thermoplasmata) [48]. If* C. archaea* acquired G1PDH after dividing the DPANN superphylum lineage from the phylum Euryarchaeota and TACK superphylum, then the DPANN superphylum may have lost the G1PDH gene. If each group in the DPANN superphylum is a subgroup of the Euryarchaeota (and/or TACK superphylum) instead of the basal group,* C. archaea* may have acquired the G1PDH (gene).

Jahn et al. [49] reported that the nanoarchaeote* Nanoarchaeum equitans*, a parasite of crenarchaeote* Ignicoccus* sp. strain KIN4/I, uses a membrane lipid synthesized by* Ignicoccus* sp. (the host). Another nanoarchaeote Nst1 also has been suggested to be a parasite of Sulfolobales' cells [50]. Baker et al. [51] reported that parvarchaeote (AMANN) cells contact* Thermoplasma* cells, suggesting that a Parvarchaeote cell can obtain membrane lipids from a* Thermoplasma* cell. Not all members of the DPANN superphylum may be parasites of other archaeal cells. However, they are known to be nanoorganisms and to have small genomes. Even if they are not parasites, they may participate in tightly connected metabolic pathways formed by the ecological community [45]. This may allow a loss of the G1PDH gene from the genomes of members of the DPANN superphylum.

The alternative scenarios mentioned above do not change our most important conclusion regarding the G1PDH tree presented in Figure 3—that the* C. commonote* did not have G1PDH. As seen in our G1PDH trees (Figure 3; Supplementary Figures S9 and S10), bacterial G1PDHs show faster evolutionary rates than archaeal G1PDHs. Although bacterial G1PDHs are subgroups of archaeal G1PDHs in these trees, the statistical support for these divisions was not high (Figure 3; Supplementary Figures S9 and S10).

On the other hand, previous studies have suggested that the common ancestor of Bacteria had G1PDH; therefore the Commonote would have had G1PDH [7, 25, 26]. In the analyses carried out by Daiyasu et al. [25] and Carbone et al. [26], they used only the Neighbor Joining method. Daiyasu et al. [25] used the Maximum Likelihood estimation of pairwise distances under the JTT model without considering the different evolutionary rates among the sites. In Carbone et al. [26], details of the pairwise distances estimation are not presented (no models were described in their paper). Peretó et al. [7] performed Bayesian analyses under the JTT + Γ(8) model. Although the monophyly of G1PDH is supported by a relatively high posterior-probability (0.95) in their tree, it is difficult to conclude that bacterial G1PDHs and archaeal G1PDHs are separate monophyletic groups, since the posterior-probabilities supporting monophyly of bacterial G1PDHs and monophyly of archaeal G1PDHs are small and neglectable (0.50 and 0.65, resp.).

The different results between our study and preceding studies can be explained by the long branch attraction (LBA)—fast-evolving OTUs that tend to appear in phylogenetic positions near the root of tree, because of lower similarities between fast-evolving OTUs from other sequences [52, 53]. As can be seen in Figure 3, bacterial F1PDH, especially of Firmicutes, have long branches. We think that LBA caused the G1PDH tree topologies of Daiyasu et al. [25], Peretó et al. [7], and Carbone et al. [26], where archaeal G1PDHs are subgroups of bacterial G1PDHs [25, 26] or bacterial G1PDHs form a separate group from the archaeal G1PDHs. LBA may also explain the statistical support for bacterial G1PDHs being subgroups of archaeal G1PDHs in our G1PDH trees. The CAT model used in our BI analyses has often been suggested to produce a more robust tree than other evolutionary substitution models when variation of evolutionary rates among OTUs is high [53]. Therefore, our hypothesis (that G1PDH was acquired by the archaeal ancestor) seems more likely than the hypotheses proposed by Daiyasu et al. [25], Peretó et al. [7], and Carbone et al. [26] suggesting that G1PDH existed in the* C. commonote*.

### 3.8. Bacterial Common Ancestor Had a G3P Polar Lipid Membrane

Our phylogenetic analysis of GpsA indicates that it is the major G3PDH in Bacteria, forming G3P from DHAP (Figure 4; Supplementary Figures S11 and S12). A survey of the distribution of GpsA among bacterial lineages (Table 1) suggested that the bacterial common ancestor had GpsA, as suggested in Peretó et al. (2004). Only two groups (Atribacteria and Caldiserica) seem to lack this gene. These groups are not basal groups of Bacteria [43, 54]. Therefore, the* C. bacteria* had GpsA as their G3PDH. In addition, our phylogenetic analyses suggested that* C. bacteria* had additional G3PDH, GlpA/GlpD, and glycerol kinase GlpK (Figures 5 and 6, Supplementary Figures S13–S16). These enzymes can also catalyze G3P formation (Figure 1). These proteins could also have contributed to G3P formation in the* C. bacteria*.

### 3.9. *C. commonote* Had G3P as the Polar Lipid of Its Cellular Membrane: Proposed Scenario of Early Cell Membrane Evolution

**Figure 7:**
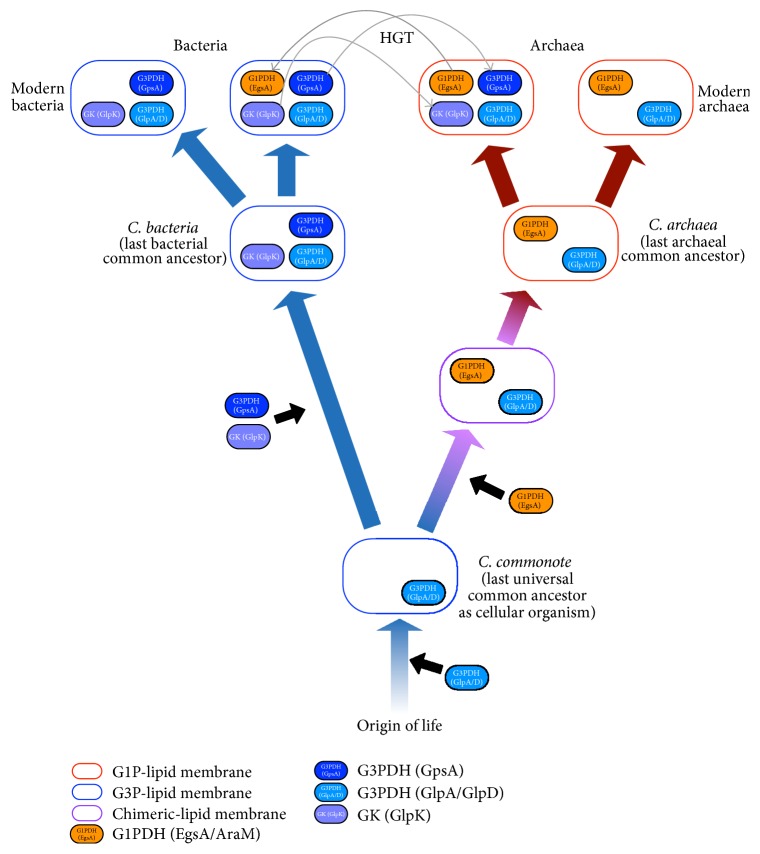
Proposed hypothesis based on the study presented in this paper. HGT: horizontal gene transfer.

Based on the above analyses, we propose a possible scenario describing the evolution of polar lipid chirality in cellular membranes (Figure 7). We infer that the* C. commonote* could form G3P by catalysis of GlpA/GlpD (G3PDH) (Figure 7). We do not have any direct evidence that the* C. commonote* could have synthesized a G1P polar lipid via enzymatic reactions. Therefore, it is most likely that the* C. commonote* had a cellular membrane with a G3P polar lipid, rather than a heterochiral polar lipid. Note that prior to acquiring GlpA/GlpD the ancestor of* C. commonote* might have had a heterochiral polar lipid membrane. G3P and G1P could have been synthesized by certain enzymatic activities [7, 9] or by nonenzymatic activities [55], but we do not know which enzyme or which chemical reaction contributed to the formation of G3P and G1P during that time period. As mentioned above, we do not have any direct evidence regarding the structure of cellular membranes in that era.

The quite early stage of the bacterial lineage had only GlpA/GlpD. Its descendant then acquired GpsA, the major G3PDH of modern bacterial species, as well as GlpK (GK forming G3P from glycerol), in addition to GlpA/GlpD (Figure 7). GpsA and GlpK were acquired prior to the appearance of* C. bacteria*. Thus,* C. bacteria* had a G3P-lipid membrane. After* C. bacteria *acquired GpsA, GpsA became the major enzyme responsible for synthesizing G3P in the cellular membrane.

The quite early stage of the archaeal lineage had GlpA/GlpD (G3PDH), so that the archaeal ancestor at this stage, before* C. archaea,* could have had a G3P polar lipid membrane rather than a G1P polar lipid membrane.* C. archaea* next acquired G1PDH (EgsA) in addition to a GlpA/GlpD homolog (G3PDH). In this stage, the archaeal ancestor could have had a heterochiral polar lipid membrane. Shimada and Yamagishi [56] suggested that the heterochiral polar lipid membrane is not less stable than homochiral polar lipid membranes. In addition, both G1PDH (EgsA) and G3PDH (GpsA, GlpA/GlpD) use DHAP as the substrate to form G1P and G3P, respectively (Figure 1), so that the G1PDH substrate already existed when this enzyme appeared in the archaeal lineage. In this sense, there should have been a “preadaptation” state to utilize G1PDH in the very early archaeal lineage before* C. archaea*. Thus, the change in polar lipid chirality from homochiral (G3P) to heterochiral might not have caused detrimental effects at this stage of the archaeal ancestor. After the stage of heterochiral polar lipid membranes, in the early evolution of the archaeal lineage, the heterochiral lipid membrane evolved to the G1P-homochiral lipid membrane.

An ether bond is generally more stable against hydrolysis than an ester bond, suggesting that membrane lipids with ether bonds are more stable than those with ester bonds in extreme environments such as high temperature and low/high pH. In addition, caldarchaeol is a membrane-spanning lipid found in hyperthermophilic archaeal species. When hyperthermophilic archaea is grown at higher temperatures, the portion of caldarchaeol is larger, suggesting that caldarchaeol is adaptive to high temperatures [57]. If the use of G1P had been tightly connected to the use of an ether lipid and (cald)archaeol, the change from heterochiral to G1P polar lipids in cellular membranes could have been adaptive to the hyperthermophilic archaeal ancestor, as suggested by Akanuma et al. [58, 59].

In certain bacterial lineages, G1PDH (EgsA) was acquired as AraM via horizontal gene transfer. In contrast, in certain archaeal lineages G3PDH (GpsA) was also acquired by horizontal gene transfer.

Although gene structures such as operon organization of genes and dispersed genes in the chromosomes would provide further evidences to the directionality of gene evolution and/or horizontal transfer, we could not find any characteristics of gene structures that made it possible for us to suggest the directionality of gene evolution and/or horizontal transfer.

### 3.10. Further Discussion: Origin of Eukaryotic Cellular Membranes

We did not discuss the origin of eukaryotic cellular membranes in this paper in order to focus on early stages of cellular membrane evolution from the age of* C. commonote* to the age in which Archaea and Bacteria were established, since we think that the appearance of Eukarya was a much later event than the appearance of Archaea and Bacteria. Recent studies have suggested an archaeal origin of Eukarya [2, 3, 59], although Bacteria are thought to contribute to the origin of Eukarya as, at least, the origin of the important organelles mitochondria and plastids. We will discuss this issue briefly.

No G1PDH (EgsA/AraM) has been reported from eukaryotic cells. On the other hand, the GpsA homolog (G3PDH) is known to be a major enzyme forming G3P in eukaryotic cells. GlpA/GlpD homologs have been found in various eukaryotic cells. However, their major reported roles are not the formation of G3P in cellular membranes; rather they carry out the “glycerol shuttle” and so on. In conclusion, from a quite early stage of eukaryal cell evolution, the eukaryotic cell membrane was a G3P-lipid membrane, not a G1P-lipid membrane.

Thus, the transition of membrane polar lipids from the G1P polar lipid to the G3P polar lipid occurred in an early step of eukaryote evolution. The GpsA homolog (Gpd) is likely to have a bacterial origin [7], probably via the mitochondrion or via horizontal gene transfer. As in the transition from a G3P polar lipid to a G1P polar lipid in the membrane of* C. archaea*, the transition from a G1P polar lipid to a G3P polar lipid in the membrane of the eukaryotic common ancestor could have been a neutral process, but it was not a disadvantageous process, since heterochiral membranes are as stable as homochiral membranes [56].

Interestingly, Lokiarchaeota seems to carry no G1PDH (EgsA), as mentioned above, but they do have G3PDH (GlpA/GlpD) (Table 1). Lokiarchaeota also seems to carry no G3PDH (GpsA) (Table 1). The lokiarchaeal genome was “reconstructed” via environmental DNA sequencing [3, 4]. Therefore, the absence of lokiarchaeal EgsA could be attributed to the complicated process of sequence determination and/or the complex structure of the lokiarchaeal genome, whereas Lokiarchaeota might not have EgsA, analogous to members of the DPANN superphylum (Table 1). Lokiarchaeota is known only from environmental DNA data, and no lokiarchaeal species have been isolated. Therefore, the nature of the lokiarchaeal membrane lipid is not known. Does Lokiarchaeota have a G1P-lipid or a G3P-lipid? This is quite an interesting question regarding the origin of the eukaryal cell, since Lokiarchaeota was proposed to be the most closely related to Eukarya among the archaeal group [3, 4].

## 4. Conclusion

In this paper, we propose a hypothesis regarding the early evolution of chirality of polar membrane lipids based on molecular phylogenetic analyses of enzymes determining the chirality of polar lipids in cellular membranes. By considering molecular phylogenetic analyses of enzymes contributing to fatty acid biosynthesis and isoprenoid biosynthesis and by connecting G3P/G1P and long hydrocarbonate chains (fatty acids/isoprenoids) with molecular phylogenetic analyses presented in this paper, the detailed history of cellular membrane evolution will become clearer.

## Supplementary Material

Supplementary Table S1: The list of sequence entries used to infer the G1PDH (EgsA/AraM) tree.
Supplementary Table S2: The list of sequence entries used to infer the G3PDH (GpsA) tree.
Supplementary Table S3: The list of sequence entries used to infer the G3PDH (GlpA/D) tree.
Supplementary Table S4: The list of sequence entries used to infer the GK (GlpK) tree.
Supplementary Table S5: Statistical test showing a maximum likelihood analysis of G1PDH. The AU test [34] was performed using Consel v0.1j [35] to test various alternative phylogenetic hypotheses. Based on the ML tree of G1PDH inferred by the RAxML, we divided G1PDHs into 8 groups, *Thermofilum pendens* Hrk-5 (Thermoproteales of Crenarchaeota) (A), Most Thermoproteales (rest of Thermoproteales) (B), Desulfurococcales + Acidilobales + Sulfolobales (C), Thaumarchaeota (D), Euryarchaeota (E), *Bacillus subtilis* subsp. *subtilis* str. 168 (F), Deltaproteobacteria + Haloplasmatales + *Anoxybacillus flavithermus* WK1 + *Bacillus cellulosilyticus* DSM 2522 (G), and Gammaproteobacteria + Actinobacteria (H), together with outgroup (O). Under the two constraint conditions ({{A, F, G, H}, B, C, D, E, O} and {A, B, C, D, E, {F, G, H, O}}), we listed 3,150 relationships among 8 G1PDH groups and 1 outgroup, using ProtML of Molphy 3.2b [36]. Next, the 3,150 relationships were used as the constraint for an ML tree search performed with RAxML with the PROTGAMMALG model. The log-likelihoods of 3,150 resultant trees were compared, and the top 2,000 trees on the log-likelihoods were then used for the AU test with Consel. The species (or groups) with white columns form a group together with the outgroup. Those with red columns form a distinct subgroup within the group including the outgroup (white columns).
Supplementary Figure S1: The trimed multiple alignment used for the phylogenetic analyses of G1PDH (EgsA/AraM). Details how to create this alignment is found in section 2.1 of main text.
Supplementary Figure S2. Alignment of G1PDH (EgsA/AraM) with selected sequences. The alignment is the subset of alignment used for the phylogenetic analysis. Sto: *Sulfolobus tokodaii* str. 7, DDBJ/GenBank/EMBL accession No. P58460. Afu: *Archaeoglobus fulgidus* DSM 4304, NP_070502. Msm: *Methanobrevibacter smithii* DSM 2374, ZP_05976397. Sco: *Streptomyces oelicoflavus* ZG0656, EHN78548. Bsu: *Bacillus subtilis* subsp. *subtilis* str. 168, NP_390754.
Supplementary Figure S3: The trimed multiple alignment used for the phylogenetic analyses of G3PDH (GpsA). Details how to create this alignment is found in section 2.2 of main text.
Supplementary Figure S4: Alignment of G3PDH (GpsA) with selected sequences. The alignment is the subset of alignment used for the phylogenetic analysis. Asu: *Archaeoglobus sulfaticuallidus*, DDBJ/GenBank/EMBL accession No. WP_015591014. Mru: *Methanobrevibacter ruminantium*. WP_012956986, Bsu: *Bacillus subtilis* subsp. *subtilis* str. 168, AAA86746. Eco: *Escherichia coli* str. K-12 substr. MG1665, AAB18585. Tth: *Thermus thermophilus* HB8, Q5SHJ0.
Supplementary Figure S5: The trimed multiple alignment used for the phylogenetic analyses of G3PDH (GlpA/GlpD). Details how to create this alignment is found in section 2.3 of main text.
Supplementary Figure S6: Alignment of G3PDH (GlpA/GlpD) with selected sequences. The alignment is the subset of alignment used for the phylogenetic analysis. Sso: *Sulfolobus solfataricus* P2, DDBJ/GenBank/EMBL accession No. NP_343866. Afu: *Archaeoglobus fulgidus* DSM 4304, NP_070157. Mar: *Methanocella arvoryzae* MRE50. YP_687586. Eco_GlpA: *Escherichia coli* str. K-12, P0A9C0 (GlpA). Eco_GlpD: *Escherichia coli*, 2R4J_A (GlpD). Tth: *Thermus thermophilus* HB8, YP_145382. Bsu: *Bacillus subtilis* subsp. *subtilis* str. 168, ZP_03590616.
Supplementary Figure S7: The trimed multiple alignment used for the phylogenetic analyses of GK (GlpK). Details how to create this alignment is found in section 2.4 of main text.
Supplementary Figure S8: Alignment of glycerol kinase (GlpK) with selected sequences. The alignment is the subset of alignment used for the phylogenetic analysis. Afu: *Archaeoglobus fulgidus* DSM 4304, DDBJ/GenBank/EMBL accession No. AAB90370. Sac: *Sulfolobus acidocaldarius* DSM 639, AAY80469. Bsu: *Bacillus subtilis* subsp. *subtilis* str. 168, AIY92216. Eco: *Escherichia coli* str. K-12, AAB03058. Tth: *Thermus thermophilus*, BAA28283.
Supplementary Figure S9: Detailed version of the G1PDH (EgsA/AraM) tree (ML method). This tree is a detailed version of the tree presented in Figure 3 in the main text. See detailed descriptions in the legend of Figure 3. At each node, the supporting bootstrap probability (BP) is shown (%).
Supplementary Figure S10: Detailed version of G1PDH (EgsA/AraM) the tree (BI method). The tree was constructed using PhyloBayes version 3.2f [32] under the CAT-Poisson (C20) + G (4) model. The alignment with 182 OTUs and with 252 sites without any indels was used. 200,000 MCMC cycles were performed, the sampling rate was every 10 cycles, and the first 50,000 cycles (5,000 sampled cycles) were discarded for further analysis. The log marginal likelihood of this tree is -45503.8±17.3. The Posterior probability (PP) is shown at each node of the tree.
Supplementary Figure S11: Detailed version of the G3PDH (GpsA) tree (ML method). This tree is a detailed version of the tree presented in Figure 3 in the main text. See detailed descriptions in the legend of Figure 3. At each node, the supporting BP is shown (%).
Supplementary Figure S12: Detailed version of the G3PDH (GpsA) tree (BI method). The tree was constructed using PhyloBayes version 3.2f [32] under the CAT-Poisson (C20) + G (4) model. The alignment with 305 OTUs and with 84 sites without any indels was used. 200,000 MCMC cycles were performed, the sampling rate was every 100 cycles, and the first 40,000 cycles (400 sampled cycles) were discarded for further analysis. The log marginal likelihood of this tree is -29860.5±27.3. The PP is shown at each node of the tree.
Supplementary Figure S13: Detailed version of the G3PDH (GlpA/GlpD) tree (ML method). This tree is a detailed version of the tree presented in Figure 3 in the main text. See detailed descriptions in the legend of Figure 3. At each node, the supporting BP is shown (%).
Supplementary Figure S14: Detailed version of the G3PDH (GlpA/GlpD) tree (BI method). The tree was constructed with PhyloBayes version 3.2f [32] under the CAT-Poisson (C20) + G (4) model. The alignment with 282 OTUs and with 239 sites without any indels was used. 200,000 MCMC cycles were performed, the sampling rate was every 100 cycles, and the first 40,000 cycles (400 sampled cycles) were discarded for further analysis. The log marginal likelihood of this tree is -70373.3±26.0. The PP is shown at each node of the tree. Six monophyletic subgroups of GlpA/GlpD are noted as A1 and A2 for archaeal groups and B1 to B4 for bacterial groups.
Supplementary Figure S15: Detailed version of the GK (GlpK) tree (ML method). This tree is a detailed version of the tree presented in Figure 3 in the main text. See detailed descriptions in the legend of Figure 3. At each node, the supporting BP is shown (%).
Supplementary Figure S16: Detailed version of the GK (GlpK) tree (BI method). The tree was constructed with PhyloBayes version 3.2f [32] under the CAT-Poisson (C20) + G (4) model. The alignment with 374 OTUs and with 194 sites without any indels was used. 200,000 MCMC cycles were performed, the sampling rate was every 100 cycles, and the first 50,000 cycles (500 sampled cycles) were discarded for further analysis. The log marginal likelihood of this tree is -77268.3±29.8. The PP is shown at each node of the tree.

## Figures and Tables

**Table 1:** Distribution of genes of G1PDH, G3PDH, and GK among archaea and bacteria.

Domain	Superphylum	Phylum	Class (order for Crenarchaeota)	G1PDH	G3PDH		GK
				EgsA/AraM	GpsA	GlpA/GlpD	GlpK
Archaea	DPANN	Diapherotrites					
		Parvarchaeota					**y**
		Micrarchaeota					
		Woesearchaeota			**y**		
		Pacearchaeota					
		Aenigmarchaeota					
		Nanoarchaeota					
		Nanohaloarchaeota					

Archaea		Euryarchaeota	Archaeoglobi	**Y**	**Y**	**Y**	**Y**
			Halobacteria	**Y**		**Y**	**Y**
			Methanobacteria	**Y**	**Y**		
			Methanococci	**Y**			
			Methanomicrobia	**Y**	**Y**	**Y**	
			Methanopyri	**Y**			
			Thermococci	**Y**		*y*	**Y**
			Thermoplasmata	**Y**		**Y**	**Y**

Archaea	TACK	Crenarchaeota	Acidilobales	**Y**		*y*	
			Desulfurococcales	**Y**		*y*	**Y**
			Fervidicoccales	**Y**			
			Sulfolobales	**Y**		**Y**	**Y**
			Thermoproteales	**Y**		**Y**	**Y**
		Korarchaeota		**Y**		**y**	**Y**
		Thaumarchaeota^*∗*^		**Y**			
		Aigarchaeota		**y**			**y**
		Lokiarchaeota				**Y**	*y*

Bacteria		Acetothermia			**y**	**Y**	
		Acidobacteria			**Y**	**Y**	**Y**
		Actinobacteria		**Y**	**Y**	**Y**	**Y**
		Aerophobetes			**y**		**y**
		Aminicenantes			**y**		
		Aquificae			**Y**		**Y**
		Armatimonadetes			**Y**		**Y**
		Atribacteria^*∗*^		**y**		**y**	**y**
		Bacteroidetes		**Y**	**Y**	**Y**	**Y**
		“*Caldithrix*”			**y**	**y**	
		Caldiserica				*y*	**Y**
		Calescamantes^*∗*^		**y**	**y**		
		Candidate division BRC1^*∗*^			**y**		**y**
		Candidate division NC10			**Y**		
		Chlamydiae			**Y**	**Y**	**Y**
		Chlorobi			**Y**		**Y**
		Chloroflexi		**Y**	**Y**	**Y**	**Y**
		Chrysiogenetes		**Y**	**Y**		
		Cloacimonetes			**y**		
		Cyanobacteria			**Y**	**Y**	**Y**
		Deferribacteres			**Y**	**Y**	**Y**
		Deinococcus-Thermus			**Y**	**Y**	**Y**
		Dictyoglomi		**Y**	**Y**		**Y**
		Elusimicrobia			**Y**		**Y**
		Fibrobacteres		**y**	**Y**		
		Firmicutes		**Y**	**Y**	**Y**	**Y**
		Fusobacteria			**Y**	*y*	**Y**
		Gemmatimonadetes			**Y**	**Y**	**Y**
		Haloplasmatales		**y**	**y**		**y**
		Ignavibacteriae			**Y**	**Y**	
		Latescibacteria^*∗*^			**y**		
		Lentisphaerae			**y**	**y**	**y**
		Nitrospinae			**Y**		
		Parcubacteria			**y**	**y**	**y**
		Planctomycetes		**Y**	**Y**	**Y**	**Y**
		Poribacteria			**y**		**y**
		Proteobacteria	Alphaproteobacteria	**Y**	**Y**	**Y**	**Y**
			Betaproteobacteria	**Y**	**Y**	**Y**	**Y**
			Deltaproteobacteria	**Y**	**Y**	**Y**	**Y**
			Epsilonproteobacteria		**Y**	**Y**	**Y**
			Gammaproteobacteria	**Y**	**Y**	**Y**	**Y**
			Zetaproteobacteria		**y**		
		Spirochaetes		**Y**	**Y**	**Y**	**Y**
		Synergistetes			**Y**	*y*	**Y**
		Tenericutes			**Y**	*y*	**Y**
		“*Thermobaculum*”			**Y**	**Y**	**Y**
		Thermodesulfobacteria			**Y**		
		Thermotogae		**Y**	**Y**	*y*	**Y**
		Verrucomicrobia		**y**	**Y**	**Y**	**Y**

**y**: at least one protein sequence of interest was found by our BLAST search. *y*: at least one protein sequence of interest is listed in the taxonomy in genes of KEGG (release 76.0) Orthology (K00096 for EgsA/AraM, K00057 for GpsA, K00111 for GlpA/D, and K00864 for GlpK) [37]. **Y**: at least one protein sequence of interest is found by our BLAST search and listed in the taxonomy in genes of KEGG Orthology. When no culturable species are known, but some genome sequences are available, from the phylum, the phylum was marked with an asterisk (*∗*).

Taxonomy in this table is based on NCBI Taxonomy. Exceptions are as follows: Micrarchaeota is shown as a separate phylum of Parvarchaeota. Woesearchaeota and Pacearchaeota [38] are shown. For BLAST search targeting Woesearchaeota and Pacearchaeota, the sequences (archaeon GW20011_AR3, 4, 9, 11, 15–18, and 20) and the sequences (archaeon GW20011_AR1, 6, 13, and 19) were used as Woesearchaeota and Pacearchaota sequences. Aigarchaeota is shown as a separate phylum of Thaumarchaeota.
