# Supplementary material for: Birth of Archaeal Cells: Molecular Phylogenetic Analyses of G1P Dehydrogenase, G3P Dehydrogenases, and Glycerol Kinase Suggest Derived Features of Archaeal Membranes Having G1P Polar Lipids
Source: Archaea. 2016 Sep 28;2016:1802675. doi: 10.1155/2016/1802675 (PMC5059525; doi:10.1155/2016/1802675)
Supplement: Supplementary file 1 — Supplementary Table S1: The list of sequence entries used to infer the G1PDH (EgsA/AraM) tree. Supplementary Table S2: The list of sequence entries used to infer the G3PDH (GpsA) tree. Supplementary Table S3: The list of sequence entries used to infer the G3PDH (GlpA/D) tree. Supplementary Table S4: The list of sequence entries used to infer the GK (GlpK) tree. Supplementary Table S5: Statistical test showing a maximum likelihood analysis of G1PDH. The AU test [34] was performed using Consel v0.1j [35] to test various alternative phylogenetic hypotheses. Based on the ML tree of G1PDH inferred by the RAxML, we divided G1PDHs into 8 groups, Thermofilum pendens Hrk-5 (Thermoproteales of Crenarchaeota) (A), Most Thermoproteales (rest of Thermoproteales) (B), Desulfurococcales + Acidilobales + Sulfolobales (C), Thaumarchaeota (D), Euryarchaeota (E), Bacillus subtilis subsp. subtilis str. 168 (F), Deltaproteobacteria + Haloplasmatales + Anoxybacillus flavithermus WK1 + Bacillus cellulosilyticus DSM 2522 (G), and Gammaproteobacteria + Actinobacteria (H), together with outgroup (O). Under the two constraint conditions ({{A, F, G, H}, B, C, D, E, O} and {A, B, C, D, E, {F, G, H, O}}), we listed 3,150 relationships among 8 G1PDH groups and 1 outgroup, using ProtML of Molphy 3.2b [36]. Next, the 3,150 relationships were used as the constraint for an ML tree search performed with RAxML with the PROTGAMMALG model. The log-likelihoods of 3,150 resultant trees were compared, and the top 2,000 trees on the log-likelihoods were then used for the AU test with Consel. The species (or groups) with white columns form a group together with the outgroup. Those with red columns form a distinct subgroup within the group including the outgroup (white columns). Supplementary Figure S1: The trimed multiple alignment used for the phylogenetic analyses of G1PDH (EgsA/AraM). Details how to create this alignment is found in section 2.1 of main text. Supplementary Figure S2. Alignment of G1PDH (Egs [file 1802675.f1.zip › Supplementary_Materials_Yokobori_et_al_part_1_ARCH_1737172.pdf]

Supplementary Materials

When Archaea appeared —Molecular phylogenetic analyses of G1P dehydrogenase, G3P dehydrogenases, and glycerol kinase suggest derived features of archaeal membranes having G1P-polar lipids

Shin-ichi Yokobori,[1] Yoshiki Nakajima,[1] Satoshi Akanuma,[2] & Akihiko Yamagishi[1]

[1]Laboratory of Extremophiles, Department of Applied Life Sciences, School of Life Sciences, Tokyo University of Pharmacy and Life Sciences, 1432-1 Horinouchi, Hachioji, Tokyo 192-0392, Japan.
[2]Faculty of Human Sciences, Waseda University, 2-579-15 Mikajima, Tokorozawa, Saitama 359-1192, Japan.

Correspondence should be addressed to Akihiko Yamagishi: yamagish@toyaku.ac.jp

Supplementary Table S1. The list of sequence entries used to infer the G1PDH (EgsA/AraM) tree.

Taxonomy	Organism	Accession
Archaea; Crenarchaeota; Thermoprotei; Acidilobales;	*Acidilobus saccharovorans* 345-15	YP_003815753
Archaea; Crenarchaeota; Thermoprotei; Desulfurococcales;	*Aeropyrum pernix* K1	NP_147296
	Desulfurococcus kamchatkensis 1221n	YP_002428982
	Ignicoccus hospitalis KIN4/I	YP_001435758
	Ignisphaera aggregans DSM 17230	YP_003858777
	Staphylothermus marinus F1	YP_001040946
	Staphylothermus hellenicus DSM 12710	YP_003669513
	Hyperthermus butylicus DSM 5456	YP_001013732
	Pyrolobus fumarii 1A	YP_004780337
Archaea; Crenarchaeota; Thermoprotei; Sulfolobales;	*Acidianus hospitalis* W1	YP_004457824
	Metallosphaera sedula DSM 5348	YP_001192317
	Metallosphaera cuprina Ar-4	YP_004410588
	Metallosphaera yellowstonensis MK1	ZP_09705471
	Sulfolobus tokodaii str. 7	P58460
	Sulfolobus solfataricus P2	NP_342265
	Sulfolobus islandicus L.S.2.15	YP_002832130
Archaea; Crenarchaeota; Thermoprotei; Thermoproteales;	*Thermofilum pendens* Hrk 5	YP_920632
	Caldivirga maquilingensis IC-167	A8MC03
	Pyrobaculum aerophilum str. IM2	NP_559473
	Thermoproteus uzoniensis 768-20	YP_004338788
	Thermoproteus tenax Kra 1	YP_004893223
	Vulcanisaeta moutnovskia 768-28	YP_004244434
Archaea; Euryarchaeota;	*Aciduliprofundum boonei* T469	YP_003482909
Archaea; Euryarchaeota; Archaeoglobi;	*Archaeoglobus fulgidus* DSM 4304	NP_070502
	Archaeoglobus veneficus SNP6	YP_004341260
	Ferroglobus placidus DSM 10642	YP_003436585
Archaea; Euryarchaeota; Halobacteria;	*Haladaptatus paucihalophilus* DX253	ZP_08044423
	Halalkalicoccus jeotgali B3	YP_003737916
	Haloarcula hispanica ATCC 33960	YP_004795531
	Halobacterium sp. NRC-1	NP_279479
	Halobacterium sp. DL1	ZP_09029887
	Haloferax volcanii DS2	YP_003534882
	Halogeometricum borinquense DSM 11551	YP_004037399
	Halomicrobium mukohataei DSM 12286	YP_003178951
	Halopiger xanaduensis SH-6	YP_004598244
	Haloquadratum walsbyi DSM 16790	YP_657330
	Halorhabdus tiamatea SARL4B	ZP_08559034
	Halorubrum lacusprofundi ATCC 49239	YP_002565327
	Haloterrigena turkmenica DSM 5511	YP_003404729
	Natrialba magadii ATCC 43099	YP_003480657
	Natrinema pellirubrum DSM 15624	ZP_08963262
	Natronobacterium gregoryi SP2	ZP_08968707
	Natronomonas pharaonis DSM 2160	YP_330974
Archaea; Euryarchaeota; Methanobacteria;	*Methanobacterium paludis*	YP_004520039
	Methanobrevibacter ruminantium M1	YP_003423698
	Methanobrevibacter smithii DSM 2374	ZP_05976397
	Methanosphaera stadtmanae DSM 3091	YP_448028
	Methanothermobacter thermautotrophicus str. Delta H	P72010

	Methanothermobacter marburgensis str. Marburg	YP_003849898
	Methanothermus fervidus DSM 2088	YP_004003574
Archaea; Euryarchaeota; Methanococci;	*Methanocaldococcus jannaschii* DSM 2661	NP_247697
	Methanocaldococcus vulcanius M7	YP_003247891
	Methanocaldococcus sp. FS406-22	YP_003458869
	Methanotorris formicicus Mc-S-70	ZP_09708452
	Methanococcus maripaludis S2	NP_987345
	Methanococcus maripaludis C5	YP_001097980
	Methanococcus maripaludis C7	YP_001330439
Archaea; Euryarchaeota; Methanomicrobia;	*Methanocella conradii* HZ254	AFC98788
	Methanocella paludicola SANAE	YP_003355244
	Methanocorpusculum labreanum Z	YP_001030742
	Methanoculleus marisnigri JR1	YP_001047953
	Methanolacinia petrolearia DSM 11571	YP_003894977
	Methanoplanus limicola DSM 2279	ZP_09701990
	Methanolinea tarda NOBI-1	ZP_09042444
	Methanoregula boonei 6A8	YP_001405118
	Methanosphaerula palustris E1-9c	YP_002466023
	Methanospirillum hungatei JF-1	YP_502604
	Methanosaeta harundinacea 6Ac	AET64327
	Methanosaeta thermophila PT	YP_842657
	Methanosaeta concilii GP6	YP_004383617
	Methanococcoides burtonii DSM 6242	YP_565717
	Methanohalobium evestigatum Z-7303	YP_003726269
	Methanohalophilus mahii DSM 5219	YP_003542550
	Methanosarcina acetivorans C2A	NP_618561
	Methanosarcina mazei Go1	NP_632614
	Methanosarcina barkeri str. Fusaro	YP_303855
Archaea; Euryarchaeota; Methanopyri;	*Methanopyrus kandleri* AV19	NP_614513
Archaea; Euryarchaeota; Thermococci;	*Pyrococcus horikoshii* OT3	O59144
	Pyrococcus abyssi GE5	NP_126372
	Pyrococcus furiosus DSM 3638	NP_579111
	Thermococcus kodakarensis KOD1	YP_183202
	Thermococcus barophilus MP	YP_004071243
	Thermococcus sp. 4557	YP_004763636
	Thermococcus litoralis DSM 5473	ZP_09730767
Archaea; Euryarchaeota; Thermoplasmata;	*Ferroplasma acidarmanus* fer1	ZP_05571072
	Picrophilus torridus DSM 9790	YP_023628
	Thermoplasma volcanium GSS1	NP_111746
	Thermoplasma acidophilum DSM 1728	NP_394614
Archaea; Thaumarchaeota; Cenarchaeales;	*Cenarchaeum symbiosum* A	A0RXV2
Archaea; Thaumarchaeota; Nitrosopumilales;	*Candidatus* Nitrosoarchaeum limnia SFB1	ZP_08257665
	Candidatus Nitrosoarchaeum koreensis MY1	ZP_08668836
	Nitrosopumilus maritimus SCM1	YP_001583063
	Candidatus Nitrosopumilus salaria BD31	ZP_09605882
Bacteria; Actinobacteria; Actinobacteridae;	*Nocardia brasiliensis* ATCC 700358	ZP_09840922
	Frankia sp. EAN1pec	YP_001510212
	Stackebrandtia nassauensis DSM 44728	YP_003514403
	Actinoplanes sp. SE50/110	AEV89150
	Micromonospora aurantiaca ATCC 27029	YP_003839261
	Micromonospora sp. ATCC 39149	ZP_04606494
	Salinispora arenicola CNS-205	YP_001539736
	Thermobispora bispora DSM 43833	YP_003651341

	Streptomyces venezuelae ATCC 10712	CCA59727
	Streptomyces coelicoflavus ZG0656	EHN78548
	Streptomyces clavuligerus ATCC 27064	ZP_06774702
	Streptomyces viridochromogenes DSM 40736	ZP_07307866
	Streptomyces zinciresistens K42	ZP_08803212
	Streptomyces sp. W007	ZP_09403399
	Nocardiopsis dassonvillei subsp. *dassonvillei* DSM 43111	YP_003681769
	Thermomonospora curvata DSM 43183	YP_003301673
Bacteria; Chlorobi; Chlorobia;	*Chlorobaculum parvum* NCIB 8327	YP_001998671
	Chlorobium ferrooxidans DSM 13031	ZP_01385829
Bacteria; Chrysiogenetes; Chrysiogenales;	*Desulfurispirillum indicum* S5	YP_004111517
Bacteria; Cyanobacteria; Nostocales;	*Anabaena variabilis* ATCC 29413	YP_320537
	Cylindrospermopsis raciborskii CS-505	ZP_06307585
	Nodularia spumigena CCY9414	ZP_01631542
	Nostoc punctiforme PCC 73102	YP_001868372
	Raphidiopsis brookii D9	ZP_06306057
	'Nostoc azollae' 0708	YP_003722140
Bacteria; Cyanobacteria; Oscillatoriales;	*Arthrospira platensis* str. Paraca	ZP_06380832
Bacteria; Cyanobacteria; Oscillatoriophycideae;	*Acaryochloris marina* MBIC11017	YP_001517882
	Crocosphaera watsonii WH 8501	ZP_00515762
	Cyanothece sp. ATCC 51142	YP_001802726
	Cyanothece sp. PCC 7822	YP_003885570
	Microcystis aeruginosa NIES-843	YP_001657591
	Synechococcus elongatus PCC 7942	YP_400670
	Synechococcus sp. JA-2-3B'a(2-13)	YP_476598
	Synechococcus sp. CC9311	YP_729867
	Thermosynechococcus elongatus BP-1	NP_682841
	Arthrospira platensis NIES-39	BAI91054
	Arthrospira maxima CS-328	ZP_03274582
	Coleofasciculus chthonoplastes PCC 7420	ZP_05024185
	Lyngbya sp. PCC 8106	ZP_01620809
	Microcoleus vaginatus FGP-2	ZP_08491102
	Trichodesmium erythraeum IMS101	YP_720871
Bacteria; Cyanobacteria; Prochlorales;	*Prochlorococcus marinus* str. MIT 9515	YP_001011492
Bacteria; Deinococcus-Thermus; Deinococci;	*Truepera radiovictrix* DSM 17093	YP_003704889
Bacteria; Firmicutes; Bacilli;	*Anoxybacillus* flavithermus *WK1*	YP_002315903
	Bacillus subtilis subsp. *subtilis* str. 168	NP_390754
	Bacillus cellulosilyticus DSM 2522	YP_004095225
	Listeria monocytogenes FSL J1-208	EHY62723
	Enterococcus italicus DSM 15952	ZP_07895093
	Enterococcus casseliflavus ATCC 12755	ZP_08144556
	Leuconostoc gelidum KCTC 3527	ZP_08479472
	Leuconostoc citreum KM20	YP_001728665
Bacteria; Firmicutes; Clostridia;	*Clostridium* sp. DL-VIII	ZP_09206713
	Eubacterium rectale ATCC 33656	YP_002938300
	Clostridium lentocellum DSM 5427	YP_004309602
	Roseburia intestinalis L1-82	ZP_04742161
	Desulfotomaculum nigrificans DSM 574	ZP_08114177
	Acetivibrio cellulolyticus CD2	ZP_09462476
	Moorella thermoacetica ATCC 39073	YP_429338

Bacteria; Fusobacteria; Fusobacteriales;	*Fusobacterium ulcerans* ATCC 49185	ZP_07929431
Bacteria; Haloplasmatales; Haloplasmataceae;	*Haloplasma contractile* SSD-17B	ZP_08558101
Bacteria; Planctomycetes; Planctomycetia;	*Planctomyces limnophilus* DSM 3776	YP_003629610
Bacteria; Proteobacteria; Alphaproteobacteria;	*Bradyrhizobiaceae bacterium* SG-6C	ZP_08628955
	Methylobacterium nodulans ORS 2060	YP_002500804
	Methylocystis sp. ATCC 49242	ZP_08074163
	Sinorhizobium meliloti 1021	NP_386656
	Sinorhizobium medicae WSM419	YP_001328127
	Xanthobacter autotrophicus Py2	YP_001416616
	Paracoccus sp. TRP	ZP_08664313
	Roseomonas cervicalis ATCC 49957	ZP_06895512
	Acetobacteraceae bacterium AT-5844	ZP_09398437
Bacteria; Proteobacteria; Deltaproteobacteria;	*Desulfarculus baarsii* DSM 2075	YP_003806763
	Desulfatibacillum alkenivorans AK-01	YP_002430034
	Desulfovibrio vulgaris DP4	YP_961214
	Desulfovibrio aespoeensis Aspo-2	YP_004122072
	Desulfovibrio fructosovorans JJ	ZP_07334616
	Desulfovibrio sp. FW1012B	ZP_09131560
	Geobacter lovleyi SZ	YP_001950958
	Sorangium cellulosum So ce56	YP_001616037
	Syntrophobacter fumaroxidans MPOB	YP_844654
Bacteria; Proteobacteria; Gammaproteobacteria;	*Pseudoalteromonas tunicata* D2	ZP_01134701
	Shewanella violacea DSS12	YP_003554854
	Shewanella benthica KT99	ZP_02156139
	Escherichia coli str. K-12 substr. MG1655	AAC76927
	Pseudomonas putida W619	YP_001748564
Bacteria; Spirochaetes; Spirochaetales;	*Brachyspira intermedia* PWS/A	AEM22479
Bacteria; Thermotogae; Thermotogales;	*Kosmotoga olearia* TBF 19.5.1	YP_002940231
	Thermotoga maritima	1KQ3_A
	Thermotoga petrophila RKU-1	YP_001244096
	Thermotoga neapolitana DSM 4359	YP_002533789

Supplementary Table S2. The list of sequence entries used to infer the G3PDH (GpsA) tree.

Taxonomy	Organism	Accession
Archaea; Crenarchaeota; Thermoprotei; Acidilobales;	*Acidilobus saccharovorans*	WP_013266948
	Caldisphaera lagunensis	WP_015231820
Archaea; Crenarchaeota; Thermoprotei; Desulfurococcales;	*Aeropyrum pernix*	WP_010866401
	Pyrolobus fumarii	WP_014026963
Archaea; Crenarchaeota; Thermoprotei; Sulfolobales;	*Sulfolobus tokodaii*	WP_010978008
	Sulfolobus solfataricus	WP_010923104
Archaea; Crenarchaeota; Thermoprotei; Thermoproteales;	*Thermofilum pendens*	WP_011753379
	Thermoproteus tenax	WP_014126928
Archaea; Diapherotrites;	*Candidatus* Iainarchaeum andersonii	WP_041910265
Archaea; Euryarchaeota; Archaeoglobi;	*Archaeoglobus sulfaticallidus*	WP_015591014
	Archaeoglobus fulgidus DSM 8774	AIG96861
		AIG97998
	Archaeoglobus fulgidus	WP_010878100
	Ferroglobus placidus	WP_012965915
Archaea; Euryarchaeota; Halobacteria;	*Halobacterium salinarum*	WP_010902952
		WP_010901982
Archaea; Euryarchaeota; Methanobacteria;	*Methanobacterium formicicum* DSM 3637	EKF86026
	Methanobrevibacter ruminantium	WP_012956986
	Methanothermobacter thermautotrophicus CaT2	BAM69559
Archaea; Euryarchaeota; Methanococci;	*Methanocaldococcus jannaschii* DSM 2661	Q57871
Archaea; Euryarchaeota; Methanomicrobia;	*Methanocella paludicola* SANAE	BAI62488
	Methanoculleus marisnigri	WP_011844442
	Methanoculleus marisnigri JR1	ABN56632
	Methanosarcina mazei	WP_015411604
Archaea; Euryarchaeota; Thermococci;	*Pyrococcus furiosus*	WP_011012501
Archaea; Euryarchaeota; Thermoplasmata;	*Methanomassiliicoccus luminyensis*	WP_026068756
	Thermoplasma acidophilum	WP_010900720
Archaea; Korarchaeota;	*Candidatus* Korarchaeum cryptofilum	WP_012308771
Bacteria; Acetothermia;	*Candidatus* Acetothermus autotrophicum	BAL59589
Bacteria; Acidobacteria; Acidobacteriales;	*Acidobacterium capsulatum*	WP_015898440
		WP_015895922
	Candidatus Koribacter versatilis	WP_011522939
	Granulicella tundricola	WP_013579418
	Terriglobus roseus	WP_014784837
Bacteria; Acidobacteria; Acidobacteria subdivision 4;	*Candidatus* Chloracidobacterium thermophilum	WP_014100138
Bacteria; Acidobacteria; Acidobacteria subdivision 23;	*Thermoanaerobaculum aquaticum*	KDA54421
Bacteria; Acidobacteria; Holophagae;	*Geothrix fermentans*	WP_026853830
	Holophaga foetida	WP_005034894
Bacteria; Acidobacteria; Solibacteres;	*Candidatus* Solibacter usitatus	WP_011682101
		WP_011687232
		WP_041858319
	Candidatus Solibacter usitatus Ellin6076	ABJ87618
		ABJ88405
	Bryobacter aggregatus	WP_031500416
Bacteria; Actinobacteria; Actinobacteridae;	*Actinomyces turicensis* ACS-279-V-Col4	EJZ85434
		EJZ86488
		WP_022868963

	Actinomyces vaccimaxillae	WP_026459441
	Actinomyces slackii	WP_026427628
	Corynebacterium variabile	WP_014010264
		WP_030146704
	Corynebacterium efficiens	WP_006769599
	Microbacterium indicum	WP_029150047
	Propionibacterium propionicum	WP_014845387
		WP_014847302
	Streptomyces sviceus ATCC 29083	EDY53897
		EDY55566
Bacteria; Actinobacteria; Coriobacteridae;	*Eggerthella* sp. YY7918	BAK44800
Bacteria; Actinobacteria; Rubrobacteridae;	*Rubrobacter xylanophilus*	WP_011565017
	Rubrobacter radiotolerans	AHY48316
Bacteria; Aerophobetes	Aerophobetes bacterium SCGC AAA255-F10	WP_029962257
Bacteria; Aerophobetes;	*Candidatus* Aerophobus profundus	WP_041891889
Bacteria; Aminicenantes;	*Candidatus* Aminicenans sakinawicola	WP_020260390
Bacteria; Aquificae; Aquificales;	*Aquifex aeolicus* VF5	O67555
	Aquifex aeolicus	WP_010879940
Bacteria; Aquificae; Desulfurobacteriales;	*Desulfurobacterium thermolithotrophum*	WP_013638206
	Thermovibrio ammonificans HB-1	ADU96654
Bacteria; Armatimonadetes; Chthonomonadetes;	*Chthonomonas calidirosea* T49	CCW34705
Bacteria; Armatimonadetes;	*Fimbriimonas ginsengisoli* Gsoil 348	AIE83379
	Fimbriimonas ginsengisoli	WP_025229365
Bacteria; Atribacteria	Atribacteria bacterium SCGC AAA255-G05	WP_029717092
Bacteria; Bacteroidetes; Bacteroidia;	*Bacteroides fluxus* YIT 12057	EGF55486
	Draconibacterium orientale	AHW61232
Bacteria; Bacteroidetes; Cytophagia;	*Cyclobacterium marinum*	WP_014018215
		WP_014022044
	Cytophaga aurantiaca	WP_018342739
	Cytophaga hutchinsonii	WP_011586931
Bacteria; Bacteroidetes; Flavobacteriia;	*Fluviicola taffensis*	WP_013687203
		WP_013686815
	Flavobacterium psychrophilum FPG3	AIN73080
	Flavobacterium aquatile LMG 4008	KGD68544
Bacteria; Bacteroidetes; Sphingobacteriia;	*Chitinophaga pinensis*	WP_012794626
	Saprospira grandis	WP_015692095
	Sphingobacterium paucimobilis HER1398	ERJ58061
Bacteria; Caldiserica	Caldiserica bacterium JGI 0000059-M03	WP_041907939
Bacteria; Caldithrix	*Caldithrix abyssi* DSM 13497	EHO42813
	Caldithrix abyssi	WP_006930620
Bacteria; Calescamantes;	*Candidatus* Calescibacterium nevadense	WP_022804334
		WP_029665305
Bacteria; candidate division NC10;	*Candidatus* Methylomirabilis oxyfera	CBE68583
Bacteria; Chlamydiae; Chlamydiales;	*Chlamydia trachomatis* A/HAR-13	AAX50992
	Chlamydophila pneumoniae AR39	AAF38792
	Chlamydia sp. 'Rubis'	CDZ80591
	Parachlamydia acanthamoebae	KIA78740
	Simkania negevensis Z	CCB88100
	Waddlia chondrophila	WP_013182529

	Waddlia chondrophila WSU 86-1044	ADI39161
Bacteria; Chlorobi; Chlorobia;	*Chlorobium ferrooxidans* DSM 13031	EAT59593
	Chlorobium limicola	WP_012466345
	Prosthecochloris aestuarii	WP_012506707
Bacteria; Chloroflexi; Anaerolineae;	*Anaerolinea thermophila* UNI-1	BAJ64751
Bacteria; Chloroflexi; Caldilineae;	*Caldilinea aerophila* DSM 14535 = NBRC 104270	BAM00728
	Caldilinea aerophila	WP_014432352
Bacteria; Chloroflexi; Chloroflexia;	*Chloroflexus aggregans*	WP_012615659
		WP_012616384
	Herpetosiphon aurantiacus	WP_012191918
		WP_012191410
Bacteria; Chloroflexi; Dehalococcoidia	Dehalococcoidia bacterium SCGC AB-539-J10	WP_029475349
Bacteria; Chloroflexi; Dehalococcoidia;	*Dehalococcoides mccartyi* CBDB1	CAI83405
Bacteria; Chloroflexi; Ktedonobacteria;	*Ktedonobacter racemifer*	WP_007908052
Bacteria; Chloroflexi; Sphaerobacteridae;	*Sphaerobacter thermophilus*	WP_012873366
Bacteria; Chrysiogenetes; Chrysiogenales;	*Chrysiogenes arsenatis*	WP_027389158
		WP_027389174
	Desulfurispirillum indicum	WP_013506429
		WP_013506025
Bacteria; Cloacimonetes;	*Candidatus* Cloacimonas acidaminovorans str. Evry	CAO80747
Bacteria; Cloacimonetes;	*Candidatus* Cloacimonas acidaminovorans	WP_015425177
Bacteria; Cyanobacteria; Gloeobacteria;	*Gloeobacter kilaueensis* JS1	AGY57217
	Gloeobacter kilaueensis	WP_023173759
Bacteria; Cyanobacteria; Nostocales;	*Hassallia byssoidea* VB512170	KIF34618
	Scytonema hofmanni	WP_033336837
Bacteria; Cyanobacteria; Oscillatoriophycideae;	*Crocosphaera watsonii* WH 8501	EAM47652
	Microcystis aeruginosa PCC 7806	CAO88267
	Synechococcus elongatus PCC 7942	Q935Z2
	Arthrospira platensis NIES-39	BAI93051
	Nodosilinea nodulosa	WP_026073203
Bacteria; Cyanobacteria; Pleurocapsales;	*Chroococcidiopsis thermalis*	WP_015154928
	Xenococcus sp. PCC 7305	WP_040897270
Bacteria; Cyanobacteria; Stigonematales;	*Fischerella muscicola*	WP_016868808
		WP_016868299
	Mastigocoleus testarum	WP_027837927
		WP_027840549
Bacteria; Deferribacteres; Deferribacterales;	*Deferribacter desulfuricans*	WP_013008665
		WP_013008173
	Deferribacter desulfuricans SSM1	BAI79627
	Mucispirillum schaedleri ASF457	ESJ98052
Bacteria; Deinococcus-Thermus; Deinococci;	*Deinococcus radiodurans* R1	Q9RR76
	Deinococcus radiodurans	WP_010889102
	Deinococcus geothermalis	WP_011525920
	Thermus thermophilus	WP_014629693
		WP_014510291
	Thermus thermophilus HB8	Q5SHJ0
Bacteria; Dictyoglomi; Dictyoglomales;	*Dictyoglomus thermophilum*	WP_012548792
Bacteria; Elusimicrobia; Elusimicrobia;	*Elusimicrobium minutum*	WP_012415277
Bacteria; Fibrobacteres; Chitinivibrionia;	*Chitinivibrio alkaliphilus* ACht1	ERP31319
Bacteria; Fibrobacteres; Fibrobacterales;		WP_014546887

	Fibrobacter succinogenes	WP_014545700
Bacteria; Firmicutes; Bacilli;	*Bacillus halodurans* C-125	BAB05359
	Bacillus subtilis subsp. *subtilis* str. 168	AAA86746
	Bacillus subtilis subsp. *subtilis*	AJE96931
	Bacillus subtilis subsp. *subtilis* str. SC-8	EHA32395
	Virgibacillus halodenitrificans	CDQ35629
	Brevibacillus massiliensis	WP_019122471
		WP_019123658
	Paenibacillus curdlanolyticus YK9	EFM09788
	Staphylococcus aureus A9635	EEV71258
	Aerococcus viridans	WP_003143054
	Enterococcus faecalis	WP_016627279
	Lactobacillus casei 32G	EKQ01318
	Oenococcus kitaharae DSM 17330	EHN59105
Bacteria; Firmicutes; Clostridia;	*Clostridium ultunense* Esp	CCQ96024
	Symbiobacterium thermophilum IAM 14863	BAD40666
	Anaerofustis stercorihominis DSM 17244	EDS71456
		EDS71629
		EDS72882
	Eubacterium limosum	WP_013382372
	Heliobacterium modesticaldum	WP_012281247
	Anaerostipes caccae DSM 14662	EDR96723
	Desulfotomaculum alcoholivorax	WP_027365188
	Ruminococcus flavefaciens	WP_009984763
	Thermoanaerobacter wiegelii	WP_041589314
	Thermodesulfobium narugense	WP_013755507
Bacteria; Firmicutes; Erysipelotrichia;	*Erysipelothrix rhusiopathiae* ATCC 19414	EFY09694
	Turicibacter sanguinis PC909	EFF64393
Bacteria; Firmicutes; Negativicutes;	*Acidaminococcus intestini*	WP_026385421
	Dialister invisus	WP_007069844
	Pelosinus fermentans DSM 17108	EIW28650
		EIW33496
	Veillonella atypica KON	EKY20870
Bacteria; Fusobacteria; Fusobacteriales;	*Fusobacterium perfoetens*	WP_027129016
	Fusobacterium ulcerans	WP_005979179
	Fusobacterium nucleatum CTI-3	ERT36540
	Leptotrichia wadei F0279	ERK54075
Bacteria; Gemmatimonadetes; Gemmatimonadales;	*Gemmatimonas aurantiaca* T-27	BAH38645
		BAH39749
	Gemmatimonas aurantiaca	WP_015894566
Bacteria; Haloplasmatales; Haloplasmataceae;	*Haloplasma contractile*	WP_008825790
		WP_008824753
Bacteria; Ignavibacteriae; Ignavibacteria;	*Ignavibacterium album*	WP_014561933
		WP_014560269
	Melioribacter roseus	WP_014856496
Bacteria; Latescibacteria;	*Candidatus* Latescibacter anaerobius	WP_022817110
Bacteria; Lentisphaerae; Lentisphaeria;	*Lentisphaera araneosa* HTCC2155	EDM28342
Bacteria; Marinimicrobia	Marinimicrobia bacterium SCGC AAA298-D23	WP_022839043
Bacteria; Microgenomates	Microgenomates bacterium SCGC AAA011-L6	WP_029251330

Bacteria; Nitrospinae; Nitrospinia;	*Nitrospina gracilis* 3/211	CCQ91501
Bacteria; Nitrospirae; Nitrospirales;	*Leptospirillum ferrodiazotrophum*	EES51880
	Candidatus Nitrospira defluvii	CBK40306
	Thermodesulfovibrio thiophilus	WP_028844402
Bacteria; Omnitrophica	*Omnitrophica bacterium* SCGC AAA257-O07	WP_028483971
Bacteria; Parcubacteria;	*Candidatus* Paceibacter normanii	WP_027927187
Bacteria; Planctomycetes; Phycisphaerae;	*Phycisphaera mikurensis* NBRC 102666	BAM02304
Bacteria; Planctomycetes; Planctomycetia;	*Candidatus* Kuenenia stuttgartiensis	CAJ72443
	Candidatus Scalindua brodae	KHE91036
	Planctomyces limnophilus	WP_013109618
	Planctomyces brasiliensis	WP_013629089
	Zavarzinella formosa	WP_020472803
Bacteria; Poribacteria	*Candidatus* Poribacteria sp. WGA-4E	WP_020382503
	Poribacteria bacterium WGA-3G	WP_022814938
	Candidatus Poribacteria sp. WGA-4C	WP_041883857
Bacteria; Proteobacteria; Alphaproteobacteria;	*Caulobacter vibrioides*	WP_010920237
	Geminicoccus roseus	WP_035486379
	Magnetococcus marinus	WP_011712422
		WP_011712029
	Micavibrio aeruginosavorus	WP_014103908
	Bartonella tamiae	WP_008040187
	Mesorhizobium loti MAFF303099	BAB50933
	Rhizobium gallicum	WP_018444522
	Sinorhizobium medicae WSM419	ABR61522
		ABR61793
	Dinoroseobacter shibae	WP_012180071
		WP_012187223
	Acetobacter aceti 1023	KDE20742
	Rickettsia prowazekii str. Madrid E	NP_220931
		CAA14899
	Rickettsia prowazekii str. GvF12	EOB10299
	Sneathiella glossodoripedis	WP_025897876
	Erythrobacter litoralis	WP_011414753
Bacteria; Proteobacteria; Betaproteobacteria;	*Burkholderia rhizoxinica* HKI 454	CBW76174
	Burkholderia terrae BS001	EIN03175
	Comamonas composti	WP_027014774
	Methylibium petroleiphilum	WP_011828317
	Oxalobacter formigenes	WP_005878220
	Rubrivivax gelatinosus IL144	BAL97527
	Ferrovum myxofaciens	WP_031597515
	Tepidiphilus margaritifer	WP_028874256
	Methylophilus methylotrophus	WP_018986066
	Leeia oryzae	WP_018149894
	Neisseria shayeganii	WP_009119946
	Neisseria bacilliformis ATCC BAA-1200	EGF07155
	Nitrosomonas eutropha	WP_041353699
	Nitrosomonas europaea ATCC 19718	CAD86120
	Azospira oryzae	WP_014235438
	Sulfuricella denitrificans	WP_009207553
Bacteria; Proteobacteria; Deltaproteobacteria;	*Bacteriovorax marinus* SJ	CBW27318
	Bacteriovorax marinus	WP_014245495

	Desulfarculus baarsii	WP_013258669
	Desulfatibacillum aliphaticivorans	WP_028314015
	Desulfatibacillum alkenivorans	WP_015948404
	Desulfobacter postgatei	WP_004074082
	Desulfohalobium retbaense	WP_015752839
	Desulfonatronum lacustre	WP_028573333
	Desulfurella acetivorans A63	AHF96513
	Geobacter bemidjiensis	WP_012528449
	Geobacter metallireducens	WP_004511638
	Anaeromyxobacter dehalogenans	WP_011419455
	Myxococcus xanthus	WP_011551471
	Haliangium ochraceum	WP_012829147
	Plesiocystis pacifica SIR-1	EDM76201
	Sorangium cellulosum	WP_012235425
	Syntrophus aciditrophicus SB	ABC77734
	Syntrophobacter fumaroxidans	WP_011699536
Bacteria; Proteobacteria; Epsilonproteobacteria;	*Campylobacter gracilis* RM3268	EEV18921
	Helicobacter hepaticus ATCC 51449	AAP77391
	Helicobacter sanguini	KGI52166
	Nitratifractor salsuginis	WP_013553671
	Nautilia profundicola	WP_012663917
Bacteria; Proteobacteria; Gammaproteobacteria;	*Acidithiobacillus ferrooxidans*	WP_012607014
	Moritella marina	WP_019440661
	Candidatus Contendobacter odensis	WP_034430663
	Escherichia coli str. K-12 substr. MG1655	AAB18585
	Coxiella burnetii 'MSU Goat Q177'	EAX32839
	Methylosarcina fibrata	WP_020565869
	Pseudomonas brassicacearum	AHL34067
	Sedimenticola selenatireducens	WP_029133066
	Vibrio cholerae HE48	EGR06661
	Solimonas variicoloris	WP_026353294
	Xanthomonas translucens DAR61454	ELQ11830
Bacteria; Proteobacteria; Zetaproteobacteria;	*Mariprofundus ferrooxydans* PV-1	EAU53684
	Mariprofundus ferrooxydans	WP_009849046
Bacteria; Spirochaetes; Spirochaetales;	*Brachyspira pilosicoli* WesB	CCG57016
	Brachyspira hampsonii 30446	EKV56906
	Leptospira interrogans	WP_001206941
	Leptospira weilii str. LNT 1234	EMN44318
	Borrelia afzelii ACA-1	EEC20646
	Sphaerochaeta globosa str. Buddy	ADY12403
	Spirochaeta thermophila	WP_013312828
	Treponema caldaria	WP_013967967
Bacteria; Synergistetes; Synergistia;	*Aminobacterium colombiense*	WP_013049001
	Dethiosulfovibrio peptidovorans DSM 11002	EFC91618
	Synergistes jonesii	KEJ91636
Bacteria; Tenericutes; Mollicutes;	*Acholeplasma palmae* J233	CCV64306
	Spiroplasma mirum ATCC 29335	AHI57779
	Mycoplasma hominis ATCC 23114	CAX37303
Bacteria; Thermobaculum	*Thermobaculum terrenum*	WP_012875020
		WP_012874650
Bacteria; Thermodesulfobacteria; Thermodesulfobacteriales;	*Thermodesulfatator atlanticus*	WP_022854186
	Thermodesulfobacterium geofontis	WP_013909404
	Thermodesulfobacterium thermophilum	WP_022854988

Bacteria; Thermotogae; Thermotogales;	*Mesoaciditoga lauensis*	WP_036226255
	Marinitoga piezophila	WP_014296334
	Thermotoga maritima MSB8	1Z82_B
Bacteria; Verrucomicrobia; Opitutae;	*Coraliomargarita akajimensis*	WP_013043984
Bacteria; Verrucomicrobia; Verrucomicrobiae;	*Verrucomicrobium spinosum*	WP_029190313
	Pedosphaera parvula Ellin514	EEF57665

Supplementary Table S3. The list of sequence entries used to infer the G3PDH (GlpA/D) tree.

Taxonomy	Organism	Accession
Archaea	halophilic archaeon DL31	YP_004807559
Archaea; Crenarchaeota; Thermoprotei; Desulfurococcales;	*Aeropyrum pernix* K1	NP_147138
	Desulfurococcus kamchatkensis 1221n	YP_002428574
	Desulfurococcus fermentans DSM 16532	YP_006402373
	Staphylothermus marinus F1	YP_001040279
	Staphylothermus hellenicus DSM 12710	YP_003668585
Archaea; Crenarchaeota; Thermoprotei; Sulfolobales;	*Metallosphaera sedula* DSM 5348	YP_001191262
	Sulfolobus solfataricus P2	NP_343866
	Sulfolobus acidocaldarius DSM 639	YP_255763
		YP_256621
	Sulfolobus islandicus L.S.2.15	YP_002831137
	Sulfolobus islandicus L.D.8.5	YP_003418485
Archaea; Crenarchaeota; Thermoprotei; Thermoproteales;	*Thermofilum pendens* Hrk 5	YP_920528
		YP_920775
	Caldivirga maquilingensis IC-167	YP_001541610
	Pyrobaculum oguniense TE7	YP_005260642
	Vulcanisaeta distributa DSM 14429	YP_003900729
		YP_003901260
		YP_003901840
		YP_003901936
	Vulcanisaeta moutnovskia 768-28	YP_004243833
		YP_004245070
		YP_004245910
Archaea; Euryarchaeota; Aciduliprofundum	*Aciduliprofundum boonei* T469	YP_003483900
Archaea; Euryarchaeota; Archaeoglobi;	*Archaeoglobus fulgidus* DSM 4304	NP_070157
	Archaeoglobus veneficus SNP6	YP_004342538
Archaea; Euryarchaeota; Halobacteria; Halobacteriales;	*Halalkalicoccus jeotgali* B3	YP_003735329
		YP_003737306
	Haloarcula marismortui ATCC 43049	AAV46823
		YP_135276
	Haloarcula hispanica ATCC 33960	YP_004795864
		YP_004797041
	Halobacterium sp. NRC-1	NP_444207
	Halobacterium salinarum R1	YP_001689097
		YP_001689788
	Haloferax volcanii DS2	YP_003533725
		YP_003535585
	Haloferax mediterranei ATCC 33500	YP_006349292
	Halogeometricum borinquense DSM 11551	YP_004036697
		YP_004036704
	Halomicrobium mukohataei DSM 12286	YP_003178331
		YP_003178385
	Halopiger xanaduensis SH-6	YP_004596807
		YP_004598408

	Haloquadratum walsbyi DSM 16790	YP_657500
		YP_658392
	Haloquadratum walsbyi C23	YP_005840447
	Halorhabdus utahensis DSM 12940	YP_003129602
		YP_003130380
	Halorubrum lacusprofundi ATCC 49239	YP_002565787
	Haloterrigena turkmenica DSM 5511	YP_003404503
		YP_003406508
	Natrialba magadii ATCC 43099	YP_003479079
		YP_003481190
	Natrinema sp. J7-2	YP_006540773
		YP_006541035
Archaea; Euryarchaeota; Methanomicrobia; Methanocellales;	*Methanocella arvoryzae* MRE50	YP_687586
	Methanocella paludicola SANAE	YP_003356194
	Methanocella conradii HZ254	YP_005380331
Archaea; Euryarchaeota; Thermococci; Thermococcales;	*Pyrococcus abyssi* GE5	NP_125962
	Pyrococcus furiosus DSM 3638	NP_579734
	Pyrococcus yayanosii CH1	YP_004624760
	Pyrococcus sp. ST04	YP_006353723
	Thermococcus kodakarensis KOD1	YP_183806
	Thermococcus onnurineus NA1	YP_002306585
	Thermococcus sp. AM4	YP_002582268
	Thermococcus gammatolerans	YP_002959389
		YP_002960157
	Thermococcus sibiricus MM 739	YP_002994181
	Thermococcus barophilus MP	YP_004070427
	Thermococcus sp. 4557	YP_004761857
		YP_004762353
	Thermococcus sp. CL1	YP_006424428
Archaea; Euryarchaeota; Thermoplasmata; Thermoplasmatales;	*Ferroplasma acidarmanus* fer1	ZP_05570243
	Picrophilus torridus DSM 9790	YP_024264
	Thermoplasma volcanium GSS1	NP_111359
	Thermoplasma acidophilum DSM 1728	NP_394105
Archaea; Korarchaeota; Candidatus Korarchaeum	*Candidatus* Korarchaeum cryptofilum OPF8	YP_001736738
Bacteria; Acidobacteria; Acidobacteriales;	*Granulicella tundricola* MP5ACTX9	YP_004217492
	Terriglobus saanensis SP1PR4	YP_004184650
Bacteria; Actinobacteria; Actinobacteridae;	*Actinomyces* sp. oral taxon 175 str. F0384	ZP_08759727
	Actinomyces sp. ICM39	ZP_10767126
	Mobiluncus mulieris ATCC 35243	EEJ54743
	Mobiluncus curtisii subsp. *holmesii* ATCC 35242	ZP_07910046
	Corynebacterium glutamicum ATCC 13032	BAB99039
	Corynebacterium nuruki S6-4	ZP_09129090
	Dietzia cinnamea P4	ZP_08023186
	Gordonia sp. KTR9	YP_006671520
	Gordonia rhizosphera NBRC 16068	ZP_10944040
	Mycobacterium ulcerans Agy99	YP_906459
	Mycobacterium gilvum Spyr1	YP_004076699
	Nocardia farcinica IFM 10152	YP_117181
	Frankia alni ACN14a	YP_711818
	Frankia symbiont of *Datisca glomerata*	YP_004582733
	Brachybacterium faecium DSM 4810	YP_003156345

	Janibacter sp. HTCC2649	ZP_00993754
	Jonesia denitrificans DSM 20603	YP_003160454
	Clavibacter michiganensis subsp. *sepedonicus*	YP_001709472
	Arthrobacter phenanthrenivorans Sphe3	YP_004241971
	Micrococcus luteus NCTC 2665	YP_002958321
	Rothia dentocariosa ATCC 17931	YP_003984535
	Verrucosispora maris AB-18-032	YP_004408132
	Nocardioides sp. JS614	YP_925774
	Propionibacterium freudenreichii subsp. *shermanii* CIRM-BIA1	YP_003688243
	Propionibacterium acidipropionici ATCC 4875	YP_006981912
	Propionibacterium acnes SK182B-JCVI	ZP_12575568
	Pseudonocardia dioxanivorans CB1190	YP_004335068
	Saccharomonospora azurea NA-128	ZP_09873668
	Streptomyces avermitilis MA-4680 = NBRC 14893	NP_826180
	Streptomyces griseus subsp. *griseus* NBRC 13350	YP_001827355
	Streptomyces sp. AA4	ZP_07279229
	Streptomyces sp. Tu6071	ZP_08454657
Bacteria; Actinobacteria; Coriobacteridae;	*Atopobium rimae* ATCC 49626	ZP_03567654
	Atopobium sp. ICM58	ZP_10764468
Bacteria; Actinobacteria; Rubrobacteridae;	*Rubrobacter xylanophilus* DSM 9941	YP_643847
Bacteria; Bacteroidetes; Bacteroidetes Order II.	*Salinibacter ruber* DSM 13855	YP_446536
Bacteria; Bacteroidetes; Bacteroidia;	*Bacteroides clarus* YIT 12056	ZP_08296855
	Alistipes shahii WAL 8301	CBK64321
Bacteria; Bacteroidetes; Cytophagia;	*Cecembia lonarensis* LW9	ZP_11071538
	Fibrella aestuarina BUZ 2	CCG98659
Bacteria; Bacteroidetes; Flavobacteriia;	*Blattabacterium* sp. (*Blattella germanica*) str. Bge	YP_003284075
Bacteria; Bacteroidetes; Sphingobacteriia;	*Pedobacter saltans* DSM 12145	YP_004275252
Bacteria; Chlamydiae; Chlamydiales;	*Simkania negevensis* Z	YP_004671724
	Waddlia chondrophila 2032/99	CCB91346
Bacteria; Chloroflexi; Chloroflexales;	*Oscillochloris trichoides* DG-6	ZP_07686363
Bacteria; Chloroflexi; Thermomicrobiales;	*Thermomicrobium roseum* DSM 5159	YP_002523638
Bacteria; Cyanobacteria; Oscillatoriophycideae;	*Acaryochloris* sp. CCMEE 5410	ZP_09248308
	Cyanothece sp. ATCC 51142	YP_001804024
	Synechococcus sp. WH 7803	YP_001224269
	Arthrospira maxima CS-328	ZP_03273529
	Coleofasciculus chthonoplastes PCC 7420	ZP_05028955
Bacteria; Deferribacteres; Deferribacterales;	*Deferribacter desulfuricans* SSM1	YP_003496792
Bacteria; Deinococcus-Thermus; Deinococci;	*Deinococcus radiodurans* R1	NP_294743
	Deinococcus maricopensis DSM 21211	YP_004172422
	Marinithermus hydrothermalis DSM 14884	YP_004367519
	Thermus thermophilus HB8	YP_145382
Bacteria; Firmicutes; Bacilli;	*Bacillus subtilis* BEST7613	BAM54932
	Bacillus sp. NRRL B-14911	ZP_01169666
	Bacillus sp. B14905	ZP_01721828
	Bacillus subtilis subsp. *subtilis* str. 168	ZP_03590616
	Bacillus azotoformans LMG 9581	ZP_11313520
	Bacillus anthracis str. Ames	NP_843528
	Geobacillus thermodenitrificans NG80-2	YP_001126186
	Geobacillus sp. WCH70	YP_002950065
	Halobacillus halophilus DSM 2266	YP_006181646
	Brevibacillus brevis NBRC 100599	YP_002774517
	Paenibacillus alvei DSM 29	ZP_10866902

	Paenibacillus popilliae ATCC 14706	ZP_16293437
	Sporolactobacillus vineae DSM 21990 = SL153	ZP_10969323
	Bacillus selenitireducens MLS10	YP_003699012
		YP_003700075
	Staphylococcus aureus subsp. *aureus* MRSA252	YP_040688
	Staphylococcus hominis SK119	ZP_04059706
	Staphylococcus aureus subsp. *aureus* CIG290	ZP_13541557
	Enterococcus faecium Com15	ZP_05679719
	Lactobacillus casei ATCC 334	YP_805944
	Lactobacillus rhamnosus HN001	ZP_03210412
	Lactobacillus animalis KCTC 3501	ZP_08548560
	Lactobacillus farciminis KCTC 3681	ZP_08575922
	Lactococcus lactis subsp. *cremoris* A76	YP_005876094
	Streptococcus porcinus str. *Jelinkova* 176	ZP_08399741
	Streptococcus ictaluri 707-05	ZP_09126963
	Streptococcus oralis SK100	ZP_14306472
Bacteria; Firmicutes; Clostridia;	*Clostridiales genomosp.* BVAB3 str. UPII9-5	YP_003475727
	Clostridium perfringens WAL-14572	ZP_16428527
	Sulfobacillus acidophilus TPY	YP_004720861
	Acetobacterium woodii DSM 1030	YP_005269444
	Eubacterium limosum KIST612	YP_003961020
	Heliobacterium modesticaldum Ice1	YP_001680178
	Lachnospiraceae bacterium 3_1_57FAA_CT1	ZP_08610514
	Desulfotomaculum nigrificans DSM 574	ZP_08113214
	Symbiobacterium thermophilum IAM 14863	YP_074256
		YP_075815
	Halanaerobium hydrogeniformans	YP_003993864
	Halanaerobium praevalens DSM 2228	YP_005836013
	Caldanaerobacter subterraneus subsp. *tengcongensis* MB4	NP_623575
	Carboxydothermus hydrogenoformans Z-2901	YP_360658
	Thermoanaerobacter siderophilus SR4	ZP_10305078
Bacteria; Firmicutes; Erysipelotrichia;	Erysipelotrichaceae bacterium 6_1_45	ZP_16409751
Bacteria; Firmicutes; Negativicutes;	*Pelosinus fermentans* B4	ZP_15516389
	Selenomonas sp. CM52	ZP_10883299
	Thermosinus carboxydivorans Nor1	ZP_01666923
Bacteria; Fusobacteria; Fusobacteriales;	*Fusobacterium nucleatum* subsp. *fusiforme* ATCC 51190	ZP_15604212
	Fusobacterium nucleatum subsp. *polymorphum* F0401	ZP_16419585
	Ilyobacter polytropus DSM 2926	YP_003967859
Bacteria; Planctomycetes; Planctomycetia;	*Rhodopirellula baltica* SH 1	NP_865708
	Singulisphaera acidiphila DSM 18658	ZP_09571198
Bacteria; Proteobacteria; Alphaproteobacteria;	*Bartonella grahamii* as4aup	YP_002972071
	Bradyrhizobiaceae bacterium SG-6C	ZP_08627298
	Bradyrhizobium sp. BTAi1	YP_001238306
	Bradyrhizobium sp. WSM1253	ZP_10082831
	Ochrobactrum anthropi CTS-325	ZP_10967898
	Methylobacterium radiotolerans JCM 2831	YP_001754857
	Agrobacterium sp. ATCC 31749	ZP_08530727
	Rhizobium lupini HPC(L)	ZP_11198308
	Sinorhizobium meliloti SM11	YP_005723462
	Hirschia baltica ATCC 49814	YP_003061191
	Celeribacter baekdonensis B30	ZP_11131853
	Labrenzia alexandrii DFL-11	ZP_05115288
	Octadecabacter arcticus 238	ZP_05065378

	Pelagibaca bermudensis HTCC2601	ZP_01441100
	Roseibium sp. TrichSKD4	ZP_07659307
	Silicibacter lacuscaerulensis ITI-1157	ZP_05787968
	Acidiphilium multivorum AIU301	YP_004285128
	Gluconacetobacter oboediens 174Bp2	ZP_08898651
	Acetobacteraceae bacterium AT-5844	ZP_09397651
	Thalassospira profundimaris WP0211	ZP_11121319
	Tistrella mobilis KA081020-065	YP_006374570
	Sphingomonas elodea ATCC 31461	ZP_09958823
	Sphingomonas sp. PAMC 26617	ZP_10425396
	Sphingopyxis alaskensis RB2256	YP_615246
Bacteria; Proteobacteria; Betaproteobacteria;	*Achromobacter piechaudii* ATCC 43553	ZP_06688535
	Bordetella pertussis Tohama I	NP_881260
	Burkholderia rhizoxinica HKI 454	YP_004027742
	Cupriavidus metallidurans CH34	YP_587572
	Ralstonia solanacearum UW551	ZP_00946852
	Acidovorax sp. NO-1	ZP_09330941
	Verminephrobacter eiseniae EF01-2	YP_996788
	Leptothrix cholodnii SP-6	YP_001792244
	Sideroxydans lithotrophicus ES-1	YP_003525071
	Pseudogulbenkiania sp. NH8B	YP_004849272
	Simonsiella muelleri ATCC 29453	ZP_10984245
	Azoarcus sp. KH32C	BAL23536
Bacteria; Proteobacteria; Deltaproteobacteria	delta proteobacterium NaphS2	ZP_07205020
	Desulfobacterium autotrophicum HRM2	YP_002601608
	Desulfobacula toluolica Tol2	YP_006761709
	Desulfovibrio vulgaris str. Hildenborough	YP_011157
		YP_011885
	Desulfovibrio desulfuricans subsp. *desulfuricans* str. ATCC 27774	YP_002480101
	Geobacter sp. M18	YP_004200166
	Anaeromyxobacter sp. Fw109-5	YP_001378528
Bacteria; Proteobacteria; Epsilonproteobacteria;	*Helicobacter felis* ATCC 49179	YP_004073920
Bacteria; Proteobacteria; Gammaproteobacteria	gamma proteobacterium HdN1	YP_003812610
	Aeromonas caviae Ae398	ZP_08521014
	Glaciecola psychrophila 170	ZP_11323085
	Marinobacter sp. BSs20148	YP_006558769
	Marinobacter sp. ELB17	ZP_01739057
	Moritella sp. PE36	ZP_01897076
	Pseudoalteromonas sp. Bsw20308	ZP_11405940
	Citrobacter rodentium ICC168	YP_003367805
	Citrobacter freundii 4_7_47CFAA	ZP_09336341
	Enterobacter sp. 638	YP_001178541
	Escherichia coli	2R4J_A
	Escherichia coli K-12	P0A9C0
	Morganella morganii SC01	ZP_11283921
	Photorhabdus luminescens subsp. *laumondii* TTO1	NP_927559
	Salmonella enterica subsp. *enterica* serovar Paratyphi B str. SPB7	YP_001590530
	Salmonella enterica subsp. *enterica* serovar Heidelberg str. SL476	YP_002046338
	Yersinia frederiksenii ATCC 33641	ZP_04632780
	Legionella pneumophila str. Lens	YP_126714

	Marinomonas posidonica IVIA-Po-181	YP_004480354
	Actinobacillus minor 202	ZP_05628993
	Aggregatibacter actinomycetemcomitans serotype e str. SC1083	ZP_11577631
	Haemophilus influenzae PittGG	YP_001292612
	Pasteurella bettyae CCUG 2042	ZP_10125760
	Acinetobacter sp. NCTC 10304	ZP_10936832
	Pseudomonas sp. S9	ZP_09709455
	Pseudomonas sp. GM102	ZP_10596622
	Pseudomonas sp. GM55	ZP_10645168
	Francisella tularensis subsp. *mediasiatica* FSC147	YP_001891056
	Grimontia hollisae CIP 101886	ZP_06053660
	Photobacterium profundum SS9	YP_129585
	Vibrio parahaemolyticus RIMD 2210633	NP_798767
	Vibrio vulnificus MO6-24/O	YP_004190689
	Vibrio caribbenthicus ATCC BAA-2122	ZP_07743946
	Stenotrophomonas maltophilia JV3	YP_004794105
	Xanthomonas axonopodis pv. *punicae* str. LMG 859	ZP_10261464
	Xylella fastidiosa 9a5c	NP_299545
Bacteria; Spirochaetes; Spirochaetales;	*Brachyspira pilosicoli* WesB	CCG57584
	Leptospira broomii serovar Hurstbridge str. 5399	ZP_10531998
	Leptospira interrogans str. UI 08452	ZP_13277471
	Leptospira interrogans str. Brem 329	ZP_15582285
	Turneriella parva DSM 21527	YP_006439233
	Borrelia burgdorferi	ABQ43062
	Borrelia burgdorferi ZS7	ABQ43065
	Borrelia valaisiana VS116	ZP_03672365
	Spirochaeta smaragdinae DSM 11293	YP_003805225
	Treponema phagedenis F0421	ZP_08036972
Bacteria; Synergistetes; Synergistia;	*Pyramidobacter piscolens* W5455	ZP_06265733
Bacteria; Tenericutes; Mollicutes;	*Mycoplasma hyorhinis* HUB-1	YP_003856265
	Mycoplasma mycoides subsp. *capri* LC str. 95010	YP_004399986
	Mycoplasma pneumoniae 309	YP_005175284
Bacteria; Thermotogae; Thermotogales;	*Kosmotoga olearia* TBF 19.5.1	YP_002939889
	Marinitoga piezophila KA3	YP_005096443
	Thermosipho melanesiensis BI429	YP_001305869
	Thermotoga maritima MSB8	ZP_12683583

Supplementary Table S4. The list of sequence entries used to infer the GK (GlpK) tree.

Taxonomy	Organism	Accession
Archaea; Aigarchaeota	Aigarchaeota archaeon JGI 0000106-J15	WP_042660898
Archaea; Aigarchaeota	Aigarchaeota archaeon JGI 0000106-J15	WP_042660900
Archaea; Aigarchaeota	Thaumarchaeota archaeon JGI OTU-3	WP_042663342
Archaea; Crenarchaeota; Thermoprotei; Desulfurococcales;	*Aeropyrum pernix* K1	BAA79261
Archaea; Crenarchaeota; Thermoprotei; Desulfurococcales;	*Desulfurococcus fermentans* DSM 16532	AFL66797
Archaea; Crenarchaeota; Thermoprotei; Desulfurococcales;	*Staphylothermus marinus* F1	ABN69377
Archaea; Crenarchaeota; Thermoprotei; Sulfolobales;	*Sulfolobus acidocaldarius* DSM 639	AAY80469
Archaea; Crenarchaeota; Thermoprotei; Sulfolobales;	*Sulfolobus solfataricus* P2	NP_343018
Archaea; Crenarchaeota; Thermoprotei; Thermoproteales;	*Thermofilum pendens* Hrk 5	ABL78526
Archaea; Crenarchaeota; Thermoprotei; Thermoproteales;	*Thermofilum pendens* Hrk 5	ABL78770
Archaea; Crenarchaeota; Thermoprotei; Thermoproteales;	*Pyrobaculum oguniense*	WP_014347509
Archaea; Crenarchaeota; Thermoprotei; Thermoproteales;	*Vulcanisaeta distributa* DSM 14429	ADN49680
Archaea; Crenarchaeota; Thermoprotei; Thermoproteales;	*Vulcanisaeta distributa* DSM 14429	ADN50882
Archaea; Euryarchaeota;	*Aciduliprofundum boonei* T469	ADD09236
Archaea; Euryarchaeota; Archaeoglobi; Archaeoglobales;	*Archaeoglobus fulgidus* DSM 4304	AAB90370
Archaea; Euryarchaeota; Archaeoglobi; Archaeoglobales;	*Archaeoglobus fulgidus* DSM 8774	AIG98753
Archaea; Euryarchaeota; candidate division pMC2A384	Euryarchaeota archaeon SCGC AAA252-I15	WP_041899301
Archaea; Euryarchaeota; environmental samples	uncultured marine group II/III euryarchaeote KM3_86_F07	AIF19344
Archaea; Euryarchaeota; Halobacteria; Halobacteriales	halophilic archaeon J07HX5	ERG88035
Archaea; Euryarchaeota; Halobacteria; Halobacteriales	*Halarchaeum acidiphilum* MH1-52-1	GAD52556
Archaea; Euryarchaeota; Halobacteria; Halobacteriales	*Halalkalicoccus jeotgali* B3	ELY36074
Archaea; Euryarchaeota; Halobacteria; Halobacteriales	*Halobacterium salinarum* R1	B0R6S2
Archaea; Euryarchaeota; Halobacteria; Halobacteriales	*Halococcus saccharolyticus* DSM 5350	EMA43995
Archaea; Euryarchaeota; Halobacteria; Halobacteriales	*Haloferax volcanii* DS2	ADE04400
Archaea; Euryarchaeota; Halobacteria; Halobacteriales	*Halorhabdus tiamatea* SARL4B	CCQ34342
Archaea; Euryarchaeota; Halobacteria; Halobacteriales	*Halorhabdus tiamatea* SARL4B	ERJ06771
Archaea; Euryarchaeota; Halobacteria; Halobacteriales	*Halosimplex carlsbadense* 2-9-1	ELZ24248
Archaea; Euryarchaeota; Halobacteria; Halobacteriales	*Natronomonas moolapensis* 8.8.11	CCQ35593
Archaea; Euryarchaeota; Halobacteria; Halobacteriales	*Natronomonas moolapensis* 8.8.11	CCQ37762
Archaea; Euryarchaeota; Methanomicrobia;	*Methanosaeta thermophila*	WP_011696420
Archaea; Euryarchaeota; Thermococci; Thermococcales;	*Palaeococcus pacificus* DY20341	AIF68948
Archaea; Euryarchaeota; Thermococci; Thermococcales;	*Palaeococcus pacificus* DY20341	AIF70086
Archaea; Euryarchaeota; Thermococci; Thermococcales;	*Pyrococcus abyssi* GE5	CCE69644
Archaea; Euryarchaeota; Thermococci; Thermococcales;	*Thermococcus kodakarensis* KOD1	BAA34909
Archaea; Euryarchaeota; Thermoplasmata; Thermoplasmatales;	*Acidiplasma* sp. MBA-1	KJE50097
Archaea; Euryarchaeota; Thermoplasmata; Thermoplasmatales;	*Ferroplasma acidarmanus* fer1	AGO61823
Archaea; Euryarchaeota; Thermoplasmata; Thermoplasmatales;	*Picrophilus torridus* DSM 9790	CBG09962
Archaea; Euryarchaeota; Thermoplasmata; Thermoplasmatales;	*Thermoplasma acidophilum* DSM 1728	CBG09912
Archaea; Euryarchaeota; Thermoplasmata; Thermoplasmatales;	*Thermoplasma volcanium* GSS1	BAB60308
Archaea; Korarchaeota; Candidatus Korarchaeum	*Candidatus* Korarchaeum cryptofilum OPF8	ACB07057
Archaea; Lokiarchaeota; 'Lokiarchaeum'	archaeon Loki	KKK40876
Archaea; Parvarchaeota; Candidatus Parvarchaeum	*Candidatus* Parvarchaeum acidophilus ARMAN-5	EFD93071
Archaea; Thaumarchaeota; unclassified Thaumarchaeota	*Candidatus* Caldiarchaeum subterraneum	BAJ46744
Bacteria; Acetothermia	Acetothermia bacterium SCGC AAA255-C06	WP_029957707
Bacteria; Acidobacteria; Acidobacteriales; Acidobacteriaceae;	*Acidobacterium capsulatum* ATCC 51196	ACO34171
Bacteria; Acidobacteria; Acidobacteriales; Acidobacteriaceae;	*Edaphobacter aggregans*	WP_035358925
Bacteria; Acidobacteria; Acidobacteria subdivision 4;	*Chloracidobacterium thermophilum*	WP_014099661
Bacteria; Acidobacteria;	*Candidatus* Koribacter versatilis Ellin345	ABF41988
Bacteria; Acidobacteria; Solibacteres;	*Candidatus* Solibacter usitatus Ellin6076	ABJ86502

	Bryobacter aggregatus	WP_031495195
Bacteria; Actinobacteria; Acidimicrobidae;	*Acidimicrobium ferrooxidans* DSM 10331	ACU53821
		ACU54395
Bacteria; Actinobacteria; Acidimicrobiia;	*Acidithrix ferrooxidans*	KJF16125
	Ilumatobacter nonamiensis	WP_040492385
		WP_040494833
Bacteria; Actinobacteria;	*Candidatus* Microthrix parvicella RN1	CCM62868
		CCM63116
Bacteria; Actinobacteria; Catenulisporales;	*Actinospica robiniae*	WP_034270735
Bacteria; Actinobacteria; Coriobacteridae;	*Coriobacterium glomerans* PW2	AEB06307
		AEB07709
	Cryptobacterium curtum DSM 15641	ACU94901
Bacteria; Actinobacteria; Coriobacteriia;	*Collinsella stercoris* DSM 13279	EEA91608
	Eggerthella lenta 1_1_60AFAA	KGI71311
Bacteria; Actinobacteria; Corynebacteriales;	*Nocardia cyriacigeorgica* GUH-2	CCF61844
		CCF63523
		CCF63525
Bacteria; Actinobacteria; Frankiales;	*Frankia alni* ACN14a	CAJ63208
Bacteria; Actinobacteria; Geodermatophilales;	*Modestobacter marinus*	WP_014741070
Bacteria; Actinobacteria; Micrococcales;	*Demetria terragena*	WP_018157864
	Knoellia aerolata DSM 18566	KGN40074
	Agrococcus pavilionensis RW1	ERG63217
		ERG64268
	Glaciibacter superstes	WP_022885734
	Gulosibacter molinativorax	WP_026935893
Bacteria; Actinobacteria; Micromonosporales;	*Micromonospora lupini*	WP_007455927
		WP_039906841
Bacteria; Actinobacteria; Propionibacteriales;	*Aeromicrobium marinum* DSM 15272	EFQ83688
	Marmoricola aequoreus	WP_030484549
		WP_030486057
		WP_030486066
Bacteria; Actinobacteria; Pseudonocardiales;	*Actinomycetospora chiangmaiensis*	WP_018333955
Bacteria; Actinobacteria; Rubrobacteria;	*Rubrobacter xylanophilus* DSM 9941	CBG09994
Bacteria; Actinobacteria; Rubrobacteridae;	*Conexibacter woesei* DSM 14684	ADB52334
Bacteria; Actinobacteria; Streptomycetales;	*Streptomyces coelicolor* A3(2)	CBG09919
		NP_625935
Bacteria; Actinobacteria; Thermoleophilia;	*Patulibacter americanus*	WP_022926992
		WP_022928288
	Solirubrobacter soli	WP_028064102
		WP_028066920
		WP_037503402
Bacteria; Aerophobetes;	*Candidatus* Aerophobus profundus	WP_041887985
		WP_041888568
		WP_041889415
		WP_041891372
Bacteria; Aquificae; Aquificales;	*Aquifex aeolicus* VF5	AAC06710
Bacteria; Armatimonadetes; Chthonomonadetes;	*Chthonomonas calidirosea*	WP_016483400
Bacteria; Armatimonadetes;	*Fimbriimonas ginsengisoli* Gsoil 348	AIE83569
Bacteria; Atribacteria;	Atribacteria bacterium JGI 0000059-I14	WP_020264864
	Atribacteria bacterium SCGC AAA255-G05	WP_029717935
	Atribacteria bacterium SCGC AAA255-N14	WP_029955352
	Atribacteria bacterium SCGC AB-164-A22	WP_038960502
		WP_038960838

	Candidatus Caldatribacterium saccharofermentans	WP_017873041
		WP_017873216
		WP_017873430
		WP_017873676
		WP_017874083
		WP_038304983
		WP_038305191
Bacteria; Bacteroidetes; Bacteroidetes Order II.	*Salinibacter ruber* DSM 13855	ABC43677
	Salisaeta longa	WP_022834800
Bacteria; Bacteroidetes; Bacteroidia;	*Bacteroides pectinophilus* CAG:437	CDD58737
	Draconibacterium orientale	AHW60297
	Anaerophaga thermohalophila	WP_016775946
	Odoribacter laneus CAG:561	CCZ79985
	Prolixibacter bellariivorans	WP_025864903
Bacteria; Bacteroidetes; Cytophagia;	*Cyclobacterium marinum* DSM 745	AEL26147
	Flexibacter elegans	WP_027000030
	Flammeovirga pacifica	WP_044222896
Bacteria; Bacteroidetes; Flavobacteriia;	*Arenibacter latericius*	WP_026809172
		WP_026809319
Bacteria; Bacteroidetes; Sphingobacteriia;	*Niastella koreensis* GR20-10	AEV96471
		AEV97103
	Saprospira grandis DSM 2844	EJF54771
	Sphingobacterium spiritivorum ATCC 33300	EEI90335
Bacteria; Caldiserica; Caldisericia;	*Caldisericum exile* AZM16c01	BAL81566
Bacteria; candidate division BRC1	BRC1 bacterium SCGC AAA252-M09	WP_020249390
	BRC1 bacterium SCGC AAA257-C11	WP_029711590
		WP_029712137
Bacteria; Chlamydiae; Chlamydiales;	*Chlamydia trachomatis*	CRH65459
		CRH83989
	Simkania negevensis Z	CCB89231
	Waddlia chondrophila WSU 86-1044	ADI38647
Bacteria; Chlorobi; Chlorobia;	*Chlorobium ferrooxidans* DSM 13031	EAT59240
Bacteria; Chloroflexi; Anaerolineae;	*Anaerolinea thermophila* UNI-1	BAJ64787
Bacteria; Chloroflexi; Caldilineae;	*Caldilinea aerophila* DSM 14535 = NBRC 104270	BAL98714
		BAL98869
		BAM01359
Bacteria; Chloroflexi; Chloroflexia;	*Chloroflexus aggregans* DSM 9485	ACL23025
		ACL26565
	Herpetosiphon aurantiacus DSM 785	ABX03446
Bacteria; Chloroflexi; Ktedonobacteria;	*Ktedonobacter racemifer* DSM 44963	EFH86008
Bacteria; Chloroflexi; Sphaerobacteridae;	*Sphaerobacter thermophilus* DSM 20745	ACZ40521
Bacteria; Chloroflexi;	*Thermorudis peleae*	WP_038037768
Bacteria; Cyanobacteria; Gloeobacteria;	*Gloeobacter violaceus* PCC 7421	BAC89692
Bacteria; Cyanobacteria; Nostocales;	*Hassallia byssoidea* VB512170	KIF30382
		KIF33408
	Tolypothrix campylonemoides	WP_041032975
	Anabaena cylindrica	WP_015217632
	Nostoc punctiforme PCC 73102	ACC80253
	Scytonema millei VB511283	KIF24116
Bacteria; Cyanobacteria; Oscillatoriophycideae;	*Cyanobacterium aponinum* PCC 10605	AFZ53899
	Crinalium epipsammum PCC 9333	AFZ11677
	Leptolyngbya boryana	WP_017290955
	Lyngbya confervoides BDU141951	KIF41412

	Oscillatoria acuminata PCC 6304	AFY82853
Bacteria; Cyanobacteria; Stigonematales;	*Mastigocoleus testarum*	WP_027840683
Bacteria; Deferribacteres; Deferribacterales;	*Deferribacter desulfuricans* SSM1	BAI80698
	Flexistipes sinusarabici DSM 4947	AEI15226
	Mucispirillum schaedleri ASF457	ESJ97697
Bacteria; Deinococcus-Thermus; Deinococci;	*Deinococcus radiodurans* R1	AAF11475
	Truepera radiovictrix DSM 17093	ADI13588
		ADI15622
	Thermus thermophilus	BAA28283
		WP_011229157
Bacteria; Dictyoglomi; Dictyoglomales;	*Dictyoglomus thermophilum* H-6-12	ACI19444
Bacteria; Elusimicrobia; Elusimicrobia;	*Elusimicrobium minutum* Pei191	ACC98622
Bacteria; Fibrobacteres; Fibrobacterales;	*Fibrobacter succinogenes*	WP_012820140
Bacteria; Firmicutes; Bacilli;	*Alicyclobacillus macrosporangiidus*	WP_029421137
		WP_029421276
		WP_029422805
	Bacillus subtilis subsp. *subtilis* str. 168	AIY92216
	Bacillus cereus ATCC 4342	AJH72057
	Thermicanus aegyptius	WP_028986224
	Exiguobacterium acetylicum	WP_029340955
		WP_029341249
	Jeotgalicoccus marinus	WP_026867402
	Listeria monocytogenes EGD-e	NP_464559
		NP_465063
	Sporolactobacillus terrae	WP_028977622
	Staphylococcus aureus subsp. *aureus* DSM 20231	ELP29126
	Thermoactinomyces daqus	WP_033101575
		WP_033102324
	Carnobacterium gallinarum	WP_034559134
		WP_034561444
	Lactobacillus casei 32G	EKP97237
	Lactococcus piscium MKFS47	CEN27344
		CEN28360
Bacteria; Firmicutes; Clostridia;	*Caldicoprobacter oshimai*	WP_025746882
	Catabacter hongkongensis	KKI52366
		WP_046442552
		WP_046442557
		WP_046442788
		WP_046443512
		WP_046443675
		WP_046443790
	Clostridium novyi NT	ABK60806
		ABK62551
	Kallipyga massiliensis	WP_034578917
	Anaerovorax odorimutans	WP_027399349
	Sulfobacillus thermosulfidooxidans	WP_028962118
	Symbiobacterium thermophilum IAM 14863	BAD40180
	Eubacterium hallii CAG:12	CDB17460
		CDB17563
		CDB18741
		CDB19038
		WP_022169388
	Heliobacterium modesticaldum Ice1	ABZ84166

	Lachnospira multipara	WP_027431885
	Oscillibacter ruminantium	WP_040659376
		WP_040662368
	Peptoclostridium difficile P78	EQK08286
		EQK09940
	Proteocatella sphenisci	WP_028830227
	Anaerotruncus colihominis DSM 17241	EDS11102
	Halanaerobium hydrogeniformans	WP_013404615
		WP_013405222
		WP_013405552
		WP_013405669
	Halobacteroides halobius DSM 5150	AGB41942
	Natranaerobius thermophilus JW/NM-WN-LF	ACB86230
	Thermoanaerobacterium thermosaccharolyticum DSM 571	ADL68147
	Coprothermobacter proteolyticus DSM 5265	ACI18093
Bacteria; Firmicutes; Erysipelotrichia;	*Erysipelothrix rhusiopathiae* ATCC 19414	EFY08374
Bacteria; Firmicutes; Negativicutes;	*Phascolarctobacterium succinatutens* YIT 12067	EFY05565
	Mitsuokella jalaludinii	WP_036378368
		WP_036378404
Bacteria; Fusobacteria; Fusobacteriales; Sebaldella	*Cetobacterium somerae* ATCC BAA-474	ERT68679
	Fusobacterium nucleatum CC53	EMP16676
	Ilyobacter polytropus DSM 2926	ADO81807
	Psychrilyobacter atlanticus	WP_028855010
	Leptotrichia goodfellowii F0264	EEY35146
	Sebaldella termitidis ATCC 33386	ACZ08914
Bacteria; Gemmatimonadetes; Gemmatimonadales;	*Gemmatimonas aurantiaca* T-27	BAH37860
Bacteria; Haloplasmatales; Haloplasmataceae;	*Haloplasma contractile* SSD-17B	ERJ11633
Bacteria; Lentisphaerae; Lentisphaeria;	*Lentisphaera araneosa* HTCC2155	EDM24862
Bacteria; Planctomycetes; Phycisphaerae;	*Phycisphaera mikurensis* NBRC 102666	BAM04495
Bacteria; Planctomycetes; Planctomycetia;	*Gemmata obscuriglobus*	WP_010040912
	Planctopirus limnophila DSM 3776	ADG67832
	Singulisphaera acidiphila DSM 18658	AGA27507
Bacteria; Poribacteria	*Candidatus* Poribacteria sp. WGA-4E	WP_020382788
	Poribacteria bacterium WGA-3G	WP_022813357
Bacteria; Proteobacteria; Alphaproteobacteria;	*Candidatus* Phaeomarinobacter ectocarpi	CDO58334
	Geminicoccus roseus	WP_027133847
		WP_027133908
		WP_027135507
	Kiloniella spongiae	KLN62498
	Kordiimonas gwangyangensis	WP_025896661
	Micavibrio aeruginosavorus EPB	AGH97359
	Parvularcula bermudensis HTCC2503	ADM10411
	Polymorphum gilvum SL003B-26A1	ADZ69494
	Aureimonas ureilytica	WP_019995749
	Bartonella tamiae Th239	EJF90772
	Beijerinckia indica subsp. *indica* ATCC 9039	ACB94033
	Bradyrhizobium japonicum SEMIA 5079	AHY56740
	Devosia geojensis	KKB07069
	Methyloceanibacter caenitepidi	WP_045370012
	Methylocystis parvus	WP_016921063
	Lutibaculum baratangense AMV1	ESR23373
	Acetobacter aceti NBRC 14818	GAN57735
		GAN58394

	Rhodospirillum rubrum ATCC 11170	ABC22809
		ABC24255
	Reyranella massiliensis	WP_020699470
	Candidatus Pelagibacter ubique HIMB083	ETA70016
	Candidatus Puniceispirillum marinum IMCC1322	ADE39127
	Erythrobacter longus	KEO90226
Bacteria; Proteobacteria; Betaproteobacteria;	*Aquincola tertiaricarbonis*	WP_046115146
	Burkholderia nodosa	WP_028203060
		WP_028207051
	Delftia acidovorans SPH-1	ABX33669
		ABX33766
	Parasutterella excrementihominis CAG:233	CCX86848
	Sutterella parvirubra YIT 11816	EHY30626
	Thiomonas intermedia K12	ADG29963
	Candidatus Accumulibacter phosphatis clade IIA str. UW-1	ACV35626
	Sideroxydans lithotrophicus ES-1	ADE12683
	Thiobacillus thioparus	WP_018506836
	Conchiformibius steedae	WP_027021423
	Nitrosomonas eutropha C91	ABI60303
	Nitrosospira lacus	CCU63268
	Uliginosibacterium gangwonense	WP_018609192
Bacteria; Proteobacteria; Deltaproteobacteria;	*Bdellovibrio bacteriovorus* HD100	CBG09937
	Halobacteriovorax marinus SJ	CBW28167
	Desulfarculus baarsii DSM 2075	ADK85622
	Desulfobulbus japonicus	WP_028579727
		WP_028581428
	Desulfovibrio inopinatus	WP_027184207
	Geobacter lovleyi SZ	ACD96401
	Geopsychrobacter electrodiphilus	WP_020674827
	Pelobacter seleniigenes	WP_029914222
	Anaeromyxobacter dehalogenans 2CP-C	ABC83224
	Enhygromyxa salina	KIG17860
	Haliangium ochraceum DSM 14365	ACY12932
	Sandaracinus amylolyticus	AKF05413
Bacteria; Proteobacteria; Epsilonproteobacteria;	*Arcobacter nitrofigilis* DSM 7299	ADG92542
	Helicobacter bizzozeronii CCUG 35545	CCF81739
Bacteria; Proteobacteria; Gammaproteobacteria;	*Thiobacillus prosperus*	KFZ89198
	Aeromonas caviae	KGY79666
	Anaerobiospirillum succiniciproducens	WP_027939739
	Aliagarivorans marinus	WP_026970640
		WP_035480402
	Gilvimarinus agarilyticus	WP_041524117
	Colwellia psychrerythraea	KGJ91175
	Ferrimonas futtsuensis	WP_028108982
		WP_028109277
	Cardiobacterium valvarum F0432	EHM55950
	Nitrococcus mobilis Nb-231	EAR23033
	Candidatus Competibacter denitrificans Run_A_D11	CDI01750
	Escherichia coli str. K-12 substr. MG1655	AAB03058
	Coxiella burnetii RSA 331	ABX77758
	Tatlockia micdadei	CEG61109
	Methylobacter luteus	WP_027157124
	Methylocaldum szegediense	WP_026610635

	Methyloglobulus morosus KoM1	ESS74003
	Methylohalobius crimeensis	WP_022949983
	Alcanivorax jadensis T9	KGD60522
	Balneatrix alpica	WP_027313529
	Halomonas zincidurans	WP_031383819
	Marinomonas profundimaris	ETI59307
	Oceanobacter kriegii	WP_028294836
	Congregibacter litoralis KT71	EAQ99339
	Porticoccus hydrocarbonoclasticus	WP_036860721
	Enhydrobacter aerosaccus SK60	EEV22411
	Pseudomonas alcaligenes NBRC 14159	GAD63814
	Salinisphaera hydrothermalis C41B8	KEZ76838
	Sedimenticola selenatireducens	WP_029132444
	Simiduia agarivorans SA1 = DSM 21679	AFV00906
	Fangia hongkongensis	WP_026196563
	Francisella noatunensis	WP_014714671
	Piscirickettsia salmonis LF-89 = ATCC VR-1361	ERL61157
	Thiothrix flexilis	WP_020558557
	Algiphilus aromaticivorans	WP_043769594
	Xanthomonas fuscans	WP_007968439
Bacteria; Spirochaetes; Brachyspirales;	*Brachyspira alvinipulli*	WP_028328769
Bacteria; Spirochaetes; Leptospirales;	*Leptonema illini* DSM 21528	EHQ06486
	Leptospira noguchii str. Hook	EMS83915
		EMS89436
	Turneriella parva DSM 21527	AFM14818
Bacteria; Spirochaetes; Spirochaetales;	*Borrelia afzelii* PKo	AEL69469
	Salinispira pacifica	AHC14043
		WP_024268781
	Spirochaeta smaragdinae DSM 11293	ADK80159
		ADK80772
		ADK82629
		ADK82890
	Treponema socranskii	WP_016520350
		WP_016520711
		WP_038080401
Bacteria; Synergistetes; Synergistia;	*Aminomonas paucivorans*	WP_006301602
	Synergistes sp. 3_1_syn1	EHL64432
Bacteria; Tenericutes; Mollicutes;	*Acholeplasma brassicae*	CCV65093
		CCV65925
Bacteria;	*Thermobaculum terrenum* ATCC BAA-798	ACZ41391
Bacteria; Thermotogae; Kosmotogales;	*Mesotoga prima* MesG1.Ag.4.2	AFK06476
		AFK08044
Bacteria; Thermotogae; Petrotogales;	*Defluviitoga tunisiensis*	CEP78158
Bacteria; Thermotogae; Thermotogales;	*Fervidobacterium pennivorans* DSM 9078	AFG34289
		AFG34359
	Kosmotoga olearia TBF 19.5.1	ACR79254
	Marinitoga piezophila KA3	AEX84915
	Petrotoga mobilis SJ95	ABX31026
		ABX31179
	Pseudothermotoga thermarum DSM 5069	AEH51076
	Thermosipho africanus TCF52B	ACJ75139
	Thermotoga maritima MSB8	AGL49879
		Q9X1E4

Bacteria; Verrucomicrobia; Opitutae;	*Opitutus terrae* PB90-1	ACB74772
	Coraliomargarita akajimensis DSM 45221	ADE55240
Bacteria; Verrucomicrobia; unclassified Verrucomicrobia;	*Methylacidiphilum infernorum* V4	ACD83757
Bacteria; Verrucomicrobia; Verrucomicrobiae;	Verrucomicrobiae bacterium DG1235	EDY80837
		WP_040900133
	Verrucomicrobium spinosum	WP_009960321
	Pedosphaera parvula Ellin514	EEF62012

Supplementary Table S5. Statistical test showing a maximum likelihood analysis of G1PDH.

Archaeal G1PDH (EgsA)								Bacterial G1PDH (AraM)			au	np
Crenarchaeota						Thaum -archaeota	Eury -archaeota	*Bacillus subtilis* subsp. *subtilis* str. 168	Deltaproteo -bacteria /Halo -plasmatales /*Anoxybacillus flavithermus* WK1 & *Bacillus cellulosilyticus* DSM 2522	Gamma -proteo -bacteria /Actino -bacteria		
Thermoproteales		Desulfurococcales & Acidilobales			Sulfolobales							
Most Thermo -proteales	*Thermofilum pendens* Hrk-5	Most Desulfuroco -ccales	*Acidilobus saccharovorans* /*Aeropyrum pernix* K1	*Ignisphaera aggregans* DSM 17230								
											0.879	0.744
											0.818	0.412
											**0.733**	0.307
											0.717	0.308
											0.699	0.594
											0.697	0.731
											0.697	0.731
											0.674	0.425
											0.604	0.135
											0.599	0.249
											0.589	0.271
											0.476	0.193
											0.454	0.071
											0.428	0.217
											0.413	0.166
											0.391	0.081
											0.39	0.102
											0.376	0.114
											0.372	0.06
											0.355	0.034
											0.35	0.155
											0.332	0.044
											0.311	0.253
											0.311	0.253
											0.31	0.107
											0.305	0.054
											0.303	0.025
											**0.301**	0.406
											0.289	0.058
											0.274	0.043
											0.262	0.046
											0.257	0.017
											0.256	0.05

											0.25	0.028
											0.25	0.026
											0.231	0.057
											0.23	0.059
											0.226	0.012
											0.226	0.05
											0.225	0.021
											0.217	0.035
											0.213	0.033
											0.21	0.017
											0.208	0.019
											0.198	0.006
											0.194	0.036
											0.172	0.02
											0.171	0.02
											0.163	0.046
											0.159	0.021
											0.159	0.008
											0.127	0.012
											0.117	0.005
											0.116	0.009
											0.113	0.028
											0.103	0.003
											0.101	0.002
											0.096	0.001
											0.095	0.001
											0.094	0.005
											0.016	2.00E-04

The AU test [34] was performed using Consel v0.1j [35] to test various alternative phylogenetic hypotheses. Based on the ML tree of G1PDH inferred by the RAxML, we divided G1PDHs into 8 groups, *Thermofilum pendens* Hrk-5 (Thermoproteales of Crenarchaeota) (A), Most Thermoproteales (rest of Thermoproteales) (B), Desulfurococcales + Acidilobales + Sulfolobales (C), Thaumarchaeota (D), Euryarchaeota (E), *Bacillus subtilis* subsp. *subtilis* str. 168 (F), Deltaproteobacteria + Haloplasmatales + *Anoxybacillus flavithermus* WK1 + *Bacillus cellulosilyticus* DSM 2522 (G), and Gammaproteobacteria + Actinobacteria (H), together with outgroup (O). Under the two constraint conditions ({{A, F, G, H}, B, C, D, E, O} and {A, B, C, D, E, {F, G, H, O}}), we listed 3,150 relationships among 8 G1PDH groups and 1 outgroup, using ProtML of Molphy 3.2b [36]. Next, the 3,150 relationships were used as the constraint for an ML tree search performed with RAxML with the PROTGAMMALG model. The log-likelihoods of 3,150 resultant trees were compared, and the top 2,000 trees on the log-likelihoods were then used for the AU test with Consel. The species (or groups) with white columns form a group together with the outgroup. Those with red columns form a distinct subgroup within the group including the outgroup (white columns).
