# Supplementary material for: Birth of Archaeal Cells: Molecular Phylogenetic Analyses of G1P Dehydrogenase, G3P Dehydrogenases, and Glycerol Kinase Suggest Derived Features of Archaeal Membranes Having G1P Polar Lipids
Source: Archaea. 2016 Sep 28;2016:1802675. doi: 10.1155/2016/1802675 (PMC5059525; doi:10.1155/2016/1802675)
Supplement: Supplementary file 1 — Supplementary Table S1: The list of sequence entries used to infer the G1PDH (EgsA/AraM) tree. Supplementary Table S2: The list of sequence entries used to infer the G3PDH (GpsA) tree. Supplementary Table S3: The list of sequence entries used to infer the G3PDH (GlpA/D) tree. Supplementary Table S4: The list of sequence entries used to infer the GK (GlpK) tree. Supplementary Table S5: Statistical test showing a maximum likelihood analysis of G1PDH. The AU test [34] was performed using Consel v0.1j [35] to test various alternative phylogenetic hypotheses. Based on the ML tree of G1PDH inferred by the RAxML, we divided G1PDHs into 8 groups, Thermofilum pendens Hrk-5 (Thermoproteales of Crenarchaeota) (A), Most Thermoproteales (rest of Thermoproteales) (B), Desulfurococcales + Acidilobales + Sulfolobales (C), Thaumarchaeota (D), Euryarchaeota (E), Bacillus subtilis subsp. subtilis str. 168 (F), Deltaproteobacteria + Haloplasmatales + Anoxybacillus flavithermus WK1 + Bacillus cellulosilyticus DSM 2522 (G), and Gammaproteobacteria + Actinobacteria (H), together with outgroup (O). Under the two constraint conditions ({{A, F, G, H}, B, C, D, E, O} and {A, B, C, D, E, {F, G, H, O}}), we listed 3,150 relationships among 8 G1PDH groups and 1 outgroup, using ProtML of Molphy 3.2b [36]. Next, the 3,150 relationships were used as the constraint for an ML tree search performed with RAxML with the PROTGAMMALG model. The log-likelihoods of 3,150 resultant trees were compared, and the top 2,000 trees on the log-likelihoods were then used for the AU test with Consel. The species (or groups) with white columns form a group together with the outgroup. Those with red columns form a distinct subgroup within the group including the outgroup (white columns). Supplementary Figure S1: The trimed multiple alignment used for the phylogenetic analyses of G1PDH (EgsA/AraM). Details how to create this alignment is found in section 2.1 of main text. Supplementary Figure S2. Alignment of G1PDH (Egs [file 1802675.f1.zip › Supplementary_Materials_yokobori_et_al_part_1_ARCH_1737186.docx]

**Supplementary Materials**

**When Archaea appeared —Molecular phylogenetic analyses of G1P dehydrogenase, G3P dehydrogenases, and glycerol kinase suggest derived features of archaeal membranes having G1P-polar lipids**

Shin-ichi Yokobori,^1^ Yoshiki Nakajima,^1^ Satoshi Akanuma,^2^ & Akihiko Yamagishi^1^

^1^Laboratory of Extremophiles, Department of Applied Life Sciences, School of Life Sciences, Tokyo University of Pharmacy and Life Sciences, 1432-1 Horinouchi, Hachioji, Tokyo 192-0392, Japan.

^2^Faculty of Human Sciences, Waseda University, 2-579-15 Mikajima, Tokorozawa, Saitama 359-1192, Japan.

Correspondence should be addressed to Akihiko Yamagishi: yamagish@toyaku.ac.jp

Supplementary Table S1. The list of sequence entries used to infer the G1PDH (EgsA/AraM) tree.

| Taxonomy | Organism | Accession |
| --- | --- | --- |
| Archaea; Crenarchaeota; Thermoprotei; Acidilobales; | *Acidilobus saccharovorans* 345-15 | YP_003815753 |
| Archaea; Crenarchaeota; Thermoprotei; Desulfurococcales; | *Aeropyrum pernix* K1 | NP_147296 |
|  | *Desulfurococcus kamchatkensis* 1221n | YP_002428982 |
|  | *Ignicoccus hospitalis* KIN4/I | YP_001435758 |
|  | *Ignisphaera aggregans* DSM 17230 | YP_003858777 |
|  | *Staphylothermus marinus* F1 | YP_001040946 |
|  | *Staphylothermus hellenicus* DSM 12710 | YP_003669513 |
|  | *Hyperthermus butylicus* DSM 5456 | YP_001013732 |
|  | *Pyrolobus fumarii* 1A | YP_004780337 |
| Archaea; Crenarchaeota; Thermoprotei; Sulfolobales; | *Acidianus hospitalis* W1 | YP_004457824 |
|  | *Metallosphaera sedula* DSM 5348 | YP_001192317 |
|  | *Metallosphaera cuprina* Ar-4 | YP_004410588 |
|  | *Metallosphaera yellowstonensis* MK1 | ZP_09705471 |
|  | *Sulfolobus tokodaii* str. 7 | P58460 |
|  | *Sulfolobus solfataricus* P2 | NP_342265 |
|  | *Sulfolobus islandicus* L.S.2.15 | YP_002832130 |
| Archaea; Crenarchaeota; Thermoprotei; Thermoproteales; | *Thermofilum pendens* Hrk 5 | YP_920632 |
|  | *Caldivirga* *maquilingensis* IC-167 | A8MC03 |
|  | *Pyrobaculum* *aerophilum* str. IM2 | NP_559473 |
|  | *Thermoproteus* *uzoniensis* 768-20 | YP_004338788 |
|  | *Thermoproteus* *tenax* Kra 1 | YP_004893223 |
|  | *Vulcanisaeta* *moutnovskia* 768-28 | YP_004244434 |
| Archaea; Euryarchaeota; | *Aciduliprofundum* *boonei* T469 | YP_003482909 |
| Archaea; Euryarchaeota; Archaeoglobi; | *Archaeoglobus* *fulgidus* DSM 4304 | NP_070502 |
|  | *Archaeoglobus* *veneficus* SNP6 | YP_004341260 |
|  | *Ferroglobus* *placidus* DSM 10642 | YP_003436585 |
| Archaea; Euryarchaeota; Halobacteria; | *Haladaptatus* *paucihalophilus* DX253 | ZP_08044423 |
|  | *Halalkalicoccus* *jeotgali* B3 | YP_003737916 |
|  | *Haloarcula* *hispanica* ATCC 33960 | YP_004795531 |
|  | *Halobacterium* sp. NRC-1 | NP_279479 |
|  | *Halobacterium* sp. DL1 | ZP_09029887 |
|  | *Haloferax* *volcanii* DS2 | YP_003534882 |
|  | *Halogeometricum* *borinquense* DSM 11551 | YP_004037399 |
|  | *Halomicrobium* *mukohataei* DSM 12286 | YP_003178951 |
|  | *Halopiger* *xanaduensis* SH-6 | YP_004598244 |
|  | *Haloquadratum* *walsbyi* DSM 16790 | YP_657330 |
|  | *Halorhabdus* *tiamatea* SARL4B | ZP_08559034 |
|  | *Halorubrum* *lacusprofundi* ATCC 49239 | YP_002565327 |
|  | *Haloterrigena* *turkmenica* DSM 5511 | YP_003404729 |
|  | *Natrialba* *magadii* ATCC 43099 | YP_003480657 |
|  | *Natrinema* *pellirubrum* DSM 15624 | ZP_08963262 |
|  | *Natronobacterium* *gregoryi* SP2 | ZP_08968707 |
|  | *Natronomonas* *pharaonis* DSM 2160 | YP_330974 |
| Archaea; Euryarchaeota; Methanobacteria; | *Methanobacterium* *paludis* | YP_004520039 |
|  | *Methanobrevibacter* *ruminantium* M1 | YP_003423698 |
|  | *Methanobrevibacter* *smithii* DSM 2374 | ZP_05976397 |
|  | *Methanosphaera* *stadtmanae* DSM 3091 | YP_448028 |
|  | *Methanothermobacter* *thermautotrophicus* str. Delta H | P72010 |
|  | *Methanothermobacter* *marburgensis* str. Marburg | YP_003849898 |
|  | *Methanothermus* *fervidus* DSM 2088 | YP_004003574 |
| Archaea; Euryarchaeota; Methanococci; | *Methanocaldococcus* *jannaschii* DSM 2661 | NP_247697 |
|  | *Methanocaldococcus* *vulcanius* M7 | YP_003247891 |
|  | *Methanocaldococcus* sp. FS406-22 | YP_003458869 |
|  | *Methanotorris* *formicicus* Mc-S-70 | ZP_09708452 |
|  | *Methanococcus* *maripaludis* S2 | NP_987345 |
|  | *Methanococcus* *maripaludis* C5 | YP_001097980 |
|  | *Methanococcus* *maripaludis* C7 | YP_001330439 |
| Archaea; Euryarchaeota; Methanomicrobia; | *Methanocella* *conradii* HZ254 | AFC98788 |
|  | *Methanocella* *paludicola* SANAE | YP_003355244 |
|  | *Methanocorpusculum* *labreanum* Z | YP_001030742 |
|  | *Methanoculleus* *marisnigri* JR1 | YP_001047953 |
|  | *Methanolacinia* *petrolearia* DSM 11571 | YP_003894977 |
|  | *Methanoplanus* *limicola* DSM 2279 | ZP_09701990 |
|  | *Methanolinea* *tarda* NOBI-1 | ZP_09042444 |
|  | *Methanoregula* *boonei* 6A8 | YP_001405118 |
|  | *Methanosphaerula* *palustris* E1-9c | YP_002466023 |
|  | *Methanospirillum* *hungatei* JF-1 | YP_502604 |
|  | *Methanosaeta* *harundinacea* 6Ac | AET64327 |
|  | *Methanosaeta* *thermophila* PT | YP_842657 |
|  | *Methanosaeta* *concilii* GP6 | YP_004383617 |
|  | *Methanococcoides* *burtonii* DSM 6242 | YP_565717 |
|  | *Methanohalobium* *evestigatum* Z-7303 | YP_003726269 |
|  | *Methanohalophilus* *mahii* DSM 5219 | YP_003542550 |
|  | *Methanosarcina* *acetivorans* C2A | NP_618561 |
|  | *Methanosarcina* *mazei* Go1 | NP_632614 |
|  | *Methanosarcina* *barkeri* str. Fusaro | YP_303855 |
| Archaea; Euryarchaeota; Methanopyri; | *Methanopyrus* *kandleri* AV19 | NP_614513 |
| Archaea; Euryarchaeota; Thermococci; | *Pyrococcus* *horikoshii* OT3 | O59144 |
|  | *Pyrococcus* *abyssi* GE5 | NP_126372 |
|  | *Pyrococcus* *furiosus* DSM 3638 | NP_579111 |
|  | *Thermococcus* *kodakarensis* KOD1 | YP_183202 |
|  | *Thermococcus* *barophilus* MP | YP_004071243 |
|  | *Thermococcus* sp. 4557 | YP_004763636 |
|  | *Thermococcus* *litoralis* DSM 5473 | ZP_09730767 |
| Archaea; Euryarchaeota; Thermoplasmata; | *Ferroplasma* *acidarmanus* fer1 | ZP_05571072 |
|  | *Picrophilus* *torridus* DSM 9790 | YP_023628 |
|  | *Thermoplasma* *volcanium* GSS1 | NP_111746 |
|  | *Thermoplasma* *acidophilum* DSM 1728 | NP_394614 |
| Archaea; Thaumarchaeota; Cenarchaeales; | *Cenarchaeum* *symbiosum* A | A0RXV2 |
| Archaea; Thaumarchaeota; Nitrosopumilales; | *Candidatus* Nitrosoarchaeum limnia SFB1 | ZP_08257665 |
|  | *Candidatus* Nitrosoarchaeum koreensis MY1 | ZP_08668836 |
|  | *Nitrosopumilus* *maritimus* SCM1 | YP_001583063 |
|  | *Candidatus* Nitrosopumilus salaria BD31 | ZP_09605882 |
| Bacteria; Actinobacteria; Actinobacteridae; | *Nocardia* *brasiliensis* ATCC 700358 | ZP_09840922 |
|  | *Frankia* sp. EAN1pec | YP_001510212 |
|  | *Stackebrandtia* *nassauensis* DSM 44728 | YP_003514403 |
|  | *Actinoplanes* sp. SE50/110 | AEV89150 |
|  | *Micromonospora* *aurantiaca* ATCC 27029 | YP_003839261 |
|  | *Micromonospora* sp. ATCC 39149 | ZP_04606494 |
|  | *Salinispora* *arenicola* CNS-205 | YP_001539736 |
|  | *Thermobispora* *bispora* DSM 43833 | YP_003651341 |
|  | *Streptomyces* *venezuelae* ATCC 10712 | CCA59727 |
|  | *Streptomyces* *coelicoflavus* ZG0656 | EHN78548 |
|  | *Streptomyces* *clavuligerus* ATCC 27064 | ZP_06774702 |
|  | *Streptomyces* *viridochromogenes* DSM 40736 | ZP_07307866 |
|  | *Streptomyces* *zinciresistens* K42 | ZP_08803212 |
|  | *Streptomyces* sp. W007 | ZP_09403399 |
|  | *Nocardiopsis* *dassonvillei* subsp. *dassonvillei* DSM 43111 | YP_003681769 |
|  | *Thermomonospora* *curvata* DSM 43183 | YP_003301673 |
| Bacteria; Chlorobi; Chlorobia; | *Chlorobaculum* *parvum* NCIB 8327 | YP_001998671 |
|  | *Chlorobium* *ferrooxidans* DSM 13031 | ZP_01385829 |
| Bacteria; Chrysiogenetes; Chrysiogenales; | *Desulfurispirillum* *indicum* S5 | YP_004111517 |
| Bacteria; Cyanobacteria; Nostocales; | *Anabaena* *variabilis* ATCC 29413 | YP_320537 |
|  | *Cylindrospermopsis* *raciborskii* CS-505 | ZP_06307585 |
|  | *Nodularia* *spumigena* CCY9414 | ZP_01631542 |
|  | *Nostoc* *punctiforme* PCC 73102 | YP_001868372 |
|  | *Raphidiopsis* *brookii* D9 | ZP_06306057 |
|  | 'Nostoc azollae' 0708 | YP_003722140 |
| Bacteria; Cyanobacteria; Oscillatoriales; | *Arthrospira* *platensis* str. Paraca | ZP_06380832 |
| Bacteria; Cyanobacteria; Oscillatoriophycideae; | *Acaryochloris* *marina* MBIC11017 | YP_001517882 |
|  | *Crocosphaera* *watsonii* WH 8501 | ZP_00515762 |
|  | *Cyanothece* sp. ATCC 51142 | YP_001802726 |
|  | *Cyanothece* sp. PCC 7822 | YP_003885570 |
|  | *Microcystis* *aeruginosa* NIES-843 | YP_001657591 |
|  | *Synechococcus* *elongatus* PCC 7942 | YP_400670 |
|  | *Synechococcus* sp. JA-2-3B'a(2-13) | YP_476598 |
|  | *Synechococcus* sp. CC9311 | YP_729867 |
|  | *Thermosynechococcus* *elongatus* BP-1 | NP_682841 |
|  | *Arthrospira* *platensis* NIES-39 | BAI91054 |
|  | *Arthrospira* *maxima* CS-328 | ZP_03274582 |
|  | *Coleofasciculus* *chthonoplastes* PCC 7420 | ZP_05024185 |
|  | *Lyngbya* sp. PCC 8106 | ZP_01620809 |
|  | *Microcoleus* *vaginatus* FGP-2 | ZP_08491102 |
|  | *Trichodesmium* *erythraeum* IMS101 | YP_720871 |
| Bacteria; Cyanobacteria; Prochlorales; | *Prochlorococcus* *marinus* str. MIT 9515 | YP_001011492 |
| Bacteria; Deinococcus-Thermus; Deinococci; | *Truepera* *radiovictrix* DSM 17093 | YP_003704889 |
| Bacteria; Firmicutes; Bacilli; | *Anoxybacillus* flavithermus *WK1* | YP_002315903 |
|  | *Bacillus* *subtilis* subsp. *subtilis* str. 168 | NP_390754 |
|  | *Bacillus* *cellulosilyticus* DSM 2522 | YP_004095225 |
|  | *Listeria* *monocytogenes* FSL J1-208 | EHY62723 |
|  | *Enterococcus* *italicus* DSM 15952 | ZP_07895093 |
|  | *Enterococcus* *casseliflavus* ATCC 12755 | ZP_08144556 |
|  | *Leuconostoc* *gelidum* KCTC 3527 | ZP_08479472 |
|  | *Leuconostoc* *citreum* KM20 | YP_001728665 |
| Bacteria; Firmicutes; Clostridia; | *Clostridium* sp. DL-VIII | ZP_09206713 |
|  | *Eubacterium* *rectale* ATCC 33656 | YP_002938300 |
|  | *Clostridium* *lentocellum* DSM 5427 | YP_004309602 |
|  | *Roseburia* *intestinalis* L1-82 | ZP_04742161 |
|  | *Desulfotomaculum* *nigrificans* DSM 574 | ZP_08114177 |
|  | *Acetivibrio* *cellulolyticus* CD2 | ZP_09462476 |
|  | *Moorella* *thermoacetica* ATCC 39073 | YP_429338 |
| Bacteria; Fusobacteria; Fusobacteriales; | *Fusobacterium* *ulcerans* ATCC 49185 | ZP_07929431 |
| Bacteria; Haloplasmatales; Haloplasmataceae; | *Haloplasma* *contractile* SSD-17B | ZP_08558101 |
| Bacteria; Planctomycetes; Planctomycetia; | *Planctomyces* *limnophilus* DSM 3776 | YP_003629610 |
| Bacteria; Proteobacteria; Alphaproteobacteria; | *Bradyrhizobiaceae* *bacterium* SG-6C | ZP_08628955 |
|  | *Methylobacterium* *nodulans* ORS 2060 | YP_002500804 |
|  | *Methylocystis* sp. ATCC 49242 | ZP_08074163 |
|  | *Sinorhizobium* *meliloti* 1021 | NP_386656 |
|  | *Sinorhizobium* *medicae* WSM419 | YP_001328127 |
|  | *Xanthobacter* *autotrophicus* Py2 | YP_001416616 |
|  | *Paracoccus* sp. TRP | ZP_08664313 |
|  | *Roseomonas* *cervicalis* ATCC 49957 | ZP_06895512 |
|  | Acetobacteraceae bacterium AT-5844 | ZP_09398437 |
| Bacteria; Proteobacteria; Deltaproteobacteria; | *Desulfarculus* *baarsii* DSM 2075 | YP_003806763 |
|  | *Desulfatibacillum* *alkenivorans* AK-01 | YP_002430034 |
|  | *Desulfovibrio* *vulgaris* DP4 | YP_961214 |
|  | *Desulfovibrio* *aespoeensis* Aspo-2 | YP_004122072 |
|  | *Desulfovibrio* *fructosovorans* JJ | ZP_07334616 |
|  | *Desulfovibrio* sp. FW1012B | ZP_09131560 |
|  | *Geobacter* *lovleyi* SZ | YP_001950958 |
|  | *Sorangium* *cellulosum* So ce56 | YP_001616037 |
|  | *Syntrophobacter* *fumaroxidans* MPOB | YP_844654 |
| Bacteria; Proteobacteria; Gammaproteobacteria; | *Pseudoalteromonas* *tunicata* D2 | ZP_01134701 |
|  | *Shewanella* *violacea* DSS12 | YP_003554854 |
|  | *Shewanella* *benthica* KT99 | ZP_02156139 |
|  | *Escherichia* *coli* str. K-12 substr. MG1655 | AAC76927 |
|  | *Pseudomonas* *putida* W619 | YP_001748564 |
| Bacteria; Spirochaetes; Spirochaetales; | *Brachyspira* *intermedia* PWS/A | AEM22479 |
| Bacteria; Thermotogae; Thermotogales; | *Kosmotoga* *olearia* TBF 19.5.1 | YP_002940231 |
|  | *Thermotoga* *maritima* | 1KQ3_A |
|  | *Thermotoga* *petrophila* RKU-1 | YP_001244096 |
|  | *Thermotoga* *neapolitana* DSM 4359 | YP_002533789 |

Supplementary Table S2. The list of sequence entries used to infer the G3PDH (GpsA) tree.

| Taxonomy | Organism | Accession |
| --- | --- | --- |
| Archaea; Crenarchaeota; Thermoprotei; Acidilobales; | *Acidilobus saccharovorans* | WP_013266948 |
|  | *Caldisphaera lagunensis* | WP_015231820 |
| Archaea; Crenarchaeota; Thermoprotei; Desulfurococcales; | *Aeropyrum pernix* | WP_010866401 |
|  | *Pyrolobus fumarii* | WP_014026963 |
| Archaea; Crenarchaeota; Thermoprotei; Sulfolobales; | *Sulfolobus tokodaii* | WP_010978008 |
|  | *Sulfolobus solfataricus* | WP_010923104 |
| Archaea; Crenarchaeota; Thermoprotei; Thermoproteales; | *Thermofilum pendens* | WP_011753379 |
|  | *Thermoproteus tenax* | WP_014126928 |
| Archaea; Diapherotrites; | *Candidatus* Iainarchaeum andersonii | WP_041910265 |
| Archaea; Euryarchaeota; Archaeoglobi; | *Archaeoglobus sulfaticallidus* | WP_015591014 |
|  | *Archaeoglobus fulgidus* DSM 8774 | AIG96861 |
|  |  | AIG97998 |
|  | *Archaeoglobus fulgidus* | WP_010878100 |
|  | *Ferroglobus placidus* | WP_012965915 |
| Archaea; Euryarchaeota; Halobacteria; | *Halobacterium salinarum* | WP_010902952 |
|  |  | WP_010901982 |
| Archaea; Euryarchaeota; Methanobacteria; | *Methanobacterium formicicum* DSM 3637 | EKF86026 |
|  | *Methanobrevibacter ruminantium* | WP_012956986 |
|  | *Methanothermobacter thermautotrophicus* CaT2 | BAM69559 |
| Archaea; Euryarchaeota; Methanococci; | *Methanocaldococcus jannaschii* DSM 2661 | Q57871 |
| Archaea; Euryarchaeota; Methanomicrobia; | *Methanocella paludicola* SANAE | BAI62488 |
|  | *Methanoculleus* *marisnigri* | WP_011844442 |
|  | *Methanoculleus* *marisnigri* JR1 | ABN56632 |
|  | *Methanosarcina mazei* | WP_015411604 |
| Archaea; Euryarchaeota; Thermococci; | *Pyrococcus furiosus* | WP_011012501 |
| Archaea; Euryarchaeota; Thermoplasmata; | *Methanomassiliicoccus luminyensis* | WP_026068756 |
|  | *Thermoplasma acidophilum* | WP_010900720 |
| Archaea; Korarchaeota; | *Candidatus* Korarchaeum cryptofilum | WP_012308771 |
| Bacteria; Acetothermia; | *Candidatus* Acetothermus autotrophicum | BAL59589 |
| Bacteria; Acidobacteria; Acidobacteriales; | *Acidobacterium* *capsulatum* | WP_015898440 |
|  |  | WP_015895922 |
|  | *Candidatus* Koribacter versatilis | WP_011522939 |
|  | *Granulicella tundricola* | WP_013579418 |
|  | *Terriglobus roseus* | WP_014784837 |
| Bacteria; Acidobacteria; Acidobacteria subdivision 4; | *Candidatus* Chloracidobacterium thermophilum | WP_014100138 |
| Bacteria; Acidobacteria; Acidobacteria subdivision 23; | *Thermoanaerobaculum aquaticum* | KDA54421 |
| Bacteria; Acidobacteria; Holophagae; | *Geothrix fermentans* | WP_026853830 |
|  | *Holophaga foetida* | WP_005034894 |
| Bacteria; Acidobacteria; Solibacteres; | *Candidatus* Solibacter usitatus | WP_011682101 |
|  |  | WP_011687232 |
|  |  | WP_041858319 |
|  | *Candidatus* Solibacter usitatus Ellin6076 | ABJ87618 |
|  |  | ABJ88405 |
|  | *Bryobacter aggregatus* | WP_031500416 |
| Bacteria; Actinobacteria; Actinobacteridae; | *Actinomyces* *turicensis* ACS-279-V-Col4 | EJZ85434 |
|  |  | EJZ86488 |
|  | *Actinomyces vaccimaxillae* | WP_022868963 |
|  |  | WP_026459441 |
|  | *Actinomyces slackii* | WP_026427628 |
|  | *Corynebacterium variabile* | WP_014010264 |
|  |  | WP_030146704 |
|  | *Corynebacterium efficiens* | WP_006769599 |
|  | *Microbacterium indicum* | WP_029150047 |
|  | *Propionibacterium propionicum* | WP_014845387 |
|  |  | WP_014847302 |
|  | *Streptomyces* *sviceus* ATCC 29083 | EDY53897 |
|  |  | EDY55566 |
| Bacteria; Actinobacteria; Coriobacteridae; | *Eggerthella* sp. YY7918 | BAK44800 |
| Bacteria; Actinobacteria; Rubrobacteridae; | *Rubrobacter xylanophilus* | WP_011565017 |
|  | *Rubrobacter radiotolerans* | AHY48316 |
| Bacteria; Aerophobetes | Aerophobetes bacterium SCGC AAA255-F10 | WP_029962257 |
| Bacteria; Aerophobetes; | *Candidatus* Aerophobus profundus | WP_041891889 |
| Bacteria; Aminicenantes; | *Candidatus* Aminicenans sakinawicola | WP_020260390 |
| Bacteria; Aquificae; Aquificales; | *Aquifex* *aeolicus* VF5 | O67555 |
|  | *Aquifex aeolicus* | WP_010879940 |
| Bacteria; Aquificae; Desulfurobacteriales; | *Desulfurobacterium thermolithotrophum* | WP_013638206 |
|  | *Thermovibrio* *ammonificans* HB-1 | ADU96654 |
| Bacteria; Armatimonadetes; Chthonomonadetes; | *Chthonomonas* *calidirosea* T49 | CCW34705 |
| Bacteria; Armatimonadetes; | *Fimbriimonas* *ginsengisoli* Gsoil 348 | AIE83379 |
|  | *Fimbriimonas* *ginsengisoli* | WP_025229365 |
| Bacteria; Atribacteria | Atribacteria bacterium SCGC AAA255-G05 | WP_029717092 |
| Bacteria; Bacteroidetes; Bacteroidia; | *Bacteroides* *fluxus* YIT 12057 | EGF55486 |
|  | *Draconibacterium orientale* | AHW61232 |
| Bacteria; Bacteroidetes; Cytophagia; | *Cyclobacterium marinum* | WP_014018215 |
|  |  | WP_014022044 |
|  | *Cytophaga aurantiaca* | WP_018342739 |
|  | *Cytophaga hutchinsonii* | WP_011586931 |
| Bacteria; Bacteroidetes; Flavobacteriia; | *Fluviicola taffensis* | WP_013687203 |
|  |  | WP_013686815 |
|  | *Flavobacterium* *psychrophilum* FPG3 | AIN73080 |
|  | *Flavobacterium* *aquatile* LMG 4008 | KGD68544 |
| Bacteria; Bacteroidetes; Sphingobacteriia; | *Chitinophaga pinensis* | WP_012794626 |
|  | *Saprospira grandis* | WP_015692095 |
|  | *Sphingobacterium* *paucimobilis* HER1398 | ERJ58061 |
| Bacteria; Caldiserica | Caldiserica bacterium JGI 0000059-M03 | WP_041907939 |
| Bacteria; Caldithrix | *Caldithrix* *abyssi* DSM 13497 | EHO42813 |
|  | *Caldithrix* *abyssi* | WP_006930620 |
| Bacteria; Calescamantes; | *Candidatus* Calescibacterium nevadense | WP_022804334 |
|  |  | WP_029665305 |
| Bacteria; candidate division NC10; | *Candidatus* Methylomirabilis oxyfera | CBE68583 |
| Bacteria; Chlamydiae; Chlamydiales; | *Chlamydia trachomatis* A/HAR-13 | AAX50992 |
|  | *Chlamydophila* *pneumoniae* AR39 | AAF38792 |
|  | *Chlamydia* sp. 'Rubis' | CDZ80591 |
|  | *Parachlamydia* *acanthamoebae* | KIA78740 |
|  | *Simkania* *negevensis* Z | CCB88100 |
|  | *Waddlia* *chondrophila* | WP_013182529 |
|  | *Waddlia* *chondrophila* WSU 86-1044 | ADI39161 |
| Bacteria; Chlorobi; Chlorobia; | *Chlorobium* *ferrooxidans* DSM 13031 | EAT59593 |
|  | *Chlorobium limicola* | WP_012466345 |
|  | *Prosthecochloris aestuarii* | WP_012506707 |
| Bacteria; Chloroflexi; Anaerolineae; | *Anaerolinea* *thermophila* UNI-1 | BAJ64751 |
| Bacteria; Chloroflexi; Caldilineae; | *Caldilinea* *aerophila* DSM 14535 = NBRC 104270 | BAM00728 |
|  | *Caldilinea aerophila* | WP_014432352 |
| Bacteria; Chloroflexi; Chloroflexia; | *Chloroflexus aggregans* | WP_012615659 |
|  |  | WP_012616384 |
|  | *Herpetosiphon aurantiacus* | WP_012191918 |
|  |  | WP_012191410 |
| Bacteria; Chloroflexi; Dehalococcoidia | Dehalococcoidia bacterium SCGC AB-539-J10 | WP_029475349 |
| Bacteria; Chloroflexi; Dehalococcoidia; | *Dehalococcoides* *mccartyi* CBDB1 | CAI83405 |
| Bacteria; Chloroflexi; Ktedonobacteria; | *Ktedonobacter racemifer* | WP_007908052 |
| Bacteria; Chloroflexi; Sphaerobacteridae; | *Sphaerobacter thermophilus* | WP_012873366 |
| Bacteria; Chrysiogenetes; Chrysiogenales; | *Chrysiogenes arsenatis* | WP_027389158 |
|  |  | WP_027389174 |
|  | *Desulfurispirillum indicum* | WP_013506429 |
|  |  | WP_013506025 |
| Bacteria; Cloacimonetes; | *Candidatus* Cloacimonas acidaminovorans str. Evry | CAO80747 |
| Bacteria; Cloacimonetes; | *Candidatus* Cloacimonas acidaminovorans | WP_015425177 |
| Bacteria; Cyanobacteria; Gloeobacteria; | *Gloeobacter* *kilaueensis* JS1 | AGY57217 |
|  | *Gloeobacter* *kilaueensis* | WP_023173759 |
| Bacteria; Cyanobacteria; Nostocales; | *Hassallia* *byssoidea* VB512170 | KIF34618 |
|  | *Scytonema* *hofmanni* | WP_033336837 |
| Bacteria; Cyanobacteria; Oscillatoriophycideae; | *Crocosphaera* *watsonii* WH 8501 | EAM47652 |
|  | *Microcystis* *aeruginosa* PCC 7806 | CAO88267 |
|  | *Synechococcus* *elongatus* PCC 7942 | Q935Z2 |
|  | *Arthrospira* *platensis* NIES-39 | BAI93051 |
|  | *Nodosilinea* *nodulosa* | WP_026073203 |
| Bacteria; Cyanobacteria; Pleurocapsales; | *Chroococcidiopsis* *thermalis* | WP_015154928 |
|  | *Xenococcus* sp. PCC 7305 | WP_040897270 |
| Bacteria; Cyanobacteria; Stigonematales; | *Fischerella muscicola* | WP_016868808 |
|  |  | WP_016868299 |
|  | *Mastigocoleus testarum* | WP_027837927 |
|  |  | WP_027840549 |
| Bacteria; Deferribacteres; Deferribacterales; | *Deferribacter desulfuricans* | WP_013008665 |
|  |  | WP_013008173 |
|  | *Deferribacter* *desulfuricans* SSM1 | BAI79627 |
|  | *Mucispirillum* *schaedleri* ASF457 | ESJ98052 |
| Bacteria; Deinococcus-Thermus; Deinococci; | *Deinococcus* *radiodurans* R1 | Q9RR76 |
|  | *Deinococcus radiodurans* | WP_010889102 |
|  | *Deinococcus geothermalis* | WP_011525920 |
|  | *Thermus thermophilus* | WP_014629693 |
|  |  | WP_014510291 |
|  | *Thermus* *thermophilus* HB8 | Q5SHJ0 |
| Bacteria; Dictyoglomi; Dictyoglomales; | *Dictyoglomus* *thermophilum* | WP_012548792 |
| Bacteria; Elusimicrobia; Elusimicrobia; | *Elusimicrobium* *minutum* | WP_012415277 |
| Bacteria; Fibrobacteres; Chitinivibrionia; | *Chitinivibrio* *alkaliphilus* ACht1 | ERP31319 |
| Bacteria; Fibrobacteres; Fibrobacterales; | *Fibrobacter* *succinogenes* | WP_014546887 |
|  |  | WP_014545700 |
| Bacteria; Firmicutes; Bacilli; | *Bacillus* *halodurans* C-125 | BAB05359 |
|  | *Bacillus* *subtilis* subsp. *subtilis* str. 168 | AAA86746 |
|  | *Bacillus* *subtilis* subsp. *subtilis* | AJE96931 |
|  | *Bacillus* *subtilis* subsp. *subtilis* str. SC-8 | EHA32395 |
|  | *Virgibacillus halodenitrificans* | CDQ35629 |
|  | *Brevibacillus massiliensis* | WP_019122471 |
|  |  | WP_019123658 |
|  | *Paenibacillus* *curdlanolyticus* YK9 | EFM09788 |
|  | *Staphylococcus* *aureus* A9635 | EEV71258 |
|  | *Aerococcus viridans* | WP_003143054 |
|  | *Enterococcus faecalis* | WP_016627279 |
|  | *Lactobacillus* *casei* 32G | EKQ01318 |
|  | *Oenococcus* *kitaharae* DSM 17330 | EHN59105 |
| Bacteria; Firmicutes; Clostridia; | *Clostridium* *ultunense* Esp | CCQ96024 |
|  | *Symbiobacterium* *thermophilum* IAM 14863 | BAD40666 |
|  | *Anaerofustis* *stercorihominis* DSM 17244 | EDS71456 |
|  |  | EDS71629 |
|  |  | EDS72882 |
|  | *Eubacterium limosum* | WP_013382372 |
|  | *Heliobacterium modesticaldum* | WP_012281247 |
|  | *Anaerostipes* *caccae* DSM 14662 | EDR96723 |
|  | *Desulfotomaculum alcoholivorax* | WP_027365188 |
|  | *Ruminococcus flavefaciens* | WP_009984763 |
|  | *Thermoanaerobacter wiegelii* | WP_041589314 |
|  | *Thermodesulfobium narugense* | WP_013755507 |
| Bacteria; Firmicutes; Erysipelotrichia; | *Erysipelothrix* *rhusiopathiae* ATCC 19414 | EFY09694 |
|  | *Turicibacter* *sanguinis* PC909 | EFF64393 |
| Bacteria; Firmicutes; Negativicutes; | *Acidaminococcus intestini* | WP_026385421 |
|  | *Dialister invisus* | WP_007069844 |
|  | *Pelosinus* *fermentans* DSM 17108 | EIW28650 |
|  |  | EIW33496 |
|  | *Veillonella* *atypica* KON | EKY20870 |
| Bacteria; Fusobacteria; Fusobacteriales; | *Fusobacterium perfoetens* | WP_027129016 |
|  | *Fusobacterium ulcerans* | WP_005979179 |
|  | *Fusobacterium* *nucleatum* CTI-3 | ERT36540 |
|  | *Leptotrichia* *wadei* F0279 | ERK54075 |
| Bacteria; Gemmatimonadetes; Gemmatimonadales; | *Gemmatimonas* *aurantiaca* T-27 | BAH38645 |
|  |  | BAH39749 |
|  | *Gemmatimonas aurantiaca* | WP_015894566 |
| Bacteria; Haloplasmatales; Haloplasmataceae; | *Haloplasma contractile* | WP_008825790 |
|  |  | WP_008824753 |
| Bacteria; Ignavibacteriae; Ignavibacteria; | *Ignavibacterium album* | WP_014561933 |
|  |  | WP_014560269 |
|  | *Melioribacter roseus* | WP_014856496 |
| Bacteria; Latescibacteria; | *Candidatus* Latescibacter anaerobius | WP_022817110 |
| Bacteria; Lentisphaerae; Lentisphaeria; | *Lentisphaera* *araneosa* HTCC2155 | EDM28342 |
| Bacteria; Marinimicrobia | Marinimicrobia bacterium SCGC AAA298-D23 | WP_022839043 |
| Bacteria; Microgenomates | Microgenomates bacterium SCGC AAA011-L6 | WP_029251330 |
| Bacteria; Nitrospinae; Nitrospinia; | *Nitrospina* *gracilis* 3/211 | CCQ91501 |
| Bacteria; Nitrospirae; Nitrospirales; | *Leptospirillum* *ferrodiazotrophum* | EES51880 |
|  | *Candidatus* Nitrospira defluvii | CBK40306 |
|  | *Thermodesulfovibrio* *thiophilus* | WP_028844402 |
| Bacteria; Omnitrophica | *Omnitrophica* *bacterium* SCGC AAA257-O07 | WP_028483971 |
| Bacteria; Parcubacteria; | *Candidatus* Paceibacter normanii | WP_027927187 |
| Bacteria; Planctomycetes; Phycisphaerae; | *Phycisphaera* *mikurensis* NBRC 102666 | BAM02304 |
| Bacteria; Planctomycetes; Planctomycetia; | *Candidatus* Kuenenia stuttgartiensis | CAJ72443 |
|  | *Candidatus* Scalindua brodae | KHE91036 |
|  | *Planctomyces limnophilus* | WP_013109618 |
|  | *Planctomyces brasiliensis* | WP_013629089 |
|  | *Zavarzinella formosa* | WP_020472803 |
| Bacteria; Poribacteria | *Candidatus* Poribacteria sp. WGA-4E | WP_020382503 |
|  | Poribacteria bacterium WGA-3G | WP_022814938 |
|  | *Candidatus* Poribacteria sp. WGA-4C | WP_041883857 |
| Bacteria; Proteobacteria; Alphaproteobacteria; | *Caulobacter vibrioides* | WP_010920237 |
|  | *Geminicoccus roseus* | WP_035486379 |
|  | *Magnetococcus marinus* | WP_011712422 |
|  |  | WP_011712029 |
|  | *Micavibrio aeruginosavorus* | WP_014103908 |
|  | *Bartonella tamiae* | WP_008040187 |
|  | *Mesorhizobium* *loti* MAFF303099 | BAB50933 |
|  | *Rhizobium* *gallicum* | WP_018444522 |
|  | *Sinorhizobium* *medicae* WSM419 | ABR61522 |
|  |  | ABR61793 |
|  | *Dinoroseobacter* *shibae* | WP_012180071 |
|  |  | WP_012187223 |
|  | *Acetobacter* *aceti* 1023 | KDE20742 |
|  | *Rickettsia* *prowazekii* str. Madrid E | NP_220931 |
|  |  | CAA14899 |
|  | *Rickettsia* *prowazekii* str. GvF12 | EOB10299 |
|  | *Sneathiella glossodoripedis* | WP_025897876 |
|  | *Erythrobacter litoralis* | WP_011414753 |
| Bacteria; Proteobacteria; Betaproteobacteria; | *Burkholderia* *rhizoxinica* HKI 454 | CBW76174 |
|  | *Burkholderia* *terrae* BS001 | EIN03175 |
|  | *Comamonas composti* | WP_027014774 |
|  | *Methylibium petroleiphilum* | WP_011828317 |
|  | *Oxalobacter formigenes* | WP_005878220 |
|  | *Rubrivivax* *gelatinosus* IL144 | BAL97527 |
|  | *Ferrovum* *myxofaciens* | WP_031597515 |
|  | *Tepidiphilus* *margaritifer* | WP_028874256 |
|  | *Methylophilus* *methylotrophus* | WP_018986066 |
|  | *Leeia* *oryzae* | WP_018149894 |
|  | *Neisseria* *shayeganii* | WP_009119946 |
|  | *Neisseria* *bacilliformis* ATCC BAA-1200 | EGF07155 |
|  | *Nitrosomonas* *eutropha* | WP_041353699 |
|  | *Nitrosomonas* *europaea* ATCC 19718 | CAD86120 |
|  | *Azospira* *oryzae* | WP_014235438 |
|  | *Sulfuricella* *denitrificans* | WP_009207553 |
| Bacteria; Proteobacteria; Deltaproteobacteria; | *Bacteriovorax* *marinus* SJ | CBW27318 |
|  | *Bacteriovorax marinus* | WP_014245495 |
|  | *Desulfarculus baarsii* | WP_013258669 |
|  | *Desulfatibacillum aliphaticivorans* | WP_028314015 |
|  | *Desulfatibacillum alkenivorans* | WP_015948404 |
|  | *Desulfobacter postgatei* | WP_004074082 |
|  | *Desulfohalobium retbaense* | WP_015752839 |
|  | *Desulfonatronum lacustre* | WP_028573333 |
|  | *Desulfurella* *acetivorans* A63 | AHF96513 |
|  | *Geobacter bemidjiensis* | WP_012528449 |
|  | *Geobacter metallireducens* | WP_004511638 |
|  | *Anaeromyxobacter dehalogenans* | WP_011419455 |
|  | *Myxococcus xanthus* | WP_011551471 |
|  | *Haliangium ochraceum* | WP_012829147 |
|  | *Plesiocystis* *pacifica* SIR-1 | EDM76201 |
|  | *Sorangium* *cellulosum* | WP_012235425 |
|  | *Syntrophus* *aciditrophicus* SB | ABC77734 |
|  | *Syntrophobacter* *fumaroxidans* | WP_011699536 |
| Bacteria; Proteobacteria; Epsilonproteobacteria; | *Campylobacter* *gracilis* RM3268 | EEV18921 |
|  | *Helicobacter* *hepaticus* ATCC 51449 | AAP77391 |
|  | *Helicobacter sanguini* | KGI52166 |
|  | *Nitratifractor salsuginis* | WP_013553671 |
|  | *Nautilia profundicola* | WP_012663917 |
| Bacteria; Proteobacteria; Gammaproteobacteria; | *Acidithiobacillus ferrooxidans* | WP_012607014 |
|  | *Moritella marina* | WP_019440661 |
|  | *Candidatus* Contendobacter odensis | WP_034430663 |
|  | *Escherichia* *coli* str. K-12 substr. MG1655 | AAB18585 |
|  | *Coxiella* *burnetii* 'MSU Goat Q177' | EAX32839 |
|  | *Methylosarcina* *fibrata* | WP_020565869 |
|  | *Pseudomonas brassicacearum* | AHL34067 |
|  | *Sedimenticola selenatireducens* | WP_029133066 |
|  | *Vibrio* *cholerae* HE48 | EGR06661 |
|  | *Solimonas* *variicoloris* | WP_026353294 |
|  | *Xanthomonas* *translucens* DAR61454 | ELQ11830 |
| Bacteria; Proteobacteria; Zetaproteobacteria; | *Mariprofundus* *ferrooxydans* PV-1 | EAU53684 |
|  | *Mariprofundus* *ferrooxydans* | WP_009849046 |
| Bacteria; Spirochaetes; Spirochaetales; | *Brachyspira* *pilosicoli* WesB | CCG57016 |
|  | *Brachyspira* *hampsonii* 30446 | EKV56906 |
|  | *Leptospira* *interrogans* | WP_001206941 |
|  | *Leptospira* *weilii* str. LNT 1234 | EMN44318 |
|  | *Borrelia* *afzelii* ACA-1 | EEC20646 |
|  | *Sphaerochaeta* *globosa* str. Buddy | ADY12403 |
|  | *Spirochaeta thermophila* | WP_013312828 |
|  | *Treponema caldaria* | WP_013967967 |
| Bacteria; Synergistetes; Synergistia; | *Aminobacterium colombiense* | WP_013049001 |
|  | *Dethiosulfovibrio* *peptidovorans* DSM 11002 | EFC91618 |
|  | *Synergistes* *jonesii* | KEJ91636 |
| Bacteria; Tenericutes; Mollicutes; | *Acholeplasma* *palmae* J233 | CCV64306 |
|  | *Spiroplasma* *mirum* ATCC 29335 | AHI57779 |
|  | *Mycoplasma* *hominis* ATCC 23114 | CAX37303 |
| Bacteria; Thermobaculum | *Thermobaculum* *terrenum* | WP_012875020 |
|  |  | WP_012874650 |
| Bacteria; Thermodesulfobacteria; Thermodesulfobacteriales; | *Thermodesulfatator atlanticus* | WP_022854186 |
|  | *Thermodesulfobacterium geofontis* | WP_013909404 |
|  | *Thermodesulfobacterium thermophilum* | WP_022854988 |
| Bacteria; Thermotogae; Thermotogales; | *Mesoaciditoga lauensis* | WP_036226255 |
|  | *Marinitoga piezophila* | WP_014296334 |
|  | *Thermotoga* *maritima* MSB8 | 1Z82_B |
| Bacteria; Verrucomicrobia; Opitutae; | *Coraliomargarita* *akajimensis* | WP_013043984 |
| Bacteria; Verrucomicrobia; Verrucomicrobiae; | *Verrucomicrobium* *spinosum* | WP_029190313 |
|  | *Pedosphaera* *parvula* Ellin514 | EEF57665 |

Supplementary Table S3. The list of sequence entries used to infer the G3PDH (GlpA/D) tree.

| Taxonomy | Organism | Accession |
| --- | --- | --- |
| Archaea | halophilic archaeon DL31 | YP_004807559 |
| Archaea; Crenarchaeota; Thermoprotei; Desulfurococcales; | *Aeropyrum pernix* K1 | NP_147138 |
|  | *Desulfurococcus* *kamchatkensis* 1221n | YP_002428574 |
|  | *Desulfurococcus* *fermentans* DSM 16532 | YP_006402373 |
|  | *Staphylothermus* *marinus* F1 | YP_001040279 |
|  | *Staphylothermus* *hellenicus* DSM 12710 | YP_003668585 |
| Archaea; Crenarchaeota; Thermoprotei; Sulfolobales; | *Metallosphaera* *sedula* DSM 5348 | YP_001191262 |
|  | *Sulfolobus* *solfataricus* P2 | NP_343866 |
|  | *Sulfolobus* *acidocaldarius* DSM 639 | YP_255763 |
|  |  | YP_256621 |
|  | *Sulfolobus* *islandicus* L.S.2.15 | YP_002831137 |
|  | *Sulfolobus* *islandicus* L.D.8.5 | YP_003418485 |
| Archaea; Crenarchaeota; Thermoprotei; Thermoproteales; | *Thermofilum* *pendens* Hrk 5 | YP_920528 |
|  |  | YP_920775 |
|  | *Caldivirga* *maquilingensis* IC-167 | YP_001541610 |
|  | *Pyrobaculum* *oguniense* TE7 | YP_005260642 |
|  | *Vulcanisaeta* *distributa* DSM 14429 | YP_003900729 |
|  |  | YP_003901260 |
|  |  | YP_003901840 |
|  |  | YP_003901936 |
|  | *Vulcanisaeta* *moutnovskia* 768-28 | YP_004243833 |
|  |  | YP_004245070 |
|  |  | YP_004245910 |
| Archaea; Euryarchaeota; Aciduliprofundum | *Aciduliprofundum* *boonei* T469 | YP_003483900 |
| Archaea; Euryarchaeota; Archaeoglobi; | *Archaeoglobus* *fulgidus* DSM 4304 | NP_070157 |
|  | *Archaeoglobus* *veneficus* SNP6 | YP_004342538 |
| Archaea; Euryarchaeota; Halobacteria; Halobacteriales; | *Halalkalicoccus* *jeotgali* B3 | YP_003735329 |
|  |  | YP_003737306 |
|  | *Haloarcula* *marismortui* ATCC 43049 | AAV46823 |
|  |  | YP_135276 |
|  | *Haloarcula* *hispanica* ATCC 33960 | YP_004795864 |
|  |  | YP_004797041 |
|  | *Halobacterium* sp. NRC-1 | NP_444207 |
|  | *Halobacterium* *salinarum* R1 | YP_001689097 |
|  |  | YP_001689788 |
|  | *Haloferax* *volcanii* DS2 | YP_003533725 |
|  |  | YP_003535585 |
|  | *Haloferax* *mediterranei* ATCC 33500 | YP_006349292 |
|  | *Halogeometricum* *borinquense* DSM 11551 | YP_004036697 |
|  |  | YP_004036704 |
|  | *Halomicrobium* *mukohataei* DSM 12286 | YP_003178331 |
|  |  | YP_003178385 |
|  | *Halopiger* *xanaduensis* SH-6 | YP_004596807 |
|  |  | YP_004598408 |
|  | *Haloquadratum* *walsbyi* DSM 16790 | YP_657500 |
|  |  | YP_658392 |
|  | *Haloquadratum* *walsbyi* C23 | YP_005840447 |
|  | *Halorhabdus* *utahensis* DSM 12940 | YP_003129602 |
|  |  | YP_003130380 |
|  | *Halorubrum* *lacusprofundi* ATCC 49239 | YP_002565787 |
|  | *Haloterrigena* *turkmenica* DSM 5511 | YP_003404503 |
|  |  | YP_003406508 |
|  | *Natrialba* *magadii* ATCC 43099 | YP_003479079 |
|  |  | YP_003481190 |
|  | *Natrinema* sp. J7-2 | YP_006540773 |
|  |  | YP_006541035 |
| Archaea; Euryarchaeota; Methanomicrobia; Methanocellales; | *Methanocella* *arvoryzae* MRE50 | YP_687586 |
|  | *Methanocella* *paludicola* SANAE | YP_003356194 |
|  | *Methanocella* *conradii* HZ254 | YP_005380331 |
| Archaea; Euryarchaeota; Thermococci; Thermococcales; | *Pyrococcus* *abyssi* GE5 | NP_125962 |
|  | *Pyrococcus* *furiosus* DSM 3638 | NP_579734 |
|  | *Pyrococcus* *yayanosii* CH1 | YP_004624760 |
|  | *Pyrococcus* sp. ST04 | YP_006353723 |
|  | *Thermococcus* *kodakarensis* KOD1 | YP_183806 |
|  | *Thermococcus* *onnurineus* NA1 | YP_002306585 |
|  | *Thermococcus* sp. AM4 | YP_002582268 |
|  | *Thermococcus* *gammatolerans* | YP_002959389 |
|  |  | YP_002960157 |
|  | *Thermococcus* *sibiricus* MM 739 | YP_002994181 |
|  | *Thermococcus* *barophilus* MP | YP_004070427 |
|  | *Thermococcus* sp. 4557 | YP_004761857 |
|  |  | YP_004762353 |
|  | *Thermococcus* sp. CL1 | YP_006424428 |
| Archaea; Euryarchaeota; Thermoplasmata; Thermoplasmatales; | *Ferroplasma* *acidarmanus* fer1 | ZP_05570243 |
|  | *Picrophilus* *torridus* DSM 9790 | YP_024264 |
|  | *Thermoplasma* *volcanium* GSS1 | NP_111359 |
|  | *Thermoplasma* *acidophilum* DSM 1728 | NP_394105 |
| Archaea; Korarchaeota; Candidatus Korarchaeum | *Candidatus* Korarchaeum cryptofilum OPF8 | YP_001736738 |
| Bacteria; Acidobacteria; Acidobacteriales; | *Granulicella* *tundricola* MP5ACTX9 | YP_004217492 |
|  | *Terriglobus* *saanensis* SP1PR4 | YP_004184650 |
| Bacteria; Actinobacteria; Actinobacteridae; | *Actinomyces* sp. oral taxon 175 str. F0384 | ZP_08759727 |
|  | *Actinomyces* sp. ICM39 | ZP_10767126 |
|  | *Mobiluncus* *mulieris* ATCC 35243 | EEJ54743 |
|  | *Mobiluncus* *curtisii* subsp. *holmesii* ATCC 35242 | ZP_07910046 |
|  | *Corynebacterium* *glutamicum* ATCC 13032 | BAB99039 |
|  | *Corynebacterium* *nuruki* S6-4 | ZP_09129090 |
|  | *Dietzia* *cinnamea* P4 | ZP_08023186 |
|  | *Gordonia* sp. KTR9 | YP_006671520 |
|  | *Gordonia* *rhizosphera* NBRC 16068 | ZP_10944040 |
|  | *Mycobacterium* *ulcerans* Agy99 | YP_906459 |
|  | *Mycobacterium* *gilvum* Spyr1 | YP_004076699 |
|  | *Nocardia* *farcinica* IFM 10152 | YP_117181 |
|  | *Frankia* *alni* ACN14a | YP_711818 |
|  | Frankia symbiont of *Datisca* *glomerata* | YP_004582733 |
|  | *Brachybacterium* *faecium* DSM 4810 | YP_003156345 |
|  | *Janibacter* sp. HTCC2649 | ZP_00993754 |
|  | *Jonesia* *denitrificans* DSM 20603 | YP_003160454 |
|  | *Clavibacter* *michiganensis* subsp. *sepedonicus* | YP_001709472 |
|  | *Arthrobacter* *phenanthrenivorans* Sphe3 | YP_004241971 |
|  | *Micrococcus* *luteus* NCTC 2665 | YP_002958321 |
|  | *Rothia* *dentocariosa* ATCC 17931 | YP_003984535 |
|  | *Verrucosispora* *maris* AB-18-032 | YP_004408132 |
|  | *Nocardioides* sp. JS614 | YP_925774 |
|  | *Propionibacterium* *freudenreichii* subsp. *shermanii* CIRM-BIA1 | YP_003688243 |
|  | *Propionibacterium* *acidipropionici* ATCC 4875 | YP_006981912 |
|  | *Propionibacterium* *acnes* SK182B-JCVI | ZP_12575568 |
|  | *Pseudonocardia* *dioxanivorans* CB1190 | YP_004335068 |
|  | *Saccharomonospora* *azurea* NA-128 | ZP_09873668 |
|  | *Streptomyces* *avermitilis* MA-4680 = NBRC 14893 | NP_826180 |
|  | *Streptomyces* *griseus* subsp. *griseus* NBRC 13350 | YP_001827355 |
|  | *Streptomyces* sp. AA4 | ZP_07279229 |
|  | *Streptomyces* sp. Tu6071 | ZP_08454657 |
| Bacteria; Actinobacteria; Coriobacteridae; | *Atopobium* *rimae* ATCC 49626 | ZP_03567654 |
|  | *Atopobium* sp. ICM58 | ZP_10764468 |
| Bacteria; Actinobacteria; Rubrobacteridae; | *Rubrobacter* *xylanophilus* DSM 9941 | YP_643847 |
| Bacteria; Bacteroidetes; Bacteroidetes Order II. | *Salinibacter* *ruber* DSM 13855 | YP_446536 |
| Bacteria; Bacteroidetes; Bacteroidia; | *Bacteroides* *clarus* YIT 12056 | ZP_08296855 |
|  | *Alistipes* *shahii* WAL 8301 | CBK64321 |
| Bacteria; Bacteroidetes; Cytophagia; | *Cecembia* *lonarensis* LW9 | ZP_11071538 |
|  | *Fibrella* *aestuarina* BUZ 2 | CCG98659 |
| Bacteria; Bacteroidetes; Flavobacteriia; | *Blattabacterium* sp. (*Blattella* *germanica*) str. Bge | YP_003284075 |
| Bacteria; Bacteroidetes; Sphingobacteriia; | *Pedobacter* *saltans* DSM 12145 | YP_004275252 |
| Bacteria; Chlamydiae; Chlamydiales; | *Simkania* *negevensis* Z | YP_004671724 |
|  | *Waddlia* *chondrophila* 2032/99 | CCB91346 |
| Bacteria; Chloroflexi; Chloroflexales; | *Oscillochloris* *trichoides* DG-6 | ZP_07686363 |
| Bacteria; Chloroflexi; Thermomicrobiales; | *Thermomicrobium* *roseum* DSM 5159 | YP_002523638 |
| Bacteria; Cyanobacteria; Oscillatoriophycideae; | *Acaryochloris* sp. CCMEE 5410 | ZP_09248308 |
|  | *Cyanothece* sp. ATCC 51142 | YP_001804024 |
|  | *Synechococcus* sp. WH 7803 | YP_001224269 |
|  | *Arthrospira* *maxima* CS-328 | ZP_03273529 |
|  | *Coleofasciculus* *chthonoplastes* PCC 7420 | ZP_05028955 |
| Bacteria; Deferribacteres; Deferribacterales; | *Deferribacter* *desulfuricans* SSM1 | YP_003496792 |
| Bacteria; Deinococcus-Thermus; Deinococci; | *Deinococcus* *radiodurans* R1 | NP_294743 |
|  | *Deinococcus* *maricopensis* DSM 21211 | YP_004172422 |
|  | *Marinithermus* *hydrothermalis* DSM 14884 | YP_004367519 |
|  | *Thermus* *thermophilus* HB8 | YP_145382 |
| Bacteria; Firmicutes; Bacilli; | *Bacillus* *subtilis* BEST7613 | BAM54932 |
|  | *Bacillus* sp. NRRL B-14911 | ZP_01169666 |
|  | *Bacillus* sp. B14905 | ZP_01721828 |
|  | *Bacillus* *subtilis* subsp. *subtilis* str. 168 | ZP_03590616 |
|  | *Bacillus* *azotoformans* LMG 9581 | ZP_11313520 |
|  | *Bacillus* *anthracis* str. Ames | NP_843528 |
|  | *Geobacillus* *thermodenitrificans* NG80-2 | YP_001126186 |
|  | *Geobacillus* sp. WCH70 | YP_002950065 |
|  | *Halobacillus* *halophilus* DSM 2266 | YP_006181646 |
|  | *Brevibacillus* *brevis* NBRC 100599 | YP_002774517 |
|  | *Paenibacillus* *alvei* DSM 29 | ZP_10866902 |
|  | *Paenibacillus* *popilliae* ATCC 14706 | ZP_16293437 |
|  | *Sporolactobacillus* *vineae* DSM 21990 = SL153 | ZP_10969323 |
|  | *Bacillus* *selenitireducens* MLS10 | YP_003699012 |
|  |  | YP_003700075 |
|  | *Staphylococcus* *aureus* subsp. *aureus* MRSA252 | YP_040688 |
|  | *Staphylococcus* *hominis* SK119 | ZP_04059706 |
|  | *Staphylococcus* *aureus* subsp. *aureus* CIG290 | ZP_13541557 |
|  | *Enterococcus* *faecium* Com15 | ZP_05679719 |
|  | *Lactobacillus* *casei* ATCC 334 | YP_805944 |
|  | *Lactobacillus* *rhamnosus* HN001 | ZP_03210412 |
|  | *Lactobacillus* *animalis* KCTC 3501 | ZP_08548560 |
|  | *Lactobacillus* *farciminis* KCTC 3681 | ZP_08575922 |
|  | *Lactococcus* *lactis* subsp. *cremoris* A76 | YP_005876094 |
|  | *Streptococcus* *porcinus* str. *Jelinkova* 176 | ZP_08399741 |
|  | *Streptococcus* *ictaluri* 707-05 | ZP_09126963 |
|  | *Streptococcus* *oralis* SK100 | ZP_14306472 |
| Bacteria; Firmicutes; Clostridia; | *Clostridiales* *genomosp*. BVAB3 str. UPII9-5 | YP_003475727 |
|  | *Clostridium* *perfringens* WAL-14572 | ZP_16428527 |
|  | *Sulfobacillus* *acidophilus* TPY | YP_004720861 |
|  | *Acetobacterium* *woodii* DSM 1030 | YP_005269444 |
|  | *Eubacterium* *limosum* KIST612 | YP_003961020 |
|  | *Heliobacterium* *modesticaldum* Ice1 | YP_001680178 |
|  | Lachnospiraceae bacterium 3_1_57FAA_CT1 | ZP_08610514 |
|  | *Desulfotomaculum* *nigrificans* DSM 574 | ZP_08113214 |
|  | *Symbiobacterium* *thermophilum* IAM 14863 | YP_074256 |
|  |  | YP_075815 |
|  | *Halanaerobium* *hydrogeniformans* | YP_003993864 |
|  | *Halanaerobium* *praevalens* DSM 2228 | YP_005836013 |
|  | *Caldanaerobacter* *subterraneus* subsp. *tengcongensis* MB4 | NP_623575 |
|  | *Carboxydothermus* *hydrogenoformans* Z-2901 | YP_360658 |
|  | *Thermoanaerobacter* *siderophilus* SR4 | ZP_10305078 |
| Bacteria; Firmicutes; Erysipelotrichia; | Erysipelotrichaceae bacterium 6_1_45 | ZP_16409751 |
| Bacteria; Firmicutes; Negativicutes; | *Pelosinus* *fermentans* B4 | ZP_15516389 |
|  | *Selenomonas* sp. CM52 | ZP_10883299 |
|  | *Thermosinus* *carboxydivorans* Nor1 | ZP_01666923 |
| Bacteria; Fusobacteria; Fusobacteriales; | *Fusobacterium* *nucleatum* subsp. *fusiforme* ATCC 51190 | ZP_15604212 |
|  | *Fusobacterium* *nucleatum* subsp. *polymorphum* F0401 | ZP_16419585 |
|  | *Ilyobacter* *polytropus* DSM 2926 | YP_003967859 |
| Bacteria; Planctomycetes; Planctomycetia; | *Rhodopirellula* *baltica* SH 1 | NP_865708 |
|  | *Singulisphaera* *acidiphila* DSM 18658 | ZP_09571198 |
| Bacteria; Proteobacteria; Alphaproteobacteria; | *Bartonella* *grahamii* as4aup | YP_002972071 |
|  | Bradyrhizobiaceae bacterium SG-6C | ZP_08627298 |
|  | *Bradyrhizobium* sp. BTAi1 | YP_001238306 |
|  | *Bradyrhizobium* sp. WSM1253 | ZP_10082831 |
|  | *Ochrobactrum* *anthropi* CTS-325 | ZP_10967898 |
|  | *Methylobacterium* *radiotolerans* JCM 2831 | YP_001754857 |
|  | *Agrobacterium* sp. ATCC 31749 | ZP_08530727 |
|  | *Rhizobium* *lupini* HPC(L) | ZP_11198308 |
|  | *Sinorhizobium* *meliloti* SM11 | YP_005723462 |
|  | *Hirschia* *baltica* ATCC 49814 | YP_003061191 |
|  | *Celeribacter* *baekdonensis* B30 | ZP_11131853 |
|  | *Labrenzia* *alexandrii* DFL-11 | ZP_05115288 |
|  | *Octadecabacter* *arcticus* 238 | ZP_05065378 |
|  | *Pelagibaca* *bermudensis* HTCC2601 | ZP_01441100 |
|  | *Roseibium* sp. TrichSKD4 | ZP_07659307 |
|  | *Silicibacter* *lacuscaerulensis* ITI-1157 | ZP_05787968 |
|  | *Acidiphilium* *multivorum* AIU301 | YP_004285128 |
|  | *Gluconacetobacter* *oboediens* 174Bp2 | ZP_08898651 |
|  | Acetobacteraceae bacterium AT-5844 | ZP_09397651 |
|  | *Thalassospira* *profundimaris* WP0211 | ZP_11121319 |
|  | *Tistrella* *mobilis* KA081020-065 | YP_006374570 |
|  | *Sphingomonas* *elodea* ATCC 31461 | ZP_09958823 |
|  | *Sphingomonas* sp. PAMC 26617 | ZP_10425396 |
|  | *Sphingopyxis* *alaskensis* RB2256 | YP_615246 |
| Bacteria; Proteobacteria; Betaproteobacteria; | *Achromobacter* *piechaudii* ATCC 43553 | ZP_06688535 |
|  | *Bordetella* *pertussis* Tohama I | NP_881260 |
|  | *Burkholderia* *rhizoxinica* HKI 454 | YP_004027742 |
|  | *Cupriavidus* *metallidurans* CH34 | YP_587572 |
|  | *Ralstonia* *solanacearum* UW551 | ZP_00946852 |
|  | *Acidovorax* sp. NO-1 | ZP_09330941 |
|  | *Verminephrobacter* *eiseniae* EF01-2 | YP_996788 |
|  | *Leptothrix* *cholodnii* SP-6 | YP_001792244 |
|  | *Sideroxydans* *lithotrophicus* ES-1 | YP_003525071 |
|  | *Pseudogulbenkiania* sp. NH8B | YP_004849272 |
|  | *Simonsiella* *muelleri* ATCC 29453 | ZP_10984245 |
|  | *Azoarcus* sp. KH32C | BAL23536 |
| Bacteria; Proteobacteria; Deltaproteobacteria | delta proteobacterium NaphS2 | ZP_07205020 |
|  | *Desulfobacterium* *autotrophicum* HRM2 | YP_002601608 |
|  | *Desulfobacula* *toluolica* Tol2 | YP_006761709 |
|  | *Desulfovibrio* *vulgaris* str. Hildenborough | YP_011157 |
|  |  | YP_011885 |
|  | *Desulfovibrio* *desulfuricans* subsp. *desulfuricans* str. ATCC 27774 | YP_002480101 |
|  | *Geobacter* sp. M18 | YP_004200166 |
|  | *Anaeromyxobacter* sp. Fw109-5 | YP_001378528 |
| Bacteria; Proteobacteria; Epsilonproteobacteria; | *Helicobacter* *felis* ATCC 49179 | YP_004073920 |
| Bacteria; Proteobacteria; Gammaproteobacteria | gamma proteobacterium HdN1 | YP_003812610 |
|  | *Aeromonas* *caviae* Ae398 | ZP_08521014 |
|  | *Glaciecola* *psychrophila* 170 | ZP_11323085 |
|  | *Marinobacter* sp. BSs20148 | YP_006558769 |
|  | *Marinobacter* sp. ELB17 | ZP_01739057 |
|  | *Moritella* sp. PE36 | ZP_01897076 |
|  | *Pseudoalteromonas* sp. Bsw20308 | ZP_11405940 |
|  | *Citrobacter* *rodentium* ICC168 | YP_003367805 |
|  | *Citrobacter* *freundii* 4_7_47CFAA | ZP_09336341 |
|  | *Enterobacter* sp. 638 | YP_001178541 |
|  | *Escherichia* *coli* | 2R4J_A |
|  | *Escherichia* *coli* K-12 | P0A9C0 |
|  | *Morganella* *morganii* SC01 | ZP_11283921 |
|  | *Photorhabdus* *luminescens* subsp. *laumondii* TTO1 | NP_927559 |
|  | *Salmonella* *enterica* subsp. *enterica* serovar Paratyphi B str. SPB7 | YP_001590530 |
|  | *Salmonella* *enterica* subsp. *enterica* serovar Heidelberg str. SL476 | YP_002046338 |
|  | *Yersinia* *frederiksenii* ATCC 33641 | ZP_04632780 |
|  | *Legionella* *pneumophila* str. Lens | YP_126714 |
|  | *Marinomonas* *posidonica* IVIA-Po-181 | YP_004480354 |
|  | *Actinobacillus* *minor* 202 | ZP_05628993 |
|  | *Aggregatibacter* *actinomycetemcomitans* serotype e str. SC1083 | ZP_11577631 |
|  | *Haemophilus* *influenzae* PittGG | YP_001292612 |
|  | *Pasteurella* *bettyae* CCUG 2042 | ZP_10125760 |
|  | *Acinetobacter* sp. NCTC 10304 | ZP_10936832 |
|  | *Pseudomonas* sp. S9 | ZP_09709455 |
|  | *Pseudomonas* sp. GM102 | ZP_10596622 |
|  | *Pseudomonas* sp. GM55 | ZP_10645168 |
|  | *Francisella* *tularensis* subsp. *mediasiatica* FSC147 | YP_001891056 |
|  | *Grimontia* *hollisae* CIP 101886 | ZP_06053660 |
|  | *Photobacterium* *profundum* SS9 | YP_129585 |
|  | *Vibrio* *parahaemolyticus* RIMD 2210633 | NP_798767 |
|  | *Vibrio* *vulnificus* MO6-24/O | YP_004190689 |
|  | *Vibrio* *caribbenthicus* ATCC BAA-2122 | ZP_07743946 |
|  | *Stenotrophomonas* *maltophilia* JV3 | YP_004794105 |
|  | *Xanthomonas* *axonopodis* pv. *punicae* str. LMG 859 | ZP_10261464 |
|  | *Xylella* *fastidiosa* 9a5c | NP_299545 |
| Bacteria; Spirochaetes; Spirochaetales; | *Brachyspira* *pilosicoli* WesB | CCG57584 |
|  | *Leptospira* *broomii* serovar Hurstbridge str. 5399 | ZP_10531998 |
|  | *Leptospira* *interrogans* str. UI 08452 | ZP_13277471 |
|  | *Leptospira* *interrogans* str. Brem 329 | ZP_15582285 |
|  | *Turneriella* *parva* DSM 21527 | YP_006439233 |
|  | *Borrelia* *burgdorferi* | ABQ43062 |
|  | *Borrelia* *burgdorferi* ZS7 | ABQ43065 |
|  | *Borrelia* *valaisiana* VS116 | ZP_03672365 |
|  | *Spirochaeta* *smaragdinae* DSM 11293 | YP_003805225 |
|  | *Treponema* *phagedenis* F0421 | ZP_08036972 |
| Bacteria; Synergistetes; Synergistia; | *Pyramidobacter* *piscolens* W5455 | ZP_06265733 |
| Bacteria; Tenericutes; Mollicutes; | *Mycoplasma* *hyorhinis* HUB-1 | YP_003856265 |
|  | *Mycoplasma* *mycoides* subsp. *capri* LC str. 95010 | YP_004399986 |
|  | *Mycoplasma* *pneumoniae* 309 | YP_005175284 |
| Bacteria; Thermotogae; Thermotogales; | *Kosmotoga* *olearia* TBF 19.5.1 | YP_002939889 |
|  | *Marinitoga* *piezophila* KA3 | YP_005096443 |
|  | *Thermosipho* *melanesiensis* BI429 | YP_001305869 |
|  | *Thermotoga* *maritima* MSB8 | ZP_12683583 |

Supplementary Table S4. The list of sequence entries used to infer the GK (GlpK) tree.

| Taxonomy | Organism | Accession |
| --- | --- | --- |
| Archaea; Aigarchaeota | Aigarchaeota archaeon JGI 0000106-J15 | WP_042660898 |
|  |  | WP_042660900 |
|  | Thaumarchaeota archaeon JGI OTU-3 | WP_042663342 |
| Archaea; Crenarchaeota; Thermoprotei; Desulfurococcales; | *Aeropyrum* *pernix* K1 | BAA79261 |
|  | *Desulfurococcus* *fermentans* DSM 16532 | AFL66797 |
|  | *Staphylothermus* *marinus* F1 | ABN69377 |
| Archaea; Crenarchaeota; Thermoprotei; Sulfolobales; | *Sulfolobus* *acidocaldarius* DSM 639 | AAY80469 |
|  | *Sulfolobus* *solfataricus* P2 | NP_343018 |
| Archaea; Crenarchaeota; Thermoprotei; Thermoproteales; | *Thermofilum* *pendens* Hrk 5 | ABL78526 |
|  |  | ABL78770 |
|  | *Pyrobaculum* *oguniense* | WP_014347509 |
|  | *Vulcanisaeta* *distributa* DSM 14429 | ADN49680 |
|  |  | ADN50882 |
| Archaea; Euryarchaeota; | *Aciduliprofundum* *boonei* T469 | ADD09236 |
| Archaea; Euryarchaeota; Archaeoglobi; Archaeoglobales; | *Archaeoglobus* *fulgidus* DSM 4304 | AAB90370 |
|  | *Archaeoglobus* *fulgidus* DSM 8774 | AIG98753 |
| Archaea; Euryarchaeota; candidate division pMC2A384 | Euryarchaeota archaeon SCGC AAA252-I15 | WP_041899301 |
| Archaea; Euryarchaeota; environmental samples | uncultured marine group II/III euryarchaeote KM3_86_F07 | AIF19344 |
| Archaea; Euryarchaeota; Halobacteria; Halobacteriales | halophilic archaeon J07HX5 | ERG88035 |
|  | *Halarchaeum* *acidiphilum* MH1-52-1 | GAD52556 |
|  | *Halalkalicoccus* *jeotgali* B3 | ELY36074 |
|  | *Halobacterium* *salinarum* R1 | B0R6S2 |
|  | *Halococcus* *saccharolyticus* DSM 5350 | EMA43995 |
|  | *Haloferax* *volcanii* DS2 | ADE04400 |
|  | *Halorhabdus* *tiamatea* SARL4B | CCQ34342 |
|  |  | ERJ06771 |
|  | *Halosimplex* *carlsbadense* 2-9-1 | ELZ24248 |
|  | *Natronomonas* *moolapensis* 8.8.11 | CCQ35593 |
|  |  | CCQ37762 |
| Archaea; Euryarchaeota; Methanomicrobia; | *Methanosaeta* *thermophila* | WP_011696420 |
| Archaea; Euryarchaeota; Thermococci; Thermococcales; | *Palaeococcus* *pacificus* DY20341 | AIF68948 |
|  |  | AIF70086 |
|  | *Pyrococcus* *abyssi* GE5 | CCE69644 |
|  | *Thermococcus* *kodakarensis* KOD1 | BAA34909 |
| Archaea; Euryarchaeota; Thermoplasmata; Thermoplasmatales; | *Acidiplasma* sp. MBA-1 | KJE50097 |
|  | *Ferroplasma* *acidarmanus* fer1 | AGO61823 |
|  | *Picrophilus* *torridus* DSM 9790 | CBG09962 |
|  | *Thermoplasma* *acidophilum* DSM 1728 | CBG09912 |
|  | *Thermoplasma* *volcanium* GSS1 | BAB60308 |
| Archaea; Korarchaeota; Candidatus Korarchaeum | *Candidatus* Korarchaeum cryptofilum OPF8 | ACB07057 |
| Archaea; Lokiarchaeota; 'Lokiarchaeum' | archaeon Loki | KKK40876 |
| Archaea; Parvarchaeota; Candidatus Parvarchaeum | *Candidatus* Parvarchaeum acidophilus ARMAN-5 | EFD93071 |
| Archaea; Thaumarchaeota; unclassified Thaumarchaeota | *Candidatus* Caldiarchaeum subterraneum | BAJ46744 |
| Bacteria; Acetothermia | Acetothermia bacterium SCGC AAA255-C06 | WP_029957707 |
| Bacteria; Acidobacteria; Acidobacteriales; Acidobacteriaceae; | *Acidobacterium* *capsulatum* ATCC 51196 | ACO34171 |
|  | *Edaphobacter* *aggregans* | WP_035358925 |
| Bacteria; Acidobacteria; Acidobacteria subdivision 4; | *Chloracidobacterium* *thermophilum* | WP_014099661 |
| Bacteria; Acidobacteria; | *Candidatus* Koribacter versatilis Ellin345 | ABF41988 |
| Bacteria; Acidobacteria; Solibacteres; | *Candidatus* Solibacter usitatus Ellin6076 | ABJ86502 |
|  | *Bryobacter* *aggregatus* | WP_031495195 |
| Bacteria; Actinobacteria; Acidimicrobidae; | *Acidimicrobium* *ferrooxidans* DSM 10331 | ACU53821 |
|  |  | ACU54395 |
| Bacteria; Actinobacteria; Acidimicrobiia; | *Acidithrix* *ferrooxidans* | KJF16125 |
|  | *Ilumatobacter* *nonamiensis* | WP_040492385 |
|  |  | WP_040494833 |
| Bacteria; Actinobacteria; | *Candidatus* Microthrix parvicella RN1 | CCM62868 |
|  |  | CCM63116 |
| Bacteria; Actinobacteria; Catenulisporales; | *Actinospica* *robiniae* | WP_034270735 |
| Bacteria; Actinobacteria; Coriobacteridae; | *Coriobacterium* *glomerans* PW2 | AEB06307 |
|  |  | AEB07709 |
|  | *Cryptobacterium* *curtum* DSM 15641 | ACU94901 |
| Bacteria; Actinobacteria; Coriobacteriia; | *Collinsella* *stercoris* DSM 13279 | EEA91608 |
|  | *Eggerthella* *lenta* 1_1_60AFAA | KGI71311 |
| Bacteria; Actinobacteria; Corynebacteriales; | *Nocardia* *cyriacigeorgica* GUH-2 | CCF61844 |
|  |  | CCF63523 |
|  |  | CCF63525 |
| Bacteria; Actinobacteria; Frankiales; | *Frankia* *alni* ACN14a | CAJ63208 |
| Bacteria; Actinobacteria; Geodermatophilales; | *Modestobacter* *marinus* | WP_014741070 |
| Bacteria; Actinobacteria; Micrococcales; | *Demetria* *terragena* | WP_018157864 |
|  | *Knoellia* *aerolata* DSM 18566 | KGN40074 |
|  | *Agrococcus* *pavilionensis* RW1 | ERG63217 |
|  |  | ERG64268 |
|  | *Glaciibacter* *superstes* | WP_022885734 |
|  | *Gulosibacter* *molinativorax* | WP_026935893 |
| Bacteria; Actinobacteria; Micromonosporales; | *Micromonospora* *lupini* | WP_007455927 |
|  |  | WP_039906841 |
| Bacteria; Actinobacteria; Propionibacteriales; | *Aeromicrobium* *marinum* DSM 15272 | EFQ83688 |
|  | *Marmoricola* *aequoreus* | WP_030484549 |
|  |  | WP_030486057 |
|  |  | WP_030486066 |
| Bacteria; Actinobacteria; Pseudonocardiales; | *Actinomycetospora* *chiangmaiensis* | WP_018333955 |
| Bacteria; Actinobacteria; Rubrobacteria; | *Rubrobacter* *xylanophilus* DSM 9941 | CBG09994 |
| Bacteria; Actinobacteria; Rubrobacteridae; | *Conexibacter* *woesei* DSM 14684 | ADB52334 |
| Bacteria; Actinobacteria; Streptomycetales; | *Streptomyces* *coelicolor* A3(2) | CBG09919 |
|  |  | NP_625935 |
| Bacteria; Actinobacteria; Thermoleophilia; | *Patulibacter* *americanus* | WP_022926992 |
|  |  | WP_022928288 |
|  | *Solirubrobacter* *soli* | WP_028064102 |
|  |  | WP_028066920 |
|  |  | WP_037503402 |
| Bacteria; Aerophobetes; | *Candidatus* Aerophobus profundus | WP_041887985 |
|  |  | WP_041888568 |
|  |  | WP_041889415 |
|  |  | WP_041891372 |
| Bacteria; Aquificae; Aquificales; | *Aquifex* *aeolicus* VF5 | AAC06710 |
| Bacteria; Armatimonadetes; Chthonomonadetes; | *Chthonomonas* *calidirosea* | WP_016483400 |
| Bacteria; Armatimonadetes; | *Fimbriimonas* *ginsengisoli* Gsoil 348 | AIE83569 |
| Bacteria; Atribacteria; | Atribacteria bacterium JGI 0000059-I14 | WP_020264864 |
|  | Atribacteria bacterium SCGC AAA255-G05 | WP_029717935 |
|  | Atribacteria bacterium SCGC AAA255-N14 | WP_029955352 |
|  | Atribacteria bacterium SCGC AB-164-A22 | WP_038960502 |
|  |  | WP_038960838 |
|  | *Candidatus* Caldatribacterium saccharofermentans | WP_017873041 |
|  |  | WP_017873216 |
|  |  | WP_017873430 |
|  |  | WP_017873676 |
|  |  | WP_017874083 |
|  |  | WP_038304983 |
|  |  | WP_038305191 |
| Bacteria; Bacteroidetes; Bacteroidetes Order II. | *Salinibacter* *ruber* DSM 13855 | ABC43677 |
|  | *Salisaeta* *longa* | WP_022834800 |
| Bacteria; Bacteroidetes; Bacteroidia; | *Bacteroides* *pectinophilus* CAG:437 | CDD58737 |
|  | *Draconibacterium* *orientale* | AHW60297 |
|  | *Anaerophaga* *thermohalophila* | WP_016775946 |
|  | *Odoribacter* *laneus* CAG:561 | CCZ79985 |
|  | *Prolixibacter* *bellariivorans* | WP_025864903 |
| Bacteria; Bacteroidetes; Cytophagia; | *Cyclobacterium* *marinum* DSM 745 | AEL26147 |
|  | *Flexibacter* *elegans* | WP_027000030 |
|  | *Flammeovirga* *pacifica* | WP_044222896 |
| Bacteria; Bacteroidetes; Flavobacteriia; | *Arenibacter* *latericius* | WP_026809172 |
|  |  | WP_026809319 |
| Bacteria; Bacteroidetes; Sphingobacteriia; | *Niastella* *koreensis* GR20-10 | AEV96471 |
|  |  | AEV97103 |
|  | *Saprospira* *grandis* DSM 2844 | EJF54771 |
|  | *Sphingobacterium* *spiritivorum* ATCC 33300 | EEI90335 |
| Bacteria; Caldiserica; Caldisericia; | *Caldisericum* *exile* AZM16c01 | BAL81566 |
| Bacteria; candidate division BRC1 | BRC1 bacterium SCGC AAA252-M09 | WP_020249390 |
|  | BRC1 bacterium SCGC AAA257-C11 | WP_029711590 |
|  |  | WP_029712137 |
| Bacteria; Chlamydiae; Chlamydiales; | *Chlamydia* *trachomatis* | CRH65459 |
|  |  | CRH83989 |
|  | *Simkania* *negevensis* Z | CCB89231 |
|  | *Waddlia* *chondrophila* WSU 86-1044 | ADI38647 |
| Bacteria; Chlorobi; Chlorobia; | *Chlorobium* *ferrooxidans* DSM 13031 | EAT59240 |
| Bacteria; Chloroflexi; Anaerolineae; | *Anaerolinea* *thermophila* UNI-1 | BAJ64787 |
| Bacteria; Chloroflexi; Caldilineae; | *Caldilinea* *aerophila* DSM 14535 = NBRC 104270 | BAL98714 |
|  |  | BAL98869 |
|  |  | BAM01359 |
| Bacteria; Chloroflexi; Chloroflexia; | *Chloroflexus* *aggregans* DSM 9485 | ACL23025 |
|  |  | ACL26565 |
|  | *Herpetosiphon* *aurantiacus* DSM 785 | ABX03446 |
| Bacteria; Chloroflexi; Ktedonobacteria; | *Ktedonobacter* *racemifer* DSM 44963 | EFH86008 |
| Bacteria; Chloroflexi; Sphaerobacteridae; | *Sphaerobacter* *thermophilus* DSM 20745 | ACZ40521 |
| Bacteria; Chloroflexi; | *Thermorudis* *peleae* | WP_038037768 |
| Bacteria; Cyanobacteria; Gloeobacteria; | *Gloeobacter* *violaceus* PCC 7421 | BAC89692 |
| Bacteria; Cyanobacteria; Nostocales; | *Hassallia* *byssoidea* VB512170 | KIF30382 |
|  |  | KIF33408 |
|  | *Tolypothrix* *campylonemoides* | WP_041032975 |
|  | *Anabaena* *cylindrica* | WP_015217632 |
|  | *Nostoc* *punctiforme* PCC 73102 | ACC80253 |
|  | *Scytonema* *millei* VB511283 | KIF24116 |
| Bacteria; Cyanobacteria; Oscillatoriophycideae; | *Cyanobacterium* *aponinum* PCC 10605 | AFZ53899 |
|  | *Crinalium* *epipsammum* PCC 9333 | AFZ11677 |
|  | *Leptolyngbya* *boryana* | WP_017290955 |
|  | *Lyngbya* *confervoides* BDU141951 | KIF41412 |
|  | *Oscillatoria* *acuminata* PCC 6304 | AFY82853 |
| Bacteria; Cyanobacteria; Stigonematales; | *Mastigocoleus* *testarum* | WP_027840683 |
| Bacteria; Deferribacteres; Deferribacterales; | *Deferribacter* *desulfuricans* SSM1 | BAI80698 |
|  | *Flexistipes* *sinusarabici* DSM 4947 | AEI15226 |
|  | *Mucispirillum* *schaedleri* ASF457 | ESJ97697 |
| Bacteria; Deinococcus-Thermus; Deinococci; | *Deinococcus* *radiodurans* R1 | AAF11475 |
|  | *Truepera* *radiovictrix* DSM 17093 | ADI13588 |
|  |  | ADI15622 |
|  | *Thermus* *thermophilus* | BAA28283 |
|  |  | WP_011229157 |
| Bacteria; Dictyoglomi; Dictyoglomales; | *Dictyoglomus* *thermophilum* H-6-12 | ACI19444 |
| Bacteria; Elusimicrobia; Elusimicrobia; | *Elusimicrobium* *minutum* Pei191 | ACC98622 |
| Bacteria; Fibrobacteres; Fibrobacterales; | *Fibrobacter* *succinogenes* | WP_012820140 |
| Bacteria; Firmicutes; Bacilli; | *Alicyclobacillus* *macrosporangiidus* | WP_029421137 |
|  |  | WP_029421276 |
|  |  | WP_029422805 |
|  | *Bacillus* *subtilis* subsp. *subtilis* str. 168 | AIY92216 |
|  | *Bacillus* *cereus* ATCC 4342 | AJH72057 |
|  | *Thermicanus* *aegyptius* | WP_028986224 |
|  | *Exiguobacterium* *acetylicum* | WP_029340955 |
|  |  | WP_029341249 |
|  | *Jeotgalicoccus* *marinus* | WP_026867402 |
|  | *Listeria* *monocytogenes* EGD-e | NP_464559 |
|  |  | NP_465063 |
|  | *Sporolactobacillus* *terrae* | WP_028977622 |
|  | *Staphylococcus* *aureus* subsp. *aureus* DSM 20231 | ELP29126 |
|  | *Thermoactinomyces* *daqus* | WP_033101575 |
|  |  | WP_033102324 |
|  | *Carnobacterium* *gallinarum* | WP_034559134 |
|  |  | WP_034561444 |
|  | *Lactobacillus* *casei* 32G | EKP97237 |
|  | *Lactococcus* *piscium* MKFS47 | CEN27344 |
|  |  | CEN28360 |
| Bacteria; Firmicutes; Clostridia; | *Caldicoprobacter* *oshimai* | WP_025746882 |
|  | *Catabacter* *hongkongensis* | KKI52366 |
|  |  | WP_046442552 |
|  |  | WP_046442557 |
|  |  | WP_046442788 |
|  |  | WP_046443512 |
|  |  | WP_046443675 |
|  |  | WP_046443790 |
|  | *Clostridium* *novyi* NT | ABK60806 |
|  |  | ABK62551 |
|  | *Kallipyga* *massiliensis* | WP_034578917 |
|  | *Anaerovorax odorimutans* | WP_027399349 |
|  | *Sulfobacillus thermosulfidooxidans* | WP_028962118 |
|  | *Symbiobacterium* *thermophilum* IAM 14863 | BAD40180 |
|  | *Eubacterium* *hallii* CAG:12 | CDB17460 |
|  |  | CDB17563 |
|  |  | CDB18741 |
|  |  | CDB19038 |
|  |  | WP_022169388 |
|  | *Heliobacterium* *modesticaldum* Ice1 | ABZ84166 |
|  | *Lachnospira multipara* | WP_027431885 |
|  | *Oscillibacter ruminantium* | WP_040659376 |
|  |  | WP_040662368 |
|  | *Peptoclostridium* *difficile* P78 | EQK08286 |
|  |  | EQK09940 |
|  | *Proteocatella* *sphenisci* | WP_028830227 |
|  | *Anaerotruncus* *colihominis* DSM 17241 | EDS11102 |
|  | *Halanaerobium* *hydrogeniformans* | WP_013404615 |
|  |  | WP_013405222 |
|  |  | WP_013405552 |
|  |  | WP_013405669 |
|  | *Halobacteroides* *halobius* DSM 5150 | AGB41942 |
|  | *Natranaerobius* *thermophilus* JW/NM-WN-LF | ACB86230 |
|  | *Thermoanaerobacterium* *thermosaccharolyticum* DSM 571 | ADL68147 |
|  | *Coprothermobacter* *proteolyticus* DSM 5265 | ACI18093 |
| Bacteria; Firmicutes; Erysipelotrichia; | *Erysipelothrix* *rhusiopathiae* ATCC 19414 | EFY08374 |
| Bacteria; Firmicutes; Negativicutes; | *Phascolarctobacterium* *succinatutens* YIT 12067 | EFY05565 |
|  | *Mitsuokella* *jalaludinii* | WP_036378368 |
|  |  | WP_036378404 |
| Bacteria; Fusobacteria; Fusobacteriales; Sebaldella | *Cetobacterium* *somerae* ATCC BAA-474 | ERT68679 |
|  | *Fusobacterium* *nucleatum* CC53 | EMP16676 |
|  | *Ilyobacter* *polytropus* DSM 2926 | ADO81807 |
|  | *Psychrilyobacter* *atlanticus* | WP_028855010 |
|  | *Leptotrichia* *goodfellowii* F0264 | EEY35146 |
|  | *Sebaldella* *termitidis* ATCC 33386 | ACZ08914 |
| Bacteria; Gemmatimonadetes; Gemmatimonadales; | *Gemmatimonas* *aurantiaca* T-27 | BAH37860 |
| Bacteria; Haloplasmatales; Haloplasmataceae; | *Haloplasma* *contractile* SSD-17B | ERJ11633 |
| Bacteria; Lentisphaerae; Lentisphaeria; | *Lentisphaera* *araneosa* HTCC2155 | EDM24862 |
| Bacteria; Planctomycetes; Phycisphaerae; | *Phycisphaera* *mikurensis* NBRC 102666 | BAM04495 |
| Bacteria; Planctomycetes; Planctomycetia; | *Gemmata* *obscuriglobus* | WP_010040912 |
|  | *Planctopirus* *limnophila* DSM 3776 | ADG67832 |
|  | *Singulisphaera* *acidiphila* DSM 18658 | AGA27507 |
| Bacteria; Poribacteria | *Candidatus* Poribacteria sp. WGA-4E | WP_020382788 |
|  | Poribacteria bacterium WGA-3G | WP_022813357 |
| Bacteria; Proteobacteria; Alphaproteobacteria; | *Candidatus* Phaeomarinobacter ectocarpi | CDO58334 |
|  | *Geminicoccus roseus* | WP_027133847 |
|  |  | WP_027133908 |
|  |  | WP_027135507 |
|  | *Kiloniella spongiae* | KLN62498 |
|  | *Kordiimonas gwangyangensis* | WP_025896661 |
|  | *Micavibrio* *aeruginosavorus* EPB | AGH97359 |
|  | *Parvularcula* *bermudensis* HTCC2503 | ADM10411 |
|  | *Polymorphum* *gilvum* SL003B-26A1 | ADZ69494 |
|  | *Aureimonas* *ureilytica* | WP_019995749 |
|  | *Bartonella* *tamiae* Th239 | EJF90772 |
|  | *Beijerinckia* *indica* subsp. *indica* ATCC 9039 | ACB94033 |
|  | *Bradyrhizobium* *japonicum* SEMIA 5079 | AHY56740 |
|  | *Devosia geojensis* | KKB07069 |
|  | *Methyloceanibacter caenitepidi* | WP_045370012 |
|  | *Methylocystis parvus* | WP_016921063 |
|  | *Lutibaculum* *baratangense* AMV1 | ESR23373 |
|  | *Acetobacter* *aceti* NBRC 14818 | GAN57735 |
|  |  | GAN58394 |
|  | *Rhodospirillum* *rubrum* ATCC 11170 | ABC22809 |
|  |  | ABC24255 |
|  | *Reyranella* *massiliensis* | WP_020699470 |
|  | *Candidatus* Pelagibacter ubique HIMB083 | ETA70016 |
|  | *Candidatus* Puniceispirillum marinum IMCC1322 | ADE39127 |
|  | *Erythrobacter* *longus* | KEO90226 |
| Bacteria; Proteobacteria; Betaproteobacteria; | *Aquincola tertiaricarbonis* | WP_046115146 |
|  | *Burkholderia nodosa* | WP_028203060 |
|  |  | WP_028207051 |
|  | *Delftia* *acidovorans* SPH-1 | ABX33669 |
|  |  | ABX33766 |
|  | *Parasutterella* *excrementihominis* CAG:233 | CCX86848 |
|  | *Sutterella* *parvirubra* YIT 11816 | EHY30626 |
|  | *Thiomonas* *intermedia* K12 | ADG29963 |
|  | *Candidatus* Accumulibacter phosphatis clade IIA str. UW-1 | ACV35626 |
|  | *Sideroxydans* *lithotrophicus* ES-1 | ADE12683 |
|  | *Thiobacillus* *thioparus* | WP_018506836 |
|  | *Conchiformibius* *steedae* | WP_027021423 |
|  | *Nitrosomonas* *eutropha* C91 | ABI60303 |
|  | *Nitrosospira* *lacus* | CCU63268 |
|  | *Uliginosibacterium* *gangwonense* | WP_018609192 |
| Bacteria; Proteobacteria; Deltaproteobacteria; | *Bdellovibrio* *bacteriovorus* HD100 | CBG09937 |
|  | *Halobacteriovorax* *marinus* SJ | CBW28167 |
|  | *Desulfarculus* *baarsii* DSM 2075 | ADK85622 |
|  | *Desulfobulbus japonicus* | WP_028579727 |
|  |  | WP_028581428 |
|  | *Desulfovibrio inopinatus* | WP_027184207 |
|  | *Geobacter* *lovleyi* SZ | ACD96401 |
|  | *Geopsychrobacter* *electrodiphilus* | WP_020674827 |
|  | *Pelobacter* *seleniigenes* | WP_029914222 |
|  | *Anaeromyxobacter* *dehalogenans* 2CP-C | ABC83224 |
|  | *Enhygromyxa* *salina* | KIG17860 |
|  | *Haliangium* *ochraceum* DSM 14365 | ACY12932 |
|  | *Sandaracinus* *amylolyticus* | AKF05413 |
| Bacteria; Proteobacteria; Epsilonproteobacteria; | *Arcobacter* *nitrofigilis* DSM 7299 | ADG92542 |
|  | *Helicobacter* *bizzozeronii* CCUG 35545 | CCF81739 |
| Bacteria; Proteobacteria; Gammaproteobacteria; | *Thiobacillus* *prosperus* | KFZ89198 |
|  | *Aeromonas* *caviae* | KGY79666 |
|  | *Anaerobiospirillum* *succiniciproducens* | WP_027939739 |
|  | *Aliagarivorans* *marinus* | WP_026970640 |
|  |  | WP_035480402 |
|  | *Gilvimarinus agarilyticus* | WP_041524117 |
|  | *Colwellia psychrerythraea* | KGJ91175 |
|  | *Ferrimonas futtsuensis* | WP_028108982 |
|  |  | WP_028109277 |
|  | *Cardiobacterium* *valvarum* F0432 | EHM55950 |
|  | *Nitrococcus* *mobilis* Nb-231 | EAR23033 |
|  | *Candidatus* Competibacter denitrificans Run_A_D11 | CDI01750 |
|  | *Escherichia* *coli* str. K-12 substr. MG1655 | AAB03058 |
|  | *Coxiella* *burnetii* RSA 331 | ABX77758 |
|  | *Tatlockia* *micdadei* | CEG61109 |
|  | *Methylobacter luteus* | WP_027157124 |
|  | *Methylocaldum szegediense* | WP_026610635 |
|  | *Methyloglobulus* *morosus* KoM1 | ESS74003 |
|  | *Methylohalobius* *crimeensis* | WP_022949983 |
|  | *Alcanivorax* *jadensis* T9 | KGD60522 |
|  | *Balneatrix alpica* | WP_027313529 |
|  | *Halomonas zincidurans* | WP_031383819 |
|  | *Marinomonas profundimaris* | ETI59307 |
|  | *Oceanobacter kriegii* | WP_028294836 |
|  | *Congregibacter* *litoralis* KT71 | EAQ99339 |
|  | *Porticoccus* *hydrocarbonoclasticus* | WP_036860721 |
|  | *Enhydrobacter* *aerosaccus* SK60 | EEV22411 |
|  | *Pseudomonas* *alcaligenes* NBRC 14159 | GAD63814 |
|  | *Salinisphaera* *hydrothermalis* C41B8 | KEZ76838 |
|  | *Sedimenticola* *selenatireducens* | WP_029132444 |
|  | *Simiduia* *agarivorans* SA1 = DSM 21679 | AFV00906 |
|  | *Fangia* *hongkongensis* | WP_026196563 |
|  | *Francisella* *noatunensis* | WP_014714671 |
|  | *Piscirickettsia* *salmonis* LF-89 = ATCC VR-1361 | ERL61157 |
|  | *Thiothrix* *flexilis* | WP_020558557 |
|  | *Algiphilus* *aromaticivorans* | WP_043769594 |
|  | *Xanthomonas* *fuscans* | WP_007968439 |
| Bacteria; Spirochaetes; Brachyspirales; | *Brachyspira* *alvinipulli* | WP_028328769 |
| Bacteria; Spirochaetes; Leptospirales; | *Leptonema* *illini* DSM 21528 | EHQ06486 |
|  | *Leptospira* *noguchii* str. Hook | EMS83915 |
|  |  | EMS89436 |
|  | *Turneriella* *parva* DSM 21527 | AFM14818 |
| Bacteria; Spirochaetes; Spirochaetales; | *Borrelia* *afzelii* PKo | AEL69469 |
|  | *Salinispira* *pacifica* | AHC14043 |
|  |  | WP_024268781 |
|  | *Spirochaeta* *smaragdinae* DSM 11293 | ADK80159 |
|  |  | ADK80772 |
|  |  | ADK82629 |
|  |  | ADK82890 |
|  | *Treponema* *socranskii* | WP_016520350 |
|  |  | WP_016520711 |
|  |  | WP_038080401 |
| Bacteria; Synergistetes; Synergistia; | *Aminomonas* *paucivorans* | WP_006301602 |
|  | *Synergistes* sp. 3_1_syn1 | EHL64432 |
| Bacteria; Tenericutes; Mollicutes; | *Acholeplasma* *brassicae* | CCV65093 |
|  |  | CCV65925 |
| Bacteria; | *Thermobaculum* *terrenum* ATCC BAA-798 | ACZ41391 |
| Bacteria; Thermotogae; Kosmotogales; | *Mesotoga* *prima* MesG1.Ag.4.2 | AFK06476 |
|  |  | AFK08044 |
| Bacteria; Thermotogae; Petrotogales; | *Defluviitoga* *tunisiensis* | CEP78158 |
| Bacteria; Thermotogae; Thermotogales; | *Fervidobacterium* *pennivorans* DSM 9078 | AFG34289 |
|  |  | AFG34359 |
|  | *Kosmotoga* *olearia* TBF 19.5.1 | ACR79254 |
|  | *Marinitoga* *piezophila* KA3 | AEX84915 |
|  | *Petrotoga* *mobilis* SJ95 | ABX31026 |
|  |  | ABX31179 |
|  | *Pseudothermotoga* *thermarum* DSM 5069 | AEH51076 |
|  | *Thermosipho* *africanus* TCF52B | ACJ75139 |
|  | *Thermotoga* *maritima* MSB8 | AGL49879 |
|  |  | Q9X1E4 |
| Bacteria; Verrucomicrobia; Opitutae; | *Opitutus* *terrae* PB90-1 | ACB74772 |
|  | *Coraliomargarita* *akajimensis* DSM 45221 | ADE55240 |
| Bacteria; Verrucomicrobia; unclassified Verrucomicrobia; | *Methylacidiphilum* *infernorum* V4 | ACD83757 |
| Bacteria; Verrucomicrobia; Verrucomicrobiae; | Verrucomicrobiae bacterium DG1235 | EDY80837 |
|  |  | WP_040900133 |
|  | *Verrucomicrobium* *spinosum* | WP_009960321 |
|  | *Pedosphaera* *parvula* Ellin514 | EEF62012 |

Supplementary Table S5. Statistical test showing a maximum likelihood analysis of G1PDH.

| Archaeal G1PDH (EgsA) | | | | | | | | Bacterial G1PDH (AraM) | | | au | np |
| --- | --- | --- | --- | --- | --- | --- | --- | --- | --- | --- | --- | --- |
| Crenarchaeota | | | | | | Thaum  -archaeota | Eury  -archaeota | *Bacillus* *subtilis* subsp. *subtilis* str. 168 | Deltaproteo  -bacteria  /Halo  -plasmatales  /*Anoxybacillus flavithermus* WK1 & *Bacillus cellulosilyticus* DSM 2522 | Gamma  -proteo  -bacteria  /Actino  -bacteria |  |  |
| Thermoproteales | | Desulfurococcales & Acidilobales | | | Sulfolobales |  |  |  |  |  |  |  |
| Most Thermo  -proteales | *Thermofilum pendens* Hrk-5 | Most Desulfuroco  -ccales | *Acidilobus saccharovorans*  /*Aeropyrum pernix* K1 | *Ignisphaera aggregans* DSM 17230 |  |  |  |  |  |  |  |  |
|  |  |  |  |  |  | X | X |  |  |  | 0.879 | 0.744 |
|  |  | Y | Y | Y | Y | Y | Y |  |  |  | 0.818 | 0.412 |
|  | Y | Y | Y | Y | Y | Y | Y | Y | Y | Y | **0.733** | 0.307 |
|  |  |  |  |  |  |  |  | X | X | X | 0.717 | 0.308 |
|  | X |  |  |  |  |  |  | X | X | X | 0.699 | 0.594 |
|  |  |  | Y | Y | Y |  |  |  |  |  | 0.697 | 0.731 |
|  |  |  | Y | Y |  |  |  |  |  |  | 0.697 | 0.731 |
|  |  |  |  |  |  |  |  |  | X | X | 0.674 | 0.425 |
| X | X |  |  |  |  |  |  | X | X | X | 0.604 | 0.135 |
|  | X |  |  |  |  |  |  | X |  |  | 0.599 | 0.249 |
|  |  |  |  |  |  |  |  | X | X |  | 0.589 | 0.271 |
| X |  |  |  |  |  | X | X |  |  |  | 0.476 | 0.193 |
|  | X |  |  |  |  | X | X | X | X | X | 0.454 | 0.071 |
| Y |  | Y | Y | Y | Y | Y | Y |  |  |  | 0.428 | 0.217 |
|  | Y | Y | Y | Y | Y | Y | Y |  |  |  | 0.413 | 0.166 |
| X | X |  |  |  |  | X | X | X | X | X | 0.391 | 0.081 |
|  | Y | Y | Y | Y | Y |  |  | Y | Y | Y | 0.39 | 0.102 |
|  |  |  |  |  |  |  |  | X |  | X | 0.376 | 0.114 |
|  | X |  |  |  |  |  |  |  | X | X | 0.372 | 0.06 |
|  | Y | Y | Y | Y | Y |  | Y | Y | Y | Y | 0.355 | 0.034 |
| Y | Y | Y | Y | Y | Y | Y | Y | Y |  |  | 0.35 | 0.155 |
| Y | Y | Y | Y | Y | Y | Y | Y |  |  | Y | 0.332 | 0.044 |
|  |  |  |  | X | X |  |  |  |  |  | 0.311 | 0.253 |
|  |  | Y | Y |  |  |  |  |  |  |  | 0.311 | 0.253 |
|  | X |  |  |  |  |  |  | X | X |  | 0.31 | 0.107 |
|  |  | Y | Y | Y | Y | Y |  |  |  |  | 0.305 | 0.054 |
|  | Y | Y | Y | Y | Y |  |  |  |  |  | 0.303 | 0.025 |
| Y | Y | Y | Y | Y | Y | Y | Y |  |  |  | **0.301** | 0.406 |
|  | X |  |  |  |  | X | X |  |  |  | 0.289 | 0.058 |
| Y | Y | Y | Y | Y | Y | Y | Y | Y |  | Y | 0.274 | 0.043 |
| Y | Y | Y | Y | Y | Y | Y | Y |  | Y | Y | 0.262 | 0.046 |
| X | X |  |  |  |  | X |  |  |  |  | 0.257 | 0.017 |
|  | X |  |  |  |  |  |  |  |  | X | 0.256 | 0.05 |
|  | X |  |  |  |  |  |  |  | X |  | 0.25 | 0.028 |
| Y | Y | Y | Y | Y | Y |  |  | Y | Y | Y | 0.25 | 0.026 |
|  | X |  |  |  |  |  |  | X |  | X | 0.231 | 0.057 |
| Y | Y | Y | Y | Y | Y | Y | Y | Y | Y |  | 0.23 | 0.059 |
| Y | Y | Y | Y | Y | Y | Y |  |  |  |  | 0.226 | 0.012 |
|  | X |  |  |  |  | X |  |  |  |  | 0.226 | 0.05 |
|  | X |  |  |  |  | X |  | X | X | X | 0.225 | 0.021 |
| X |  |  |  |  |  | X |  |  |  |  | 0.217 | 0.035 |
| Y |  | Y | Y | Y | Y |  |  |  |  |  | 0.213 | 0.033 |
|  | Y | Y | Y | Y | Y | Y |  | Y | Y | Y | 0.21 | 0.017 |
|  | Y | Y | Y | Y | Y | Y |  |  |  |  | 0.208 | 0.019 |
| Y | Y | Y | Y | Y | Y |  |  |  |  |  | 0.198 | 0.006 |
| X | X |  |  |  |  | X | X |  |  |  | 0.194 | 0.036 |
| Y |  | Y | Y | Y | Y |  | Y |  |  |  | 0.172 | 0.02 |
| X | X |  |  |  |  |  |  |  |  |  | 0.171 | 0.02 |
|  |  | Y | Y | Y | Y |  | Y |  |  |  | 0.163 | 0.046 |
| Y | Y | Y | Y | Y | Y | Y | Y |  | Y |  | 0.159 | 0.021 |
|  | X |  |  |  |  |  | X |  |  |  | 0.159 | 0.008 |
|  | X |  |  |  |  |  | X | X | X | X | 0.127 | 0.012 |
| Y | Y | Y | Y | Y | Y | Y |  | Y | Y | Y | 0.117 | 0.005 |
| Y | Y | Y | Y | Y | Y |  | Y | Y | Y | Y | 0.116 | 0.009 |
| X |  |  |  |  |  |  | X |  |  |  | 0.113 | 0.028 |
|  | Y | Y | Y | Y | Y |  | Y |  |  |  | 0.103 | 0.003 |
| Y | Y | Y | Y | Y | Y |  | Y |  |  |  | 0.101 | 0.002 |
| X | X |  |  |  |  | X |  | X | X | X | 0.096 | 0.001 |
| X | X |  |  |  |  |  | X | X | X | X | 0.095 | 0.001 |
| Y |  | Y | Y | Y | Y | Y |  |  |  |  | 0.094 | 0.005 |
| X | X |  |  |  |  |  | X |  |  |  | 0.016 | 2.00E-04 |

The AU test [34] was performed using Consel v0.1j [35] to test various alternative phylogenetic hypotheses. Based on the ML tree of G1PDH inferred by the RAxML, we divided G1PDHs into 8 groups, *Thermofilum pendens* Hrk-5 (Thermoproteales of Crenarchaeota) (A), Most Thermoproteales (rest of Thermoproteales) (B), Desulfurococcales + Acidilobales + Sulfolobales (C), Thaumarchaeota (D), Euryarchaeota (E), *Bacillus* *subtilis* subsp. *subtilis* str. 168 (F), Deltaproteobacteria + Haloplasmatales + *Anoxybacillus flavithermus* WK1 + *Bacillus cellulosilyticus* DSM 2522 (G), and Gammaproteobacteria + Actinobacteria (H), together with outgroup (O). Under the two constraint conditions ({{A, F, G, H}, B, C, D, E, O} and {A, B, C, D, E, {F, G, H, O}}), we listed 3,150 relationships among 8 G1PDH groups and 1 outgroup, using ProtML of Molphy 3.2b [36]. Next, the 3,150 relationships were used as the constraint for an ML tree search performed with RAxML with the PROTGAMMALG model. The log-likelihoods of 3,150 resultant trees were compared, and the top 2,000 trees on the log-likelihoods were then used for the AU test with Consel. The species (or groups) with white columns form a group together with the outgroup. Those with red columns form a distinct subgroup within the group including the outgroup (white columns).
