# Supplementary material for: Birth of Archaeal Cells: Molecular Phylogenetic Analyses of G1P Dehydrogenase, G3P Dehydrogenases, and Glycerol Kinase Suggest Derived Features of Archaeal Membranes Having G1P Polar Lipids
Source: Archaea. 2016 Sep 28;2016:1802675. doi: 10.1155/2016/1802675 (PMC5059525; doi:10.1155/2016/1802675)
Supplement: Supplementary file 1 — Supplementary Table S1: The list of sequence entries used to infer the G1PDH (EgsA/AraM) tree. Supplementary Table S2: The list of sequence entries used to infer the G3PDH (GpsA) tree. Supplementary Table S3: The list of sequence entries used to infer the G3PDH (GlpA/D) tree. Supplementary Table S4: The list of sequence entries used to infer the GK (GlpK) tree. Supplementary Table S5: Statistical test showing a maximum likelihood analysis of G1PDH. The AU test [34] was performed using Consel v0.1j [35] to test various alternative phylogenetic hypotheses. Based on the ML tree of G1PDH inferred by the RAxML, we divided G1PDHs into 8 groups, Thermofilum pendens Hrk-5 (Thermoproteales of Crenarchaeota) (A), Most Thermoproteales (rest of Thermoproteales) (B), Desulfurococcales + Acidilobales + Sulfolobales (C), Thaumarchaeota (D), Euryarchaeota (E), Bacillus subtilis subsp. subtilis str. 168 (F), Deltaproteobacteria + Haloplasmatales + Anoxybacillus flavithermus WK1 + Bacillus cellulosilyticus DSM 2522 (G), and Gammaproteobacteria + Actinobacteria (H), together with outgroup (O). Under the two constraint conditions ({{A, F, G, H}, B, C, D, E, O} and {A, B, C, D, E, {F, G, H, O}}), we listed 3,150 relationships among 8 G1PDH groups and 1 outgroup, using ProtML of Molphy 3.2b [36]. Next, the 3,150 relationships were used as the constraint for an ML tree search performed with RAxML with the PROTGAMMALG model. The log-likelihoods of 3,150 resultant trees were compared, and the top 2,000 trees on the log-likelihoods were then used for the AU test with Consel. The species (or groups) with white columns form a group together with the outgroup. Those with red columns form a distinct subgroup within the group including the outgroup (white columns). Supplementary Figure S1: The trimed multiple alignment used for the phylogenetic analyses of G1PDH (EgsA/AraM). Details how to create this alignment is found in section 2.1 of main text. Supplementary Figure S2. Alignment of G1PDH (Egs [file 1802675.f1.zip › Supplementary_Materials_Yokobori_et_al_part_2_ARCH_1737173.pdf]

Legends to Supplementary Figures

Supplementary Figure S1: The trimed multiple alignment used for the phylogenetic analyses of G1PDH (EgsA/AraM). Details how to create this alignment is found in section 2.1 of main text.

Supplementary Figure S2. Alignment of G1PDH (EgsA/AraM) with selected sequences. The alignment is the subset of alignment used for the phylogenetic analysis. Sto: *Sulfolobus tokodaii* str. 7, DDBJ/GenBank/EMBL accession No. P58460. Afu: *Archaeoglobus fulgidus* DSM 4304, NP_070502. Msm: *Methanobrevibacter smithii* DSM 2374, ZP_05976397. Sco: *Streptomyces coelicoflavus* ZG0656, EHN78548. Bsu: *Bacillus subtilis* subsp. *subtilis* str. 168, NP_390754.

Supplementary Figure S3: The trimed multiple alignment used for the phylogenetic analyses of G3PDH (GpsA). Details how to create this alignment is found in section 2.2 of main text.

Supplementary Figure S4. Alignment of G3PDH (GpsA) with selected sequences. The alignment is the subset of alignment used for the phylogenetic analysis. Asu: *Archaeoglobus sulfaticuallidus*, DDBJ/GenBank/EMBL accession No. WP_015591014. Mru: *Methanobrevibacter ruminantium*. WP_012956986, Bsu: *Bacillus subtilis* subsp. *subtilis* str. 168, AAA86746. Eco: *Escherichia coli* str. K-12 substr. MG1665, AAB18585. Tth: *Thermus thermophilus* HB8, Q5SHJ0.

Supplementary Figure S5: The trimed multiple alignment used for the phylogenetic analyses of G3PDH (GlpA/GlpD). Details how to create this alignment is found in section 2.3 of main text.

Supplementary Figure S6. Alignment of G3PDH (GlpA/GlpD) with selected sequences. The alignment is the subset of alignment used for the phylogenetic analysis. Sso: *Sulfolobus solfataricus* P2, DDBJ/GenBank/EMBL accession No. NP_343866. Afu: *Archaeoglobus fulgidus* DSM 4304, NP_070157. Mar: *Methanocella arvoryzae* MRE50. YP_687586. Eco_GlpA: *Escherichia coli* str. K-12,

P0A9C0 (GlpA). Eco_GlpD: *Escherichia coli*, 2R4J_A (GlpD). Tth: *Thermus thermophilus* HB8, YP_145382. Bsu: *Bacillus subtilis* subsp. *subtilis* str. 168, ZP_03590616.

Supplementary Figure S7: The trimed multiple alignment used for the phylogenetic analyses of GK (GlpK). Details how to create this alignment is found in section 2.4 of main text.

Supplementary Figure S8. Alignment of glycerol kinase (GlpK) with selected sequences. The alignment is the subset of alignment used for the phylogenetic analysis. Afu: *Archaeoglobus fulgidus* DSM 4304, DDBJ/GenBank/EMBL accession No. AAB90370. Sac: *Sulfolobus acidocaldarius* DSM 639, AAY80469. Bsu: *Bacillus subtilis* subsp. *subtilis* str. 168, AIY92216. Eco: *Escherichia coli* str. K-12, AAB03058. Tth: *Thermus thermophilus*, BAA28283.

Supplementary Figure S9: Detailed version of the G1PDH (EgsA/AraM) tree (ML method). This tree is a detailed version of the tree presented in Figure 3 in the main text. See detailed descriptions in the legend of Figure 3. At each node, the supporting bootstrap probability (BP) is shown (%).

Supplementary Figure S10: Detailed version of G1PDH (EgsA/AraM) the tree (BI method). The tree was constructed using PhyloBayes version 3.2f [32] under the CAT-Poisson (C20) + G (4) model. The alignment with 182 OTUs and with 252 sites without any indels was used. 200,000 MCMC cycles were performed, the sampling rate was every 10 cycles, and the first 50,000 cycles (5,000 sampled cycles) were discarded for further analysis. The log marginal likelihood of this tree is -45503.8±17.3. The Posterior probability (PP) is shown at each node of the tree.

Supplementary Figure S11: Detailed version of the G3PDH (GpsA) tree (ML method). This tree is a detailed version of the tree presented in Figure 3 in the main text. See detailed descriptions in the legend of Figure 3. At each node, the supporting BP is shown (%).

Supplementary Figure S12: Detailed version of the G3PDH (GpsA) tree (BI method). The tree was constructed using PhyloBayes version 3.2f [32] under the CAT-Poisson (C20) + G (4) model. The alignment with 305 OTUs and with 84 sites without any indels was used. 200,000 MCMC cycles were performed, the sampling rate was every 100 cycles, and the first 40,000 cycles (400 sampled cycles) were discarded for further analysis. The log marginal likelihood of this tree is -29860.5±27.3. The PP is shown at each node of the tree.

Supplementary Figure S13: Detailed version of the G3PDH (GlpA/GlpD) tree (ML method). This tree is a detailed version of the tree presented in Figure 3 in the main text. See detailed descriptions in the legend of Figure 3. At each node, the supporting BP is shown (%).

Supplementary Figure S14: Detailed version of the G3PDH (GlpA/GlpD) tree (BI method). The tree was constructed with PhyloBayes version 3.2f [32] under the CAT-Poisson (C20) + G (4) model. The alignment with 282 OTUs and with 239 sites without any indels was used. 200,000 MCMC cycles were performed, the sampling rate was every 100 cycles, and the first 40,000 cycles (400 sampled cycles) were discarded for further analysis. The log marginal likelihood of this tree is -70373.3±26.0. The PP is shown at each node of the tree. Six monophyletic subgroups of GlpA/GlpD are noted as A1 and A2 for archaeal groups and B1 to B4 for bacterial groups.

Supplementary Figure S15: Detailed version of the GK (GlpK) tree (ML method). This tree is a detailed version of the tree presented in Figure 3 in the main text. See detailed descriptions in the legend of Figure 3. At each node, the supporting BP is shown (%).

Supplementary Figure S16: Detailed version of the GK (GlpK) tree (BI method). The tree was constructed with PhyloBayes version 3.2f [32] under the CAT-Poisson (C20) + G (4) model. The alignment with 374 OTUs and with 194 sites without any indels

was used. 200,000 MCMC cycles were performed, the sampling rate was every 100 cycles, and the first 50,000 cycles (500 sampled cycles) were discarded for further analysis. The log marginal likelihood of this tree is -77268.3±29.8. The PP is shown at each node of the tree.
