# Supplementary material for: Birth of Archaeal Cells: Molecular Phylogenetic Analyses of G1P Dehydrogenase, G3P Dehydrogenases, and Glycerol Kinase Suggest Derived Features of Archaeal Membranes Having G1P Polar Lipids
Source: Archaea. 2016 Sep 28;2016:1802675. doi: 10.1155/2016/1802675 (PMC5059525; doi:10.1155/2016/1802675)
Supplement: Supplementary file 1 — Supplementary Table S1: The list of sequence entries used to infer the G1PDH (EgsA/AraM) tree. Supplementary Table S2: The list of sequence entries used to infer the G3PDH (GpsA) tree. Supplementary Table S3: The list of sequence entries used to infer the G3PDH (GlpA/D) tree. Supplementary Table S4: The list of sequence entries used to infer the GK (GlpK) tree. Supplementary Table S5: Statistical test showing a maximum likelihood analysis of G1PDH. The AU test [34] was performed using Consel v0.1j [35] to test various alternative phylogenetic hypotheses. Based on the ML tree of G1PDH inferred by the RAxML, we divided G1PDHs into 8 groups, Thermofilum pendens Hrk-5 (Thermoproteales of Crenarchaeota) (A), Most Thermoproteales (rest of Thermoproteales) (B), Desulfurococcales + Acidilobales + Sulfolobales (C), Thaumarchaeota (D), Euryarchaeota (E), Bacillus subtilis subsp. subtilis str. 168 (F), Deltaproteobacteria + Haloplasmatales + Anoxybacillus flavithermus WK1 + Bacillus cellulosilyticus DSM 2522 (G), and Gammaproteobacteria + Actinobacteria (H), together with outgroup (O). Under the two constraint conditions ({{A, F, G, H}, B, C, D, E, O} and {A, B, C, D, E, {F, G, H, O}}), we listed 3,150 relationships among 8 G1PDH groups and 1 outgroup, using ProtML of Molphy 3.2b [36]. Next, the 3,150 relationships were used as the constraint for an ML tree search performed with RAxML with the PROTGAMMALG model. The log-likelihoods of 3,150 resultant trees were compared, and the top 2,000 trees on the log-likelihoods were then used for the AU test with Consel. The species (or groups) with white columns form a group together with the outgroup. Those with red columns form a distinct subgroup within the group including the outgroup (white columns). Supplementary Figure S1: The trimed multiple alignment used for the phylogenetic analyses of G1PDH (EgsA/AraM). Details how to create this alignment is found in section 2.1 of main text. Supplementary Figure S2. Alignment of G1PDH (Egs [file 1802675.f1.zip › Supplementary_Materials_yokobori_et_al_part_3_ARCH_1737294.docx]

Supplementary figure S1

Supplementary figure S2
